# Investing in youth public mental health in India: multi-stakeholder co-production of a whole school program to promote the mental health of Indian adolescents

**DOI:** 10.3389/fpubh.2025.1670439

**Published:** 2025-11-28

**Authors:** Siobhan Hugh-Jones, Sphoorthi Prabhu, Muthuraju Arelingaiah, Jayalaxmi Podiya, Krupa Lakshman, Ritwika Nag, Lucy Warriner, Amy Palmer, Kristian Hudson, Mina Fazel, Surendran Ventakaraman, Paul Cooke, Pavan Mallikarjun, Prachi Khandeparkar, Poornima Bhola, Janardhan Navaneetham

**Affiliations:** 1 School of Psychology, University of Leeds, Leeds, United Kingdom; 2 National Institute of Mental Health and Neurosciences, Bangalore, India; 3 Improvement Academy, Institute for Health Research, Bradford, United Kingdom; 4 Department of Psychiatry, University of Oxford, Oxford, United Kingdom; 5 Indira Gandhi Medical College and Research Institute, Government of Puducherry Institution, Puducherry, India; 6 School of Languages, Cultures and Societies, University of Leeds, Leeds, United Kingdom; 7 Birmingham Women's and Children's NHS Foundation Trust, Birmingham, United Kingdom; 8 Sangath, New Delhi, India

**Keywords:** adolescent mental health, India, anxiety, depression, intervention, co-design, whole school approach

## Abstract

**Introduction:**

In India, although rates of adolescent anxiety and depression are of concern and contribute to a high youth suicide rate, public mental health approaches targeting adolescents are rare. India does not have an integrated whole-school mental health approach to promote youth wellbeing and prevent anxiety and depression. The aim of this study (Project SAMA) was to co- produce a whole school mental health program for delivery in Indian secondary schools.

**Method:**

Using the ADAPT framework, we conducted umbrella and systematic reviews to identify school interventions with proven effectiveness which could be culturally adapted to India. Adopting a whole school approach, we sought evidence for interventions targeting risk and protective factors at the level of adolescents, teachers, parents and school climate. Informed by guidance from our Indian Youth Advisory Board and Scientific Advisory Boards, we built on the generally low availability of evidence-based interventions, drawing where possible on broader evidence of what works, to generate prototype interventions to take to co-adaptation and co-production with local communities. Working with 57 Indian stakeholders, spanning adolescents, parents, teachers, head teachers and mental health professionals, we reached consensus on a whole school program ready for feasibility testing, including how the program should be implemented and evaluated, and how safeguarding should be operationalized.

**Results:**

We report our final program content and implementation plan ready for feasibility testing. The program consists of four inter-related interventions. These target protective factors for adolescent mental health, including youth, teacher and parent mental health literacy and psychoeducation, a positive and safe school culture, and psychosocial support. They also target the mental health risk factors of stigma, bullying and corporal or harsh discipline. Agreed delivery agents are lay counsellors and local mental health professionals. We report TIDieR checklists and logic models for each intervention and an integrated program Theory of Change.

**Discussion:**

The final program, which reflects six of the World Health Organization's and UNESCO's eight global standards for health promoting schools, is freely available. Project SAMA makes a significant contribution to our understanding of what adolescents, school and parents want from a whole school approach to public mental health in India.

## 1 Introduction

Globally, and in India, more than half of mental health conditions arise during adolescence. When last assessed in 2016, an estimated 9.8 million Indian 13–17-year-olds (pooled prevalence of 7.3%) had a mental health condition (as per ICD-10 diagnostic criteria) (1) although other studies suggest considerably higher prevalence (23%) among school-going adolescents (11–18 years) (2). Anxiety and depression are the most common mental health conditions globally, and in India, are the leading causes of disability among adolescents. Sub-clinical symptoms of anxiety and depression are more common in adolescents than clinical threshold symptoms (3), although they are a risk factor for escalation to clinical levels of disorder (4). Adolescent anxiety and depression are associated with poor physical health and lifelong disadvantage (5) and can be precursors to suicide (6). India has one of the highest suicide rates globally in those aged between 15 and 19 years (7, 8). Without action, the prevalence of mental health disorders in India is set to rise. Early evidence-based intervention is integral to reducing mental distress and promoting positive mental health and resilience in at-risk adolescents (9).

Globally, there has been an exponential interest in prevention approaches for mental health conditions (10). Prevention can operate at three levels: primary prevention to avert a disorder prior to any symptoms; secondary prevention, to lessen the progression to disorder when symptoms appear; and tertiary prevention, to lessen the impact of a disorder once it has commenced. In line with global calls for action on mental health (11), some Indian policies recognize the scale of the problem and advocate investment in prevention to improve population levels of mental health (12–14). Primary and secondary prevention have the potential to influence adolescent mental health in India given the treatment gap and under-resourced mental healthcare systems (1).

The Health Promoting Schools Framework (HPSF) (15) has driven attention to schools as sites to advance population health. Organized efforts to implement the HPSF in India have been mixed, and largely sub-optimal, opting to focus mostly on large-scale screening programs (16, 17). However, new focus is emerging around public mental health in communities, including schools. Such investment is warranted for three key reasons. First, the Indian school systems is one of the largest in world (17). Although the majority of adolescents do not suffer from mental health conditions and have a relatively positive experience in school, school life in India (and globally) can present mental health risks to students. Such risks include: the use of corporal punishment (18); extreme academic pressure (19, 20); poor mental health literacy among adolescents and teachers (21–24); prevailing mental health stigma and bullying (25); poorly supported staff, often with large class sizes and a general lack of mental health support within schools (26). In line with prevention science, addressing these risks has the potential to reduce the prevalence of youth mental health conditions, including anxiety and depression (27–30). Most mental health conditions arise from multiple causes meaning that prevention approaches should target multiple risk and protective factors simultaneously (30). Both young people with sub-clinical or clinical levels of mental health disorder can benefit from prevention interventions (30).

Second, Caldwell et al.'s (31) systematic review of school-based interventions to prevent anxiety and depression in children and young people concluded that future preventive interventions should not focus solely on individual protective or risk factors but also those that reside in the systems surrounding the adolescent (32). Globally, there is emerging consensus that mental health work in schools needs to expand from targeting only the vulnerable adolescent (e.g., by offering school counseling) to also targeting risk and protective factors that are relational (e.g., reducing bullying, fostering relationship skills), environmental (e.g., school climate, stigma) and organizational (e.g., school policies and processes, teacher training) (33). Whilst there has been good investment into school life skills program in India (34–36), these approaches still individualize mental health, do not address a broad range of risk and protective factors, and lack evidence of effectiveness.

Third, although there are many unsuccessful school mental health programs (31, 37), some are effective, cost-effective (38–41) and worth pursing (42). Evidence on their effectiveness in preventing anxiety and depression) is promising but limited to date (30). Their advantage is their potential to reach, without stigmatizing, large numbers of adolescents with varying symptomology who would typically have limited access to treatment or support (43). Whole school programs are often financially viable, have low dropout (43–46) and can target multiple risk and protective factors simultaneously (47–49) whilst fostering outcomes that also matter to educational institutions, e.g., attendance and academic success (50, 51). Parikh et al. (26) identified that, in India, stakeholder appetite for school mental health programs is strong, and the Government of India, in its National Health Policy (Ministry of Health and Family Welfare) (12) and National Education Policy (52) identified schools as key locations for improving the physical and mental health of adolescents through the provision of information and health services.

There are school initiatives in India which could be seen as supporting adolescent mental health. Two operate at a national level: the Ayushman Bharat program focuses on neurodevelopmental disorders, substance abuse, internet safety, yoga, and meditation (53), and the National and District Mental Health Programs recommend school outreach activities with a focus on suicide prevention (54). Several states have generated regional initiatives and programs for adolescent wellbeing. However, many of these remain untested, are opaque with regards to their theoretical or empirical basis, are poorly implemented and lack sustainability (55). Only four whole-school health and life-skills programmes with small mental health components have been tested in India. Two did not report any clinical outcomes for psychological symptoms (36) and one has yet to establish effectiveness (56) indicating that life-skills interventions may be insufficient to limit the onset and progression adolescent anxiety and depression. The third is a health promotion programme (SEHER; Strengthening Evidence base on scHool-based intErventions for pRomoting adolescent health) which focused on physical and sexual health, bullying, gender equality and depressive symptoms and emphasized the mediating role of school climate in student health outcome (57). School climate is a multidimensional construct representing subjective experiences of the school particularly in relation to psychological safety, belonging, relationships and engagement, and can be associated with student mental health (58). SEHER promoted a multi-activity approach, spanning whole-school, class and individual components including school assemblies, school competitions, wall magazines, speak out boxes and a school health promotion committee. Following identification of best delivery agents (59), SEHER was delivered in Bihar, India by lay counselors (60). Outcomes were positive, including for school climate and depressive symptoms, with benefits observed for up to 2 years, making a strong case for multi-component approaches that include school climate (57, 59).

SEHER demonstrates many elements of the HPSF (15) and, although not formally theorized as such, reflects aspects of the theory of human functioning and school organization which sees schools exerting positive influence through promoting practical reasoning (for health) and capacity for affiliation (positive relationships, shared values) (60, 61). SEHER did not, however, target anxiety nor other risk or protective factors in the system. This is a critical gap given the high prevalence of anxiety disorders in adolescence, its co-morbidity with depression, the promising effects found for psychological/educational anxiety prevention programs (10, 62) and the importance of taking a whole school approach which includes teachers and parents as synergistic influences on adolescent wellbeing and the need to “erode boundaries” (61). Our study aimed to progress resources and evidence for whole school approaches to adolescent mental health in Indian secondary schools. This paper describes the co-creation of our SAMA program and its conceptual framework ready for feasibility testing.

Project SAMA was a collaboration between the National Institute of Mental Health and Neuroscience in Bengaluru (NIMHANS, India), the University of Leeds (UK) and several other Indian and UK partners. Our team was interdisciplinary, with shared decision making, intellectual leadership, and responsibility for project delivery. Appointed research staff in India and the UK were typically paired for joint tasks (e.g., systematic reviewing). The India team led the fieldwork, and the UK team led plans for data management, analysis, and integration. Our end goal was to feasibility test a co-designed whole school wellbeing program for delivery in Indian secondary schools to inform a future effectiveness trial [see Hugh-Jones et al. (63) for feasibility study protocol]. We use the term “wellbeing” to encompass the program's potential to help the wellbeing of all adolescents exposed to the program, although our primary focus is on prevention and early intervention for those symptomatic of anxiety and/or depression. We envisaged the program would be a complex intervention (64) and conceived of schools as social complex adaptive systems (65). We describe in Supplementary File 1 steps 1–3 of the Six Steps in Quality Intervention Development (6SQuID) (66) which outlines the nature of the problem and its causes, along with which causal or contextual factors we believed would be most amenable to change. This paper describes the development of the SAMA program and its conceptual framework ready for feasibility testing and is structured around our use of guidance for adapting interventions to new contexts and for deciding when creation of a new intervention is warranted (ADAPT) (67).

For clarity, henceforth, we refer to our plans for a whole school *program* with interrelated *interventions* each with *components*. Our primary outcome for the eventual program was youth anxiety and depression [as per our study protocol (63)]. We planned to build on SEHER, extending the focus to adolescent anxiety, to target teachers and parents as key system actors, and to deliver a more comprehensive intervention of school climate improvement. We focused on these four targets (adolescents, teachers, parents, and school climate) as representative of individual, relational, and contextual influences on adolescent mental health that could be addressed via a whole school promotion and prevention program (59, 63). Our intentions were to positively disrupt proximal risky school and individual contexts, processes and/or mechanisms which were likely to be of relevance to adolescent anxiety and depression, and to promote those important to wellbeing (32). Programs aiming to induce change in social complex adaptive systems like schools need multilevel, synergistic action. Our a priori theoretical lens was Markham and Aveyard's (61) theory of health promoting schools which posits that being in a position to choose to function well and flourish is necessary to maximize one's health potential, acknowledging that for adolescent mental health this potential is subject to multiple influences. The theory proposes that schools can promote autonomy to function well via school organization, curriculum, and pedagogic practices.

In line with ADAPT (67), we planned to first identify any existing whole school programs with established effectiveness for: (i) the reduction of anxiety and depression symptoms in adolescents; (ii) improvements in teachers' mental health literacy and teachers' use of positive practices for behavior management; (iii) improvements in school climate (including reductions in bullying); and (iv) improvements in parents' mental health literacy. If candidate programs/interventions existed, we then planned to culturally co-adapt these to the local context with stakeholders. Cultural co-adaptation is “the systematic modification of an evidence-based treatment or intervention protocol to consider language, culture, and context in such a way that is compatible with the client's cultural patterns, meanings, and values” (68). Such practice is critical to enhancing the cultural relevance and future success of interventions (69). Where no suitable program/intervention existed, we planned to co-produce these with local school communities and stakeholders (63). Co-production of school mental health programs remains rare globally, and in India.

## 2 Materials and methods

### 2.1 Ethical approvals

Ethical approval for this study was granted by the Indian Council of Medical Research and the institutional ethics review board of the University of [blinded for review] Faculty of Medicine & Health (UK) and the National Institution of Mental Health and Neurosciences (India) Ethics Committee, Behavioral Science Division. Local approval was obtained from the Ministry of Primary and Secondary Education (Government of Karnataka), the Departments of Public Instruction, and the State Educational Research and Training (DSERT).

### 2.2 Setting

Project SAMA was delivered in the state of Karnataka (India) as it is the location for NIMHANS and their effective relationships across local government and school communities. Karnataka is the 8th largest state by population and its capital is Bengaluru. Literacy rates in Karnataka are high [82.47% of males and 68% of females are literate; 2011 census (70)]. NIMHANS is a government-funded center of excellence in Bengaluru providing child, adolescent and adult mental health (primary to tertiary care). Many other institutes and non-governmental organizations also provide outpatient and community mental health services in the region. School mental health programs are delivered in Karnataka state government schools as a part of the District Mental Health Program under the National Mental Health Program (71) and Rashtriya Kishor Swasthya Karyakram (13). The Central Board of Secondary Education in India mandates its schools to integrate life skills education into the school curriculum and for each school to have a school counselor. However, most of these programs are not delivered consistently and fail to reach most school-going children (54). In our study, we worked with stakeholders from Bengaluru (urban area) and Kolar (rural area).

### 2.3 ADAPT framework methodology

**Figure 1:**
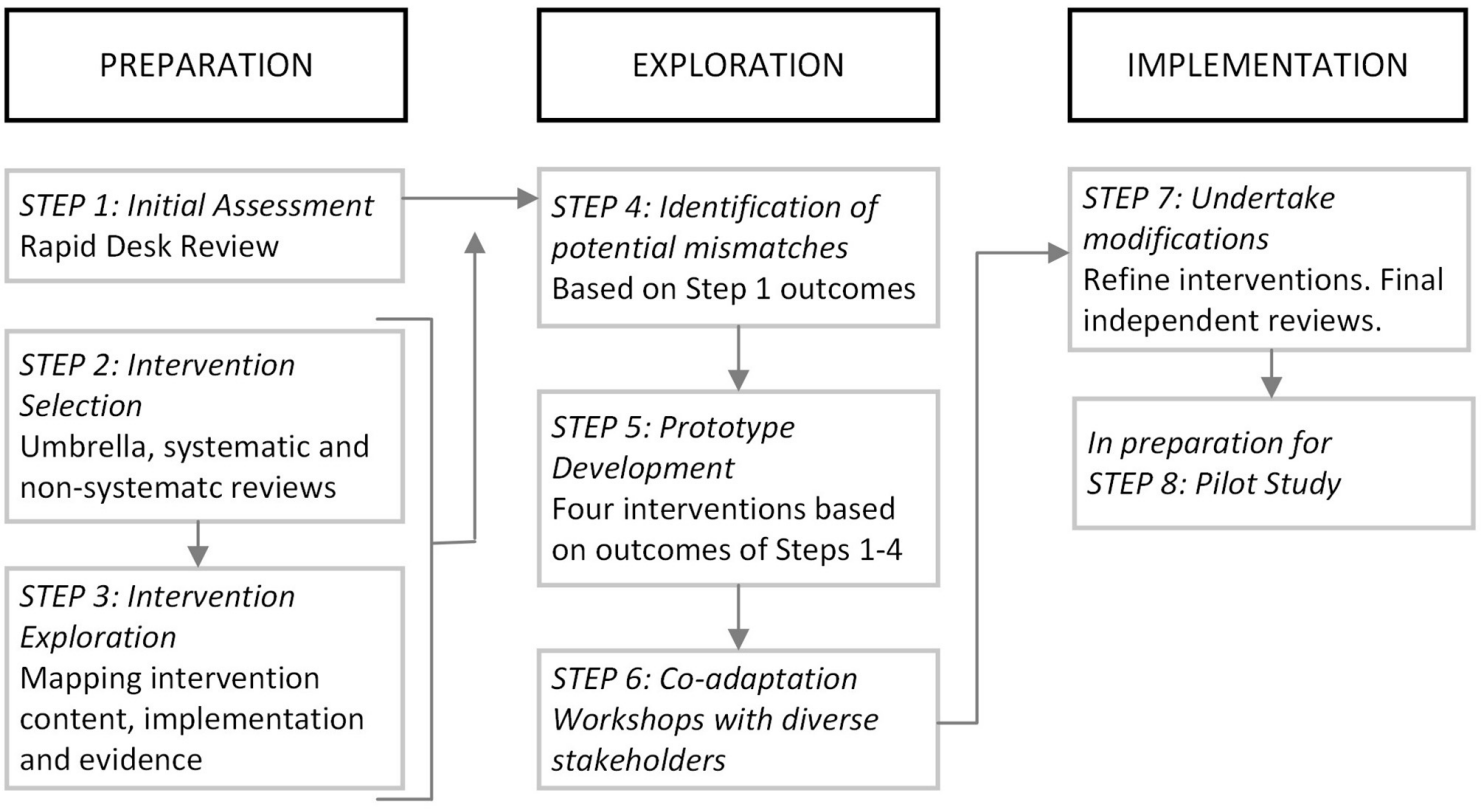
Mapping of project SAMA stages to the ADAPT framework (69).

Our work followed the Adapt framework, specifically Movsisyan et al.'s (69) steps 1–7 for adapting complex population health interventions to new contexts and Moore et al.'s (67) decision pathways for when new interventions or new intervention components should be developed for new contexts. The Adapt steps move from intervention exploration and adaptation to preparation to implementation. Figure 1 shows how our phases of work aligned with these steps. Additional frameworks were drawn upon at key points and are explained below.

#### 2.3.1 ADAPT step 1: initial assessment

We conducted a rapid desk review to identify key features of Indian culture, schooling, and national priorities to which our program should be sensitized [e.g., (26, 72)]. Desk reviews and local needs assessments support balance between globally evidenced interventions and local culture, priorities, and practices (73) and should be pragmatic to serve a project's aim (74). We mapped the education system and its decision-makers, existing life skills school programs, the state child and adolescent mental health system and potential for future intervention commissioning and funding, and relevant national policies [e.g., (12, 75, 76)]. Local needs assessments (relevant to Bengaluru and Kolar) were conducted delineating key community practices, values, expectations and possibilities with regards to the delivery of programs in schools. We also documented local vocabularies around mental health and wellbeing.

#### 2.3.2 ADAPT step 2: intervention selection

Our aim was to identify candidate interventions, with established effectiveness in other countries or contexts, that with local co-adaptation, were likely to be acceptable, feasible and efficacious in our target context to meet our project aims. Four umbrella reviews were conducted to identify international or Indian interventions published between January 2011 until January 2022 with evidence of effectiveness in domains relevant to our program, namely, for: (i) reducing, via a universal school approach, symptoms of anxiety and/or depression in school-going adolescents; (ii) improving secondary school teachers' mental health literacy; (iii) improving school climate; and (iii) improving parents' mental health literacy. Given the low prevalence of studies providing evidence, we conducted a non-systematic review of programs to improve teachers' use of positive teaching practices. Reviews were pre-registered and our search strategies are reported in Supplementary File 2. We topped up with manual searches for studies conducted after the last systematic reviews in each domain were published. Interventions were eligible if they had established effectiveness with high quality evidence, were freely available, and were in principle feasible for implementation and scaling in the local context.

#### 2.3.3 ADAPT step 3: intervention exploration

For all candidate interventions, we attempted to source intervention manuals. Using a reporting template, we comprehensively documented each intervention and underpinning theory, content, implementation, evidenced or hypothesized mechanisms and moderators of change.

#### 2.3.4 ADAPT step 4: identification of potential mismatches

The India-UK team collaboratively considered “potential mismatches” (at surface and deep levels) (77) between candidate interventions and both the local needs identified in Step 1 and our overall aims for each intervention domain. We also considered potential unintended consequences of each intervention in our target context as well as cost implications and resource availability (67).

#### 2.3.5 ADAPT step 5: intervention model development

Based on the outcomes of Steps 1–4, and in preparation for co-adaptation with stakeholders, we developed prototypes of four interventions representing our four intervention domains (i.e., youth, teachers, school climate and parents). We began by mapping intervention content and the logic or theory of change for each intervention, established from steps 1–5. Each prototype was then evaluated by the Indian team using Perera et al.'s (74) approach to cultural adaptation of psychosocial interventions which involves consideration of the eight dimensions of the Ecological Validity Model (EVM), namely language, persons, metaphors, content, concepts, goals, methods, and context (68, 78). As per guidance on the development of complex interventions (79), we created working logic models for the four interventions to help communicate hypothesized mechanisms to stakeholders, but which were open to change following co-adaptation. Once prototypes were complete, we generated adaptation hypotheses. These were research team statements regarding likely sources of cultural non-fit (74). Co-adaptation workshops focused on areas of greatest uncertainty about cultural fit. Key principles of intervention development are that it is dynamic, iterative, creative, open to change, and forward looking to future evaluation and implementation (79). We simultaneously considered implementation and suitability for evaluation.

#### 2.3.6 ADAPT step 6: co-adaptation/co-production

Our Step 6 differed from the ADAPT model as we delivered an intense phase of working with local stakeholders on intervention co-adaptation and co-production to optimize the local acceptability, feasibility, and efficacy of the prototype interventions and implementation proposals (focusing on our adaptation hypotheses) and to co-produce new components where necessary. It was decided in advance that as much of the program as possible would be delivered by lay counselors as their acceptability and effectiveness in delivering school wellbeing programs in India, compared to teachers as delivery agents, was established in the SEHER trial (59). We also aimed to co-produce with stakeholders locally appropriate and meaningful safeguarding, data management and evaluation protocols for the feasibility stage.

##### 2.3.6.1 Step 6 methodology

###### 2.3.6.1.1 Participants

Co-production was conducted in line with Lundy's (80) model for participation of children and young people in decision-making which includes (i) Space: children must be given the opportunity to express a view; (ii) Voice: children must be facilitated to express their views; (iii) Audience: the view must be listened to; and (iv) Influence: the view must be acted upon, as appropriate. At project start, we established a Youth Advisory Board (YAB) informed by the Global Consensus Statement on Meaningful Adolescent (PMNCH) and Youth Participation (81) and the World Health Organization Toolkit for Adolescents Advocating Change [PMNCH (82)]. Our YAB comprised 17 local adolescents between 14 and 17 year (12 females and 5 males) who were involved in multiple aspects of project direction and delivery and met 11 times over the course of the study. Recruitment of more girls than boys on our YAB was purposeful to redress disparities in inclusion of girls in such opportunities (83). We also strove to recruit adolescents with different gender identities but were unsuccessful. Full details of our YAB and their influence on this project is detailed elsewhere (84).

For the co-adaptation workshops, we aimed to recruit a minimum of 50 participants spanning adolescents, parents, head-teachers, teachers, and mental health professionals. Recruitment was via direct invitations from the NIMHANS research team across their local networks of private and government-funded schools. Permission to recruit schools was given by the Commissioner of Public instruction (Government of Karnataka). We met with school leadership in urban and rural schools across Bengaluru and Kolar which had over 200 students and focused on government schools where most Indian adolescents are enrolled but where resources are limited. Inclusion of schools in the study was contingent on the willingness of school leadership to participate, following which teacher, student and parent recruitment was conducted. There were no eligibility criteria, but we encouraged participation by those who were interested in adolescent wellbeing. As per our protocol (63), we only recruited adolescents from rural schools to ensure we worked most intensely with under-represented young people. All potential participants (adolescents, teacher, parents) and guardians received study information letters. Informed assent (young people) and consent (adults) was secured prior to study start. We were attentive to the likely varied levels of literacy among adolescents and parents/carers (85). Study information letters were provided in English and in Kannada (the dominant regional language) and was crafted by the Indian team with extensive experience of working in local schools. The Indian team also attended schools in person to provide verbal explanations of the study and the information letters. Although we had higher representation of girls than boys on our YAB, we aimed for equal gender representation in co-design workshops in recognition of the need to develop a program that was gender sensitive. Travel expenses were reimbursed (including for accompanying guardians). Participants were provided with certificates of participation for their time and contributions.

We recruited 57 stakeholder participants. Table 1 shows participant and workshop details. We had representation from four schools (both government and private). Mental health professionals were psychiatrists, clinical psychologists and psychiatric social workers and two representatives from NGOs who worked in school health and mental health.

**Table 1:** Co-adaptation workshop participant demographics (*N* = 57).

**Stakeholder group**	***N***	**Gender**	**Location**	**Number of workshops**	**Workshop duration**
Adolescents	7	All female	Rural Kolar	2	4 h each
Adolescents	8	All male	Rural Kolar	2	4 h each
Parents	13	6M; 7F	Rural Kolar	1	3 h
Head teachers	9	5M; 4F	Rural Kolar and Urban Bangalore	2	2 h + 5 h
Teachers	10	2M; 8F	Rural Kolar and Urban Bangalore	1	4 h
Mental health professionals	10	4M; 6F	Bangalore	2	5 h

###### 2.3.6.1.2 Procedure: co-production workshops

A total of 10 workshops were delivered by the NIMHANS team between November–December 2021, each lasting between 3 and 5 h. Separate workshops were conducted for each stakeholder group (adolescents, educators, parents, mental health professionals). Workshops with adolescents and parents were in school and those with teachers and head teachers were at NIMHANS. All workshops were conducted by facilitators proficient in Kannada, except for those with mental health professionals which were conducted in English. Informed by our adaptation hypotheses, we prepared semi-manualised workshop guides to support each stakeholder group's exploration of each prototype intervention and its anticipated outcomes, implementation, associated ethics/safeguarding, methods of intervention evaluation and preferred vocabularies. Mindful of the potential varied literacy levels among participants, the workshop materials and activities avoided technical concepts or jargon, were based on talk, art or movement (i.e., no reading or writing required) and were finalized by the Indian team and YAB to ensure they would be likely to make sense to local participants. Workshops began with project introduction and our scope for co-adaptation and co-production. Typical co-production activities were used (86, 87), such as creating personas and considering vignettes, with differing modes of contribution (e.g., stepping toward or away from statements to indicate agreement; raising hands to vote; assigning stickers to rate preferences; small group discussions). Gender was also explored in terms of preferences for tailoring program content or activities by gender, possible unequal program effects by gender, and cultural sensitivity (See Supplementary File 3 for examples of workshop activities).

###### 2.3.6.1.3 Data collection

Participants were highly engaged and expressed appreciation at the opportunity to contribute to school mental health programs in this way. Workshops were digitally recorded, transcribed verbatim (removing identifying information) and then translated into English with 5% back translation accuracy checks. Workshop artifacts (posters, rankings etc.) were photographed, scanned and uploaded. Workshop facilitators field notes supported data analysis.

###### 2.3.6.1.4 Data analysis

Analysis was conducted by an Indian and UK sub-group of the SAMA team involving senior researchers and research assistants, with oversight from an independent steering group and advisory group. Analysis was organized by stakeholder group and then by workshop discussion point. Relevant transcript extracts, workshop artifacts and facilitator notes were linked to these. This data corpus was then subjected to a form of framework analysis (88). We utilized a codebook approach to organize and deductively code data as being relevant to: the aims or components of a particular intervention; implementation; ethics/safeguarding; gender issues; or research evaluation or language (i.e., culturally appropriate vocabularies). As per framework analysis, we also coded inductively to capture insights not in these a priori codes. We then conducted a form of indexing and charting. This involved re-organizing the coded data from the different stakeholder groups around each intervention/issue (e.g., all stakeholders' views on implementation).

#### 2.3.7 ADAPT step 7: undertake modifications

Following Moore et al.'s (67) decision tree for adapting vs. creating new interventions, the UK and India team collaboratively adapted the prototypes to improve intervention-context fit and created new components or content where needed. New components were developed based on Indian or international evidence where possible, which we report below. We also generated a final safeguarding protocol (described in detail elsewhere; forthcoming) and refined our evaluation protocol. We brought all resources for review by the wider SAMA team, the oversight groups and the YAB to solicit their assessment of whether these were contextually and culturally appropriate, feasible and safe. After modifications, a final review was conducted by three to four independent local mental health experts per intervention, who were asked to rate the clarity, relevance, cultural appropriateness, acceptability, and ethicality of the intervention along with overall feedback.

## 3 Results

We present our findings for each stage of ADAPT and key outcomes from each step are summarized in Supplementary File 4.

### 3.1 ADAPT step 1: initial assessment

Supplementary File 5 shows the outcomes of our multi-level needs assessments. We identified that any intervention put forward for co-adaptation must: be compatible with Indian values and perspectives; be suitable for 15-year-old adolescents in Grade 10, as reaching older students would be difficult given exam years; be sensitive to stigma associated with mental health conditions; be experiential and without high literacy demands. In addition, as reported by Parikh et al. (26), the interventions must address adolescents' stress from multiple sources, speak differently to youth, teachers and parents, include psychoeducation and be solution-focused, especially to cope with interpersonal problems.

### 3.2 ADAPT step 2: intervention selection

We conducted four reviews to identify candidate interventions to take forward to co-adaptation. Supplementary File 6 shows the Prisma flow chart for each review, the returned reviews and how we arrived at a final pool of eligible interventions.

- **Umbrella review of whole school programs to reduce adolescent anxiety and/or depression**. A total of 523 unique systematic reviews were retrieved. Following review, eight interventions (one targeting anxiety and depression, two targeting anxiety and five targeting depression) were eligible for progression to ADAPT Step 3. Manual searches identified two further eligible intervention.
- **Umbrella review of interventions to improve secondary school teachers' mental health literacy**. Three unique systematic reviews were retrieved, which led to the identification of four unique interventions targeting teacher mental health literacy. Manual searches did not identify any further eligible interventions.
- **Umbrella review of interventions to improve school climate in secondary schools**. Five systematic reviews were retrieved, but no eligible interventions were identified. Manual searches returned two interventions eligible for progression to ADAPT Step 3.
- **Umbrella review of interventions to improve parents' mental health literacy**. One systematic review was retrieved, and three eligible interventions were identified. Manual searches returned one further intervention.

### 3.3 ADAPT step 3 and 4: intervention exploration and potential mismatches

Each candidate intervention was explored by first completing a template to support team discussions (Supplementary File 7). From the 10 candidate whole school programs targeting adolescent anxiety and/or depression, all except two [SEHER (59) and Shamiri (88)] were either not available/accessible, did not met our inclusion criteria and/or did not have any evidence of sustained effects. From the three candidate interventions for teacher mental health literacy, only one was later deemed eligible to take forward to co-adaptation [Canadian Go-To-Educator Training Curriculum (89, 90)]. For school climate, only one intervention was eligible to take forward to co-adaptation (already identified SEHER (59). For parental mental health literacy, the two candidate interventions were not available or accessible so we drew from the resources returned from the desk review (explained in ADAPT Step 5). From each eligible intervention, we identified “potential mismatches” (reported below) to focus on in Step 5, defined as intervention components or approaches with high or uncertain acceptability and feasibility in the local setting and therefore requiring adaptation.

### 3.4 ADAPT step 5: intervention model development

**Figure 2:**
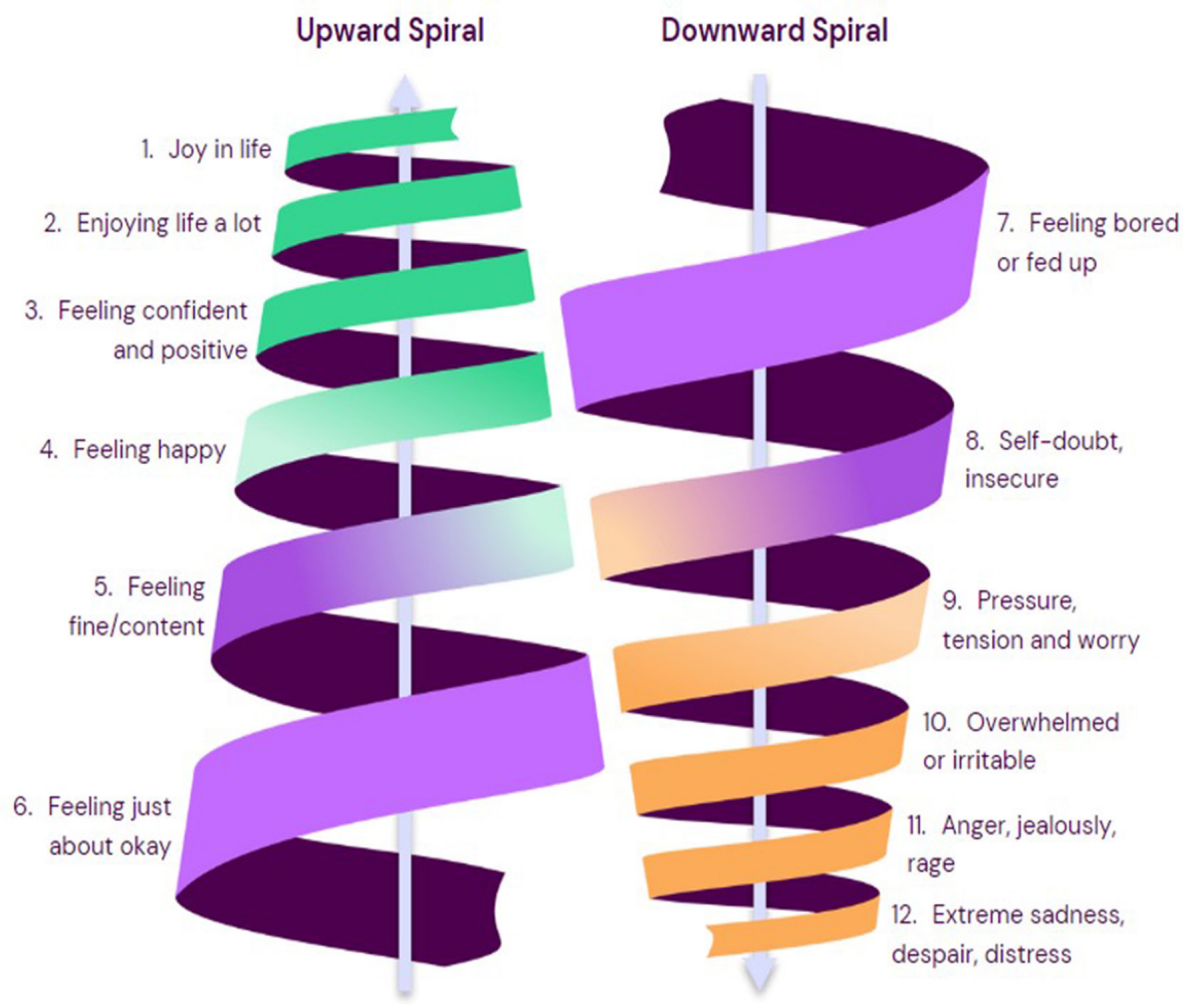
SAMA spiral concept to support emotional awareness.

To take to co-production, we created prototypes for four interventions, anticipated to function synergistically as a whole school program for adolescent mental health (Table 2). We scrutinized the SEHER and SHAMIRI manual for content and approaches that could be used in Intervention 1 prototype (SAMA for Youth). The extracted content is reported in Supplementary File 7. Given the lack of available intervention manuals from the umbrella review, we drew on additional evidence of common components of effective adolescent school mental health programs, specifically Clarke et al. (91); Gimba et al. (92), Skeen et al. (93) and WHO's Helping Adolescents Thrive Toolkit (94), as well as the Wellcome Trust's report of active ingredients for prevention and early intervention in youth anxiety and depression (95, 96). Supplementary File 8 shows our synthesis of common effective mechanisms across these reviews and how these mapped to our final intervention 1 prototype. The stated aim for prototype intervention was to help adolescents build practical skills for wellbeing and for managing early symptoms of anxiety and depression via an eight-week classroom-based psychoeducation program. This was designed to be delivered to standard class size (*n* = 30–40) in 9th grade (14–15 year) by a lay counselor embedded in the school. It utilized a concept of a “SAMA Emotion Spiral” (Figure 2) reflecting normal changes in mood, upwards and downwards, and that awareness of “how we are” can help us respond helpfully.

**Table 2:** Overview of SAMA prototype whole school program taken to Step 6.

**Program intervention component**	**Prototype intervention structure**	**Prototype intervention content**	**Proposed implementation**
SAMA for Youth	8 × weekly 60-min classroom-based sessions with student booklet plus 1 × booster	*Promotion* (growth mindset, setting goals, wellbeing strategies, living with values); *Prevention* (what challenges our wellbeing; mental health literacy, emotion regulation, relationships, problem-solving, CBT for anxiety and depression; *Redressal* (help-seeking).	Lay counselors
SAMA for Teachers	7 × 90-min sessions every 2–3 weeks	Teacher value and stressors; mental health literacy (incl. stigma); promoting student wellbeing, help-seeking and resilience; forms of discipline/teaching practices and impacts.	Mental Health Professionals and Lay counselors
SAMA for School Climate	6-month portfolio of activities	Whole school and classroom level (as per SEHER); Schools network to support shared learning across SAMA; whole school policies on bullying and student wellbeing	Mental Health Professionals
SAMA for Parents	A day workshop	Understanding changes in adolescence; mental health literacy; communication and support.	Mental Health Professionals

The aim of intervention 2 prototype (SAMA for Teachers) was to increase teachers' mental health literacy and teachers' use of positive classroom practices. This was created by first drawing upon the Canadian Go-To-Educator Training (89). Supplementary File 8 details the content mapping. We identified only a few mismatches. For example, the source intervention largely conceptualized mental health stigma as an individual attitude. Adaptation was needed to acknowledge the cultural and religious views and beliefs which shape Indian perspectives on the origins and nature of mental health (97). We drew upon other Indian manuals/resources, namely the White Swan Foundation's “Youth Mental Health: Teacher as Catalysts” (98) and Fortis' Mental Health in Classroom Curriculum (99) for culturally relevant examples and ways of talking about mental health literacy (e.g., concept of burden vs. stress, talking about “challenges” rather than “problems”). For intervention content to promote teachers' use of positive practices in the classroom, our manual searches identified the Positive Discipline and Classroom Management by the Center for Justice and Crime Prevention and the Department of Basic Education, Pretoria (100). Supplementary File 8 shows the content taken from this. Some mismatches were identified. For example, the Indian team felt more focus was needed to explore how teachers were affected by the expectations held by some Indian parents that schooling will administer tough (including corporal) discipline when needed. The final intervention 2 prototype proposed 7 × 90-min sessions delivered over 2–3-weeks (see Supplementary File 8).

To develop intervention 3 prototype (SAMA with Schools), we drew upon SEHER's school climate intervention (59) but made several modifications. For example, we dropped the development of school policy on substance use but added one on school wellbeing, and we changed the focus of classroom workshops to mental health related issues (e.g., we dropped study skills content). We also drew minimally upon two further programs -the Gatehouse Project (101) and School Climate Improvement Resource Package (102). However, most content from these programs constituted mismatches either to our aims or resources (e.g., developing in-school monitoring and evaluation teams). Supplementary File 8 details how the content from each of these fed into our final prototype SAMA with Schools. This multi-component intervention was proposed to be delivered by a lay counselor over 6 months.

For intervention prototype 4 (SAMA with Parents), we explored the Early Adolescent Skills for Emotions (EASE; psychoeducation parent sessions) (103) and the Go-To-Educators Training (104), especially on addressing mental health stigma. However, much of the content of these programs was not aligned with our aims to improve parents' mental health literacy or were not compatible with the short duration that we were going to be able to engage parents. Some mismatches could be addressed. For example, the EASE program content on parental self-care was replaced with a short focus on self-management in times of high stress with their child. A substantial proportion of the intervention content was created from the significant professional and local knowledge of the NIMHANS team. The final prototype intervention (see Supplementary File 8) was proposed to be delivered as a half day workshop on school premises. The stated aim was to improve parental mental health literacy as a pathway to support adolescents.

### 3.5 ADAPT step 6: co-adaptation/co-production

This section details the key outcomes from co-production workshops with young people, teachers, parents, and mental health professions for each of the interventions. All stakeholders strongly endorsed a whole school approach with the four interrelated interventions and that Grade 9 adolescents were a suitable age group for a feasibility trial as they were likely to be able to benefit from a psychoeducation intervention at a good pre-exam juncture. School staff indicated acceptability would be strongly linked to the awareness of the program across the school community and how well its' aims and anticipated benefits were understood.

#### 3.5.1 SAMA for Youth

All stakeholders felt that the intervention's success would be influenced by its age- and cultural-relevance. None felt that it risked escalating mental health stigma. Only one teacher was concerned that the intervention would take time from curriculum teaching. For intervention accessibility and inclusivity, teachers requested it was available for students in English and Kannada.

Certain intervention concepts and vocabularies were preferred over others (Supplementary File 9). Adolescents and teachers rejected the term “resilience” and proposed “overcoming difficult situations” instead. “Mental health” and “mental illness” were acceptable to all except the mental health professionals who preferred “wellbeing” as a less stigmatizing term. Adolescent boys suggested that “managing difficult feelings” be explained as “defense mechanisms” (a term in India referring to protection from difficult feelings), and all adolescents were unsure about the meaning of “coping.” The word “stigma” appeared hard for all to understand, even when translated into the local language (as they associated it with the plant anatomy “stigma”). During workshops, it also became clear that adolescents did not fully understand the concept of “self-esteem.”

The intervention prototype content was endorsed and deemed necessary, but insufficient, by all other stakeholders, with varying views of priority content. This is shown in Table 3 where we distilled prototype content into key issues. Parents, teachers and mental health professionals requested additional coverage of technology addiction, living by values, sustaining focus on academic achievement and family issues. All adolescents said they would be uncomfortable discussing “when things are difficult at home” as this was a private matter for their family. Mental health professionals offered suggestions about addressing teacher-student bullying and some content for the psychology-focused sessions. They emphasized that values in Indian culture are highly personal and that adolescents should be offered a chance to articulate their own values at the start of the intervention. Adolescent boys requested content on improving self-confidence, defense mechanisms, practical ways to cope with stress, ways to understand and reach your goals, and top tips on how not to be anxious or disappointed. Clear, in-school, help-seeking pathways were also important to adolescent boys. Adolescent girls emphasized the need for sessions on how to distinguish between “good and bad” friends and how to maintain good friendships. Neither adolescent boys nor girls rated self-compassion as a necessary topic. Stakeholders broadly offered the same reasons for endorsing the prototype content which centered around the empowering of adolescents to understand and manage their own wellbeing by up-skilling them with mental health literacy, problem solving abilities and knowledge of help-seeking options. All stakeholders, including parents, stated that they felt SAMA for Youth was important and should be part of the school curriculum.

**Table 3:** Stakeholder views on topics to be covered in the SAMA for Youth intervention.

**Topic**	**Stakeholder group**				
	**AB**	**AG**	**P**	**T/HT**	**MHP**
Learning top tips for feeling good	•	•	•	^*^	•
Knowing about good physical health (sleep, food, and exercise)	•	•	•	^*^	•
Learning ways to manage stressful times (e.g., exams, big decisions, big changes)	•	•	•	^*^	•
Knowing how to have better relationships with friends	°	°	•	^*^	•
General coping skills for hard times	°	•	•	•	•
Knowing how to do better in academics	▪	•	•	•	•
Learning ways to manage feeling sad, down flat or upset	•	•	•	•	•
Knowing how to handle difficult situations (arguments, peer pressure, bullying)	°	•	•	•	•
Gaining more self-confidence	•	•	•	•	•
Learning relaxation skills	▪	•	•	•	•
Knowing about how substances might affect me (as an adolescent)	▪	•	•	^*^	•
Knowing what options I have if I need help	•	•	•	•	•
Improving self-compassions and kindness to others	▪	▪	•	•	•

AB, adolescent boys; AG, adolescent girls; P, parents; T/HT, teachers/head teachers; MHP, Mental health professionals. • = a session on this would be really useful; ° = a session on this might be useful; ▪ = no need; ^*^ priority topics for head teachers.

Most stakeholders endorsed highly visual mini booklets to support youth self-reflection and content consolidation. Teachers and head teachers suggested vignettes as a cultural and age-appropriate discussion tools that minimize personal disclosures and vulnerability. Both adolescent girls and boys wanted sessions to be mixed-gender, different to typical classes, informal and highly interactive with many opportunities for peer-to-peer engagement, including role playing, group discussions, problem solving tasks, discussing example scenarios, peer-to-peer teaching as well as time for setting personal goals and personal reflection. However, adolescents deemed some issues as unsuitable for peer discussions. Some boys worried about discussing boy/girl relationships and some girls said they would feel uncomfortable sharing personal views on “what helps students feel calm and happy.”

Optimal intervention implementation was collectively endorsed as one, 1-h session per week for 8 weeks plus one booster session between 3 and 6 months later. Head teachers were willing to approve two 1-h sessions per week as they felt some topics would need more time to be covered in appropriate depth. Delivering some sessions outside classrooms (e.g., communal spaces) was suggested by head teachers to facilitate a more informal context.

All stakeholders were united in a preference for lay counselors to deliver this intervention, with provisos. For teachers and headteachers, provisos included having a clear understanding of how lay counselors had been trained, would integrate into the school community, would collaborate with teaching staff, and how they would be occupied in school when not delivering the intervention. Headteachers stressed the importance of the lay counselors understanding and aligning with the unique culture of their school to aid intervention implementation. Head teachers felt it was important that lay counsellors' interactions with students would be appropriate (including no male counselors meeting with female students) and in line with the school ethos (friendly vs. formal interactions), that they were educated on all school procedures for safeguarding and disclosures and that students would be clear on their role compared to typical teaching staff. Attention to a manageable class size for them as also important (typically 60 students). Mental health professionals stressed the importance of regular supervision for lay counselors, both individually and as part of a group, to facilitate shared learning, connection and support.

Adolescents emphasized that lay counselors were responsible for creating relaxed, fun and inclusive intervention sessions, driven largely, they felt, by the counsellor's personal orientation to them. They stressed that lay counselors needed to reassure them about how safe it will be to be open in SAMA discussions or the accompanying booklets, and that it was crucial that no information would be shared with others (e.g., via-a-vis safeguarding) in ways that disempowered them (e.g., they wanted to play an active role in identifying their trusted person).

Finally, with regards to research methodology to evaluate this intervention, it was important to parents that consent information be sent in a local language. All stakeholders agreed with the following statements: “parents should not be able to stop their son/daughter learning about their emotional wellbeing”; “schools, teachers, parents, and students have a right to see the data from the project”; “permission should be secured from students for data collection” and “a student can say they do not want to take part even if the rest of the class does.” Adolescent preferences for outcomes included changes in their feelings and thoughts about themselves and their lives and they felt that their parents should have access to school level, not individual level, data yielded from any validated measures they completed. Teachers and adolescents both emphasized the importance of confidentiality in data collection and to counter cultural perceptions by emphasizing to adolescents that measure completion was not an exam.

#### 3.5.2 SAMA for Teachers

All stakeholders endorsed the aims of the “SAMA for Teachers” prototype intervention and stressed headteacher buy in would be pivotal. They felt it should be for all teachers (not selected ones) with time made available in their schedules and teachers recognized for their take-up of training. Psychoeducation on mental health literacy was welcomed, including the role they as teachers could play in spotting early signs of poor mental health in students. They recommended additional topics (time management and anger management for themselves), making the module on resilience building mandatory (although they preferred the term “overcoming difficulties”), optimizing impact and engagement via peer discussion, implementation in 1.5/2 h blocks every 2–3 weeks over June-August with booster sessions every 3 months, and overall intervention integration into a comprehensive school health program. They suggested bringing teachers from different schools together so that there is “seriousness” during the sessions. Partnering with other intervention schools to support engagement and shared learning was also recommended. Participants welcomed training on positive classroom practices, although headteachers recommended delivery by a professional figure, not lay counselors, as they anticipated it would be hard for senior teachers to let go of tough disciplining methods. MHPs recommended the intervention should cover developmental perspectives, the bio-psycho-social model of mental health, sensitivity to LGBTQ+ issues, contemporary issues for adolescents and using IEC materials on social media to help sustain the program. Young people endorsed the teacher training as presented in the prototype.

#### 3.5.3 SAMA for school climate

We defined school environment as in SEHER, namely the pattern of students', parents', and school personnel's experience of school life [that] reflects norms, goals, values, interpersonal relationships, teaching and learning practices, and organizational structures (59). All stakeholders understood and accepted the importance of school climate as we defined it (although they preferred the term “school environment”) and endorsed the prototype's aim, content and implementation strategy. All adolescents said they would welcome the opportunity to be proactive in school via this kind of intervention. Only adolescent girls suggested that the intervention especially emphasize school (relational) connectedness, which they described as: support from friends, a helping nature in everyone, sharing difficulties with others and absence of any kind of discrimination. Adolescents wanted to expand the role of the peer leader, from supporting the lay counselor to also offering support to fellow students. MHPs and school staff emphasized that intervention success would be contingent upon initial and continued understanding and awareness of the intervention by everyone in school, with headteachers suggesting intense activity was needed, spanning media coverage, social media and easy communication channels such as WhatsApp. Teachers stressed the importance of letting young people decide which adults should join the intervention committee, that teachers play a supporting rather than leading role, and that non-stigmatizing language be consistently employed to avoid students or teachers labeling each other. Headteachers underscored that the lay counsellor's role and responsibilities be made clear to parents and staff, including communication and referral plans. MHPs also stressed lay counselor training and protocols should address student anonymity and confidentiality and decision-making for referrals. Headteachers suggested omitting initial data collection to inform school environment priorities as the cultural norm would be to collectively decide these through discussion, and to incentivise student engagement with inter-school competitions. Interest in a network for SAMA schools to collaborate and share ideas and learning was only moderately supported as this is not a typical cultural practice.

With regards to evaluating this intervention component, given adolescent literacy levels and preference for Kannada, teachers suggested data collection from adolescents be as simple as possible (including rating scales, and to avoid the format of questions and sub-questions). Adolescent boys suggested individual or group discussions as a good data collection method.

#### 3.5.4 SAMA for parents

All stakeholders endorsed the prototype aim, content and implementation, and would welcome the intervention into schools. Adolescents conveyed their challenges with parents to which the intervention should be sensitized, which included: corporal punishment, comparison with other children, autocratic parenting, poor knowledge about adolescence and perceiving adolescents to be mischievous, indulging in fights and keeping bad company. They conveyed that an intervention for parents might convey adolescent needs which they stated were: to be accepted by parents, to have freedom, to have quality time with parents, to involve them in decision-making rather than dictating, to be understood and believed, to be supported, guided and encouraged, not to be blamed or punished, to be taught moral values to have help during difficult times, and to not be too lenient or too strict.

In discussing potential barriers to intervention success, MHPs emphasized low education and low literacy levels of our target demographic and conveyed their view that most Indian parents will not have discussed mental health or been involved in such an intervention before. All stakeholders emphasized the need for accessible materials (e.g., brief videos, highly visual booklets, local languages). Local variance in terms of caste and religion, and associated differences in parental practices and preferences, were additional anticipated challenges requiring sensitive intervention content. Parents said they welcomed the opportunity to learn about parenting their adolescent via this proposed intervention but saw potential attendance barriers because of poor understanding of the problem and other pressing issues like financial constraints, work pressure and family problems. Many parents in the region work on daily wage basis (below the poverty line) and time from work for intervention attendance was considered a major barrier. An additional anticipated barrier to securing positive intervention outcomes was the lack of opportunity for parents to act on intervention guidance and to spend quality time with their children.

### 3.6 ADAPT step 7: undertake modifications (finalize interventions)

Step 6 confirmed acceptability of the intended aim of the SAMA program to reduce symptoms of anxiety and depression in adolescents. Informed by Step 6 outcomes, Step 7 involved modifying the prototypes to generate four final interventions that would constitute our SAMA whole school program, ready for feasibility testing. The content for these finalized interventions, which we detail below, was favorably reviewed by our independent local mental health experts (see Supplementary File 9). We planned to use the Reporting Adaptations and Modifications-Enhanced (FRAME) (77) to document how we had adapted candidate interventions from Step 2 prior to pre-feasibility testing. However, this was not meaningful as none of our final prototypes were based on any single existing program. Supplementary File 10 details our TIDieR checklist (105) for the whole school program and our Type 2 logic models for each intervention and likely moderating factors. Although Type 1 and 2 logic models are limited in their suitability for complex interventions (106), they are a useful organizing framework at a feasibility stage. Our logic models show the key intervention content and the hypothesized mechanisms of change leading to proximal outcomes. They also show the “Inner Context” moderators which we envisage may affect the change process.

### 3.7 Final implementation protocol

We finalized safeguarding, implementation and evaluation protocols following stakeholder input in Step 6 (available at www.sama.org.uk). The school communities suggested the term “wellness coach” for the lay counselor and their chosen name is “SAMA Snehitharu,” meaning friend. We have created an in-person training course for lay counselors (to be delivered by NIMHANS) as well as intervention training manuals and safeguarding protocols [in English and Kannada (available at www.sama.org.uk].

Simultaneous delivery of our four interventions as a whole school approach was endorsed in the co-design process, with only minor modifications. Responding to the Step 6 findings on the importance of whole school awareness and understanding the programme, our final implementation protocol is to: (i) launch “SAMA is coming” poster campaign in schools 1 month pre-delivery; (ii) integrate lay counselors into schools; (iii) deliver whole school assemblies explaining the programme and anticipated benefits; (iv) SAMA for Youth (delivered by lay counselors) broadly simultaneously with SAMA for Teachers (delivered by NIMHANS); (v) SAMA for Schools launch 1 month later (delivered by lay counselors); (vi) SAMA for Parents approximately 2 months later (delivered by NIMHANS).

#### 3.7.1 Final intervention: SAMA for Youth

**Figure 3:**
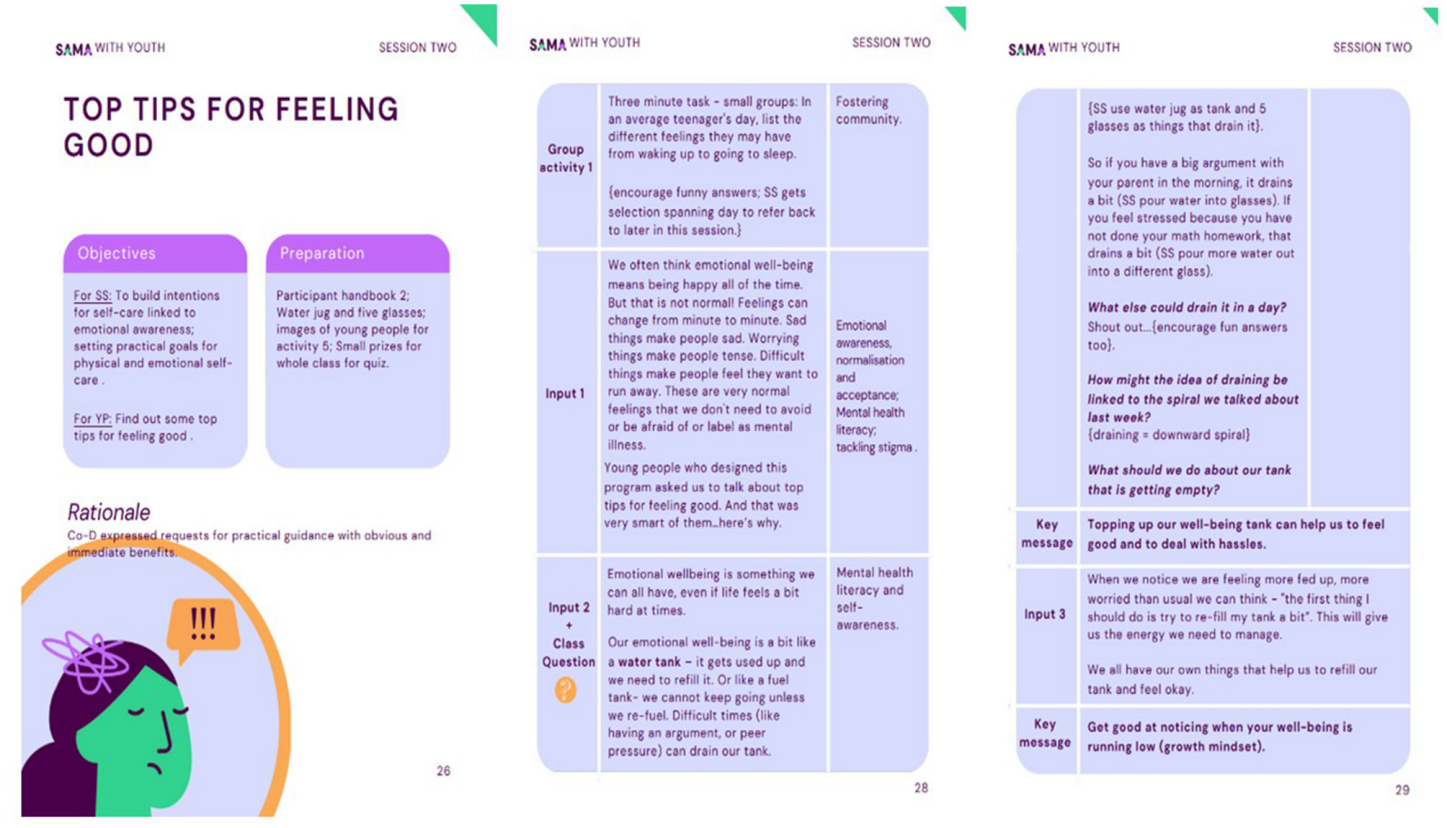
Example lesson for SAMA for Youth manual.

The primary aim of this intervention ready for feasibility test is to reduce adolescent symptoms of anxiety and depression, via improved mental health literacy and psychoeducation, school connectedness and supportive relationships. It has now been manualised for delivery to Grade 9 students (14–15y) via 8 × 40-min, evidence-based, topic-led sessions for delivery to a whole class (30–40 students). Each session has an accompanying student booklet for personal notes and a reminder of key learning. Based on the eight topic sessions, 13 extension sessions were also created for use if lay counselors were asked to cover for absent teachers. Topics move from wellbeing promotion to prevention of poor mental health and redressal if symptomatic. All sessions follow SAFE principles [sequenced, active, focused and explicit; (91)], are similar in structure and prioritize: fun, student interaction; student voice and assets; being different to everyday lessons; use of vignettes, role-play and creativity; and practical application in everyday life. The intervention contains many of the evidence-based active ingredients for prevention and early intervention in youth depression and anxiety documented by the Wellcome Trust's reviews (95, 96), including behavioral activation, problem-solving, relaxation techniques, emotional awareness and regulation, decentering, addressing repetitive negative thinking, improving social relationships and mental health literacy. Figure 3 shows manual examples, Table 4 shows the final intervention content and Supplementary File 10 shows the intervention logic model and final agreed outcome to be measured.

**Table 4:** Final version of SAMA for Youth intervention ready for feasibility testing.

**Session number**	**Prototype version**	**Final version (after co-production)**	**Objectives for adolescents**	**Content/ Objectives/ Rationale**	**Common across all sessions**
1	**Setting intentions**	**Getting started** Extension Lessons: Exploring emotion spirals	To find out about the SAMA program and get to know your SAMA Snehitharu	Establish SAMA as different to normal classes; build understanding and motivation for program benefits; introduce wellbeing. SAMA spiral/emotional awareness; growth	1 × minute mindfulness at session start and end (a minute for you) Building relationships between lay counselor and peers Connection to SAMA Spiral and Growth Mindset Focus on personal strengths and practical and memorable strategies
2	**Protecting wellbeing**	**Top tips for feeling good** Extension Lessons: Strategies for wellbeing	To find out some top tips for feeling good	Things that drain and boost our wellbeing (self-awareness); build permission, intention and practical strategies for self-care; goal setting with IF-THEN plans	
3	**Living with values** *(Dropped following co-production)*	**How to manage pressure at school** Extension Lessons: IF-THEN plans; Relaxation	To have top tips for managing school pressure	Good vs. bad stress; spotting signs of stress; MHL, resource-demand model of stress; self-efficacy for coping; practical coping skills with IF-THEN plans	
4	**Challenges to wellbeing**	**Dealing with problems (when things are bothering you)** Extension Lesson: Problem-solving; Playful Acceptance and Traffic Light Approach	To learn how to use a problem-solving approach when things are bothering you	MHL (normalization of everyday worry); anxiety/worry as problems; options for approach problems (problem-solving, acceptance; developing problem-solving as a coping skill	
5	**Dealing with difficult feelings**	**Managing difficult feelings (low mood)** Extension Lessons: Ignore the Passengers	To understand low mood and what you can do so that you can carry on doing the things you enjoy	MHL (normalization of varying mood); CBT model for persistent low mood; behavioral activation; IF-THEN plans	
6	**Dealing with difficult thoughts**	**Building positive relationships** Extension Lesson: Design a welcoming school	Top tips for good relationships	Celebrating and accepting difference; empathy for others; motivation for positive relationships.	
7	**Relationships**	**Building positive relationships** Extension lesson: managing tricky situations; creating a safe school	Top tips for friendships	Characteristics of good friendships; spotting bullying and zero tolerance approach; safeguarding	
8	**Being Supported**	**Putting it all together**	To celebrate all you have achieved on SAMA	Recognition of strengths; identifying personal strategies to continue; routes to support in school	

#### 3.7.2 Final intervention: SAMA for Teachers

It was agreed that the aim of this intervention would be to improve teachers' mental health literacy and teachers' use of positive discipline practices. No content changes were requested but participants provided suggestions to making it easier for teachers to learn and implement strategies. Planned delivery is seven short modules (manualised), by NIMHANS mental health experts, over 2 days to all teachers in SAMA study schools, with delivery to each school in isolation. We plan high levels of peer discussions based on scenarios/vignettes and some role-play. From suggestions in Step 6, we opted not to bring study schools together for this intervention delivery given challenges to coordination, omit topics recommended by MHPs which were beyond a focus on mental health and not to use social media to sustain the program as this was beyond the scope of the project. Table 5 shows the final planned intervention and Supplementary File 10 shows the intervention logic model and final agreed outcome measures.

**Table 5:** Final version of SAMA for Teachers intervention ready for feasibility testing.

**Session number**	**Final version (after co-production)**	**Objectives**
1	**Teacher Wellbeing**	Validate the importance of teaching, teacher wellbeing and teacher-student relationships
2	**Understan ding mental health and mental health conditions**	Improve knowledge mental health, signs of mental health conditions and support/ treatment available.
3	**Destigmatising mental health conditions**	Increase understanding of: mental health stigma; its manifestations (facts, factors and myths); and evidence-based strategies to reduce stigma in schools
4	**Encouraging help-seeking**	Improve understanding of the importance of help as early intervention; types of, and ways to, help; barriers to help seeking
5	**Mental health promotion and wellbeing activities**	Improve knowledge of the importance of mental health promotion; practices to promote wellbeing and stress management? in teachers and students
6	**Resilience** (Overcoming Difficulties)	Increase knowledge of the meaning of resilience and how to foster it in teaching practices
7	**Positive discipline and classroom management**	To promote: knowledge of harms of harsh discipline; forms and benefits for self and students of positive practices; how to implement in classrooms and school policy

#### 3.7.3 Final intervention: SAMA for Schools

The agreed aim of the school environment intervention is to promote the psychological safety of students in school, enhance their school connectedness and promote mental health literacy. The final intervention is multicomponent spanning inter-school, whole school and class level elements. From suggestions in Step 6, the final intervention includes proactively encouraging girls to join the school environment team, improved protocol clarity in relation to managing the Speak Out Box (e.g., if students lodge complaints about staff), expanding the role of peer leaders with skills training, and incentivizing the school competitions (see Table 6). Supplementary File 10 shows the intervention logic model and final agreed outcome measures for this intervention.

**Table 6:** Final version of SAMA for School intervention ready for feasibility testing.

**Whole school level**	**Session number**	**Prototype version**	**Final version (changes following co-production)**	**Objectives**
**Class level**	0	Data collection to inform intervention focus	Omit due to preference to decide via discussion	
	1	SAMA School Network	Unchanged	To support intra-school sharing of ideas and learning; to support sustainable practice
	2	Awareness Generation	To also begin in advance of program launch	To generate awareness of school wellbeing and engagement with SAMA, including parents
	3	School Environment Team	To also actively promote inclusion of girls to the team	To support new school processes for student-teacher collaboration; to promote youth voice; to build students' confidence as change agents and enhancing school connectedness?
	4	Speak Out Box	Clearer protocols for managing submissions	To promote perceived support and help-seeking
	5	Wall Magazine	Unchanged	To support student-led engagement on topics related to their wellbeing (improve mental health literacy); to tackle mental health stigma
	5	Anti-bullying and Wellbeing Policies	Unchanged	To support student-school collaboration on designing policies and processes to address school risk and protective factors for adolescent mental health
	6	Inter-school Competitions	To also integrate with existing school processes for competition.	To support student-led engagement on topics related to their wellbeing (improve mental health literacy); to tackle mental health stigma
	7	Lectures and Workshops	Retain student workshops only to not replicate SAMA for Teachers content	To support mental health literacy; foster positive student relationship and school connectedness
8	Peer Groups	Expanded role for the peer leader to include training to enhance listening and responding	To promote peer-to-peer support; to foster positive student relationships; to promote perceived support

#### 3.7.4 Final intervention: SAMA for Parents

Our study protocol (63) specified our intention to explore the feasibility of reaching parents. Following our ADAPT steps, we were able to go beyond this and establish a parent intervention. The endorsed primary aim of the created intervention is to promote parents' mental health literacy, and we incorporated most recommendations from Step 6. Planned delivery is by NIMHANS mental health experts, in a local language, and over one-half day workshop (informed by the stated likelihood of parents' capacity to attend given work and family commitments). Two, 1-min videos were created to support understanding of the symptoms of anxiety and depression, to be distributed via WhatsApp. Table 7 shows the final intervention content and Supplementary File 10 shows the intervention logic model and measures.

**Table 7:** Final version of SAMA for Parents intervention ready for feasibility testing.

**Session number**	**Prototype version**	**Final version (after co-production)**	**Objectives**
1	Introduction to project	About SAMA	To introduce the SAMA project, its aim, objectives and build rapport
2	Adolescent mental health	Exploring parental understanding of adolescent mental health	To identify the gaps in knowledge, beliefs and attitude
3	Parent child relationship and expectations	Understanding your child and parent expectations	To explore the knowledge and attitude of parents toward adolescent children
4	Addressing stigma	Adolescent mental health and addressing stigma	To reduce stigma and improve help-seeking behavior
5	Prevention of mental health issues and improving wellbeing	Mental health promotion and wellbeing activities	To empower parents with skills and attitude to promote emotional wellbeing
6	Adolescent mental health problems	Identification of mental health conditions in adolescence	To empower parents to identify early warning signs and symptoms of mental health problems
7	Strategies to improve adolescent mental health	Tips for managing adolescent mental health	To equip parents with actionable tips and resources to support adolescent children in improving mental health
8	Debriefing or summarizing	Reflect and review	To reflect on learning and gather feedback

#### 3.7.5 Program theory of change

Schools are “social complex adaptive systems” which respond unpredictably to interventions (1107). We therefore characterize SAMA as a complex intervention and anticipate adaptation and learning during program delivery, including the re-prioritization or changing of activities and within-system innovation (i.e. prioritization of adaptation over fidelity). This is especially important given the general lack of evidence over successful implementation approaches for school-based programs in India (107).

Acknowledging this, at this point we theorize that the interventions will work synergistically as a whole school approach to reduce the prevalence of adolescent anxiety and depression. Our logic models reflect our anticipation that the interventions will reduce the symptoms of anxiety and depression in Indian adolescents by reducing risk factors and promoting protective factors. Risk factors for adolescent anxiety and depression targeted in the interventions include: (i) low mental health literacy among students, teacher and parents, (ii) mental health stigma (which can lead to concealment of early symptoms and need for support), (iii) bullying, and (iv) teacher punitive practices (including corporal punishment). The interventions are also hypothesized to target the following promotive factors which can protect against adolescent anxiety and depression: (i) youth mental health literacy, psychoeducation and wellbeing education and strategies, (ii) positive student-teacher relationships (supported by positive classroom practices), (iii) teacher and parent mental health literacy, (iv) school climate (including psychological safety and belonging), (v) normalization and ease of support-seeking, and (vi) parent-child communication. “Inner Context” moderators that we envisage may affect the success of the program include school leadership support (e.g., allocating time and space for intervention delivery), the effectiveness of the lay counselor (influenced by training, support and school acceptance); student, teacher, and parent motivation and capacity to engage (influenced by co-occurring pressures and demands); and school culture on youth wellbeing, voice and youth-led change.

**Figure 4:**
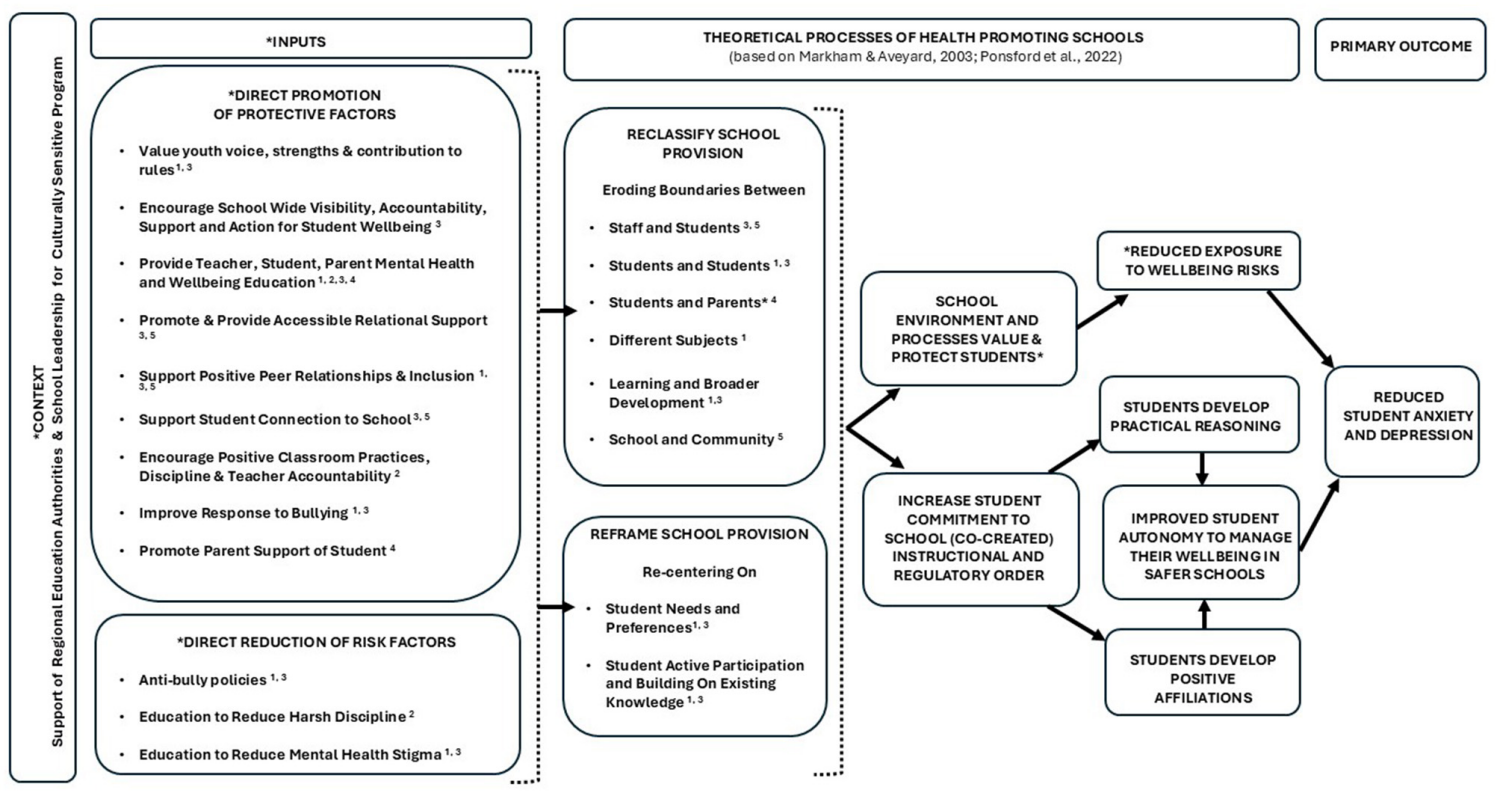
Our theory of change for the whole SAMA program based on the theory of health-promoting schools.

However, we propose moving beyond purely theorizing the dynamic interplay between risk and protective factors for youth mental health which can risk oversimplifying complex interventions. We therefore developed a Theory of Change (ToC) (Figure 4) for our prototype whole school program. This is based on an adaptation of Markham and Aveyard's (61) health-promoting schools theory given its prima facie resonance with our prototype interventions, It draws on cultural and educational theories which posit that students are aware of the dominant approach to education, internalize the school's expectations and value these in the same way the school does (108). A central tenet of Markham and Aveyard's (61) theory is that student health is promoted when the whole school experience (rather than bolt on activities) enables and motivates students to autonomously choose to function well and flourish. This is more likely when schools fulfill two primary human needs for health: the ability to imagine, think and reason (i.e., to act on education and knowledge), and the ability to have affiliation and mutually satisfying relationships with others. Schools can fulfill these needs via their organizational systems and interactions within those systems, drawing on concepts of instructional order, regulatory order and eroding boundaries. Ponsford et al. (109) modified this theory to explain school interventions for substance use and violence prevention, taking more account of the role of direct inputs on risk and protective factors for the primary outcome. Our ToC drew on both Markham and Aveyard's (61) and Ponsford et al.'s (109) work and our theorized mapping of our program (Figure 4) is explained fully in Supplementary File 10.

## 4 Discussion

Schools as sites for public mental health programs for adolescents are endorsed by the WHO (33), and the Indian National Health Policy (110) identified schools as a key location for improving the physical and mental health of young people. However, despite high rates of mental health related youth suicide, India is still without a whole school approach for adolescent anxiety and depression. Our study aimed to progress efforts toward a sustainable whole school program for Indian secondary schools by first working with local communities to co-produce a culturally relevant, evidence-based, acceptable, and feasible program.

Transparent and comprehensive reporting of the processes and outcomes of intervention co-production are important to future implementation, testing and upscaling (111). Following the MRC guidance for the development of complex interventions (112), and using the ADAPT framework (67), we drew on national and international evidence of what works and culturally adapted this with local schools (teachers, headteachers, and students), parents and mental health professionals to assemble a whole school program, with a primary aim of reducing adolescent anxiety and depression. We generated logic models and a provisional integrated program theory of change. Our paper reports the final whole school program content and implementation plan ready for feasibility testing [as per Hugh-Jones et al. (63)]. Here we discuss several issues emerging from the intervention development process and their implications.

### 4.1 Key findings from desk reviews

First, our multi-level needs assessment (ADAPT Step 1) identified the importance of school programs upholding and strengthening Indian values, including: the respect and power afforded to teachers; being sensitive to the risk of mental health stigma when raising mental health awareness; addressing adolescents' stress from multiple sources; using experiential and solution-focused approaches especially to cope with interpersonal problems and to ensure functional outcomes (academic performance and attendance).

Second, our systematic and umbrella reviews (ADAPT Step 2) to identify candidate programs for cultural adaptation within a whole school approach in Indian secondary schools revealed that, in general, effectiveness data is lacking, effectiveness studies are often limited by poor reporting, and there is low availability or access to shared intervention manuals/resources for adaptation. We could only identify two programs that were suitable to inform our SAMA for Youth component [SEHER (5859); Shamiri (113)] indicating that despite the global proliferation of school-based programs, there remains a significant problem in evidence generation for prevention of anxiety and depression and/or in the suitability or sharing of programs for adaptation. We were able to identify candidate interventions for teacher mental health literacy, and one evidence-based intervention and several Indian resources for school climate that could inform our approach. However, lacking were evidence-based, accessible and culturally adaptable programs to provide skills that could help to reduce teachers' use of corporal punishment and to promote parent mental health literacy. Therefore, to create our intervention prototypes, we had to also draw upon syntheses of common components of effective adolescent school mental health programs (91–94) as well as Indian and global resources for programs that appeared adaptable but were yet to be established for effectiveness.

### 4.2 Key findings from co-adaptation

All stakeholders broadly endorsed a whole school program focusing on mental health promotion for youth, mental health literacy for students (starting with Grade 9 for feasibility), teachers and parents, improving school climate, including reducing harsh discipline by teachers and implementing anti-bullying and safeguarding policies. That lay counselors were the preferred delivery agent across all of our participating stakeholders is in line with other reports (56, 59). Although Indian polices such as Ayushman Bharat (53), recognize teachers as a resource for student mental health, the ground realities are that teachers are overburdened and lack time and material resources, and that task-shifting to a lay counselor is a scalable option for health promotion in Indian schools. Evidence indicates that lay counselors can contribute to the advancement of public mental health in LMICs via delivery of psychoeducation and counseling, reducing stigma and creating a bridge between communities and mental health professionals—essentially a first-tier mental health service (114, 115). Reflecting the WHO (116) guidance on task-shifting, our participating schools underscored the importance of clearly defining the role of the lay counselor, how they would be inducted to work with the unique values of each school, how they would collaborate with teaching staff, and their place in a robust systems of supervision and referral. Adolescents also had expectations of the lay counselor—namely that they would create relaxed, fun, and safe relationships with them. Our findings show that whilst task-shifting is a valuable option, the introduction and embedding of the lay counselor in a schools' social dimension needs to be well planned, resourced, and managed.

Adolescents had a number of priorities for the program, including that it be delivered in active, participatory and experiential ways. This preference is now a consistent finding (59, 117) meaning that school mental health programs will likely be most acceptable if they provide practical strategies that help adolescents manage many everyday hassles and worries in their lives. Adolescents wanted a program that was fun and different to everyday classes. They also wanted the lay counselor to offer help without breaking confidentiality, mirroring findings in the development of SEHER (59). Support that validates young people's desire for privacy and carefully balances their emerging individuation from parents whilst fostering trusting relationships with them, remains a complex element of school mental health and school counseling programs in general (118). In response, in our study, clarity of the lay counselor duties and processes following disclosure of significant risks (including when parents might be contacted) was emphasized in the co-development of the accompanying SAMA safeguarding policy (forthcoming), supporting adolescents' agency in deciding what to tell a lay counselor. Adolescents also wanted SAMA to address teacher behavior which impacts their wellbeing, namely favoritism and what they termed teacher bullying (referencing harsh/corporal discipline and teaching by humiliation or fear). Such practices are consequential and often remembered into adulthood (11).

Whist there was considerable cross-stakeholder consensus on what should be included in a program targeting adolescents (namely content which empowers adolescents to manage their wellbeing by equipping them with mental health literacy, problem solving skills and clear routes for help), there were areas of departure. Parents, teachers, and mental health professionals wanted the SAMA for YOUTH intervention component to focus on issues that adolescents had not raised, including technology addiction, living by values, academic focus and dealing with family issues. In contrast, adolescents reported they would not be comfortable discussing boy-girl relationships or family issues as part of a school mental health program. There were some gendered preferences amongst the adolescents themselves, Whilst the prototype content was attractive to all of our adolescent participants, boys prioritized more individual focused content (self-confidence, goal-setting, managing stress) whereas girls were keen that we include social focused content (managing difficult friendships). This underlines the importance of asking Indian young people of different ages and genders what they would most like help within a school program designed to serve them. Such consultations with young people for the creation of school mental health programs are established in some countries and emergent in others [e.g., UK, 121)]. The final SAMA for YOUTH programs contains many of the evidence-based active ingredients for prevention and early intervention in youth depression and anxiety documented by the Wellcome Trust's reviews (95, 96), including behavioral activation, problem-solving, relaxation techniques, emotional awareness and regulation, decentering, addressing repetitive negative thinking, improving social relationships and mental health literacy.

That all stakeholders endorsed the SAMA for Teachers intervention prototype points to a collective, local perspective that any whole school mental health program must include teacher wellbeing, teachers' relationships with students and teachers' use of positive rather than harsh discipline practices. As with all teacher programs, a concern was how to make it feasible to work and life schedules, the potential of emboldening the seriousness of the training by linking schools to work together, and how to credit teachers for engaging in this continued professional development. Notably, promoting teacher MHL was well accepted, including the role teachers could take up in identifying adolescent mental health needs, as long as it was delivered by a mental health expert rather than lay counselor, who would be judged as unsuitable to be guiding higher status professionals.

The SAMA for School intervention was also endorsed by all stakeholders, with its anticipated success contingent upon broad and continued school awareness of the purpose of the intervention work. This intervention component elicited diverse views in co-adaptation about what should be included, how intense and comprehensive it should be, and how it might deliver for different stakeholder interests (e.g., adolescents girls wanted a focus on improving school relations whereas headteachers considered the public facing positive reputation this could bring the school and how critical it was to communicate well with parents about this). Mental health professionals also stressed lay counselor training and protocols should address student anonymity and confidentiality and decision-making for referrals. Notably, the adult stakeholders supported the establishment of youth-led school committees.

Consultations around evaluating the program showed acceptability to measure adolescent anxiety, depression and wellbeing but highlighted the pitfalls that should be avoided, namely that students would perceive the completion of measures as an exam with right and wrong answers or would not fully understand the measure items. Adolescent boys suggested individual or group discussions as a good data collection method. Given the sensitivity of asking teachers to disclose their use of corporal punishment, evaluation of SAMA for Teachers was suggested to use responses to vignettes.

### 4.3 The final whole school program

Our final co-created, whole school SAMA prototype, ready for feasibility testing, comprises four manualized interventions targeting adolescents, teachers, parents and the school environment, designed to be delivered over 6–12 months by an embedded lay counselor and external mental health professionals. It is multi-component, targets mental health risk and protective factors across actors and systems, and reflects six of the eight global standards for the WHO's and UNESCO's Health Promoting Schools (15) (school policies and resources; school governance and leadership; school and community partnerships; school curriculum; school social-emotional environment and school health services). It also aligns with many agendas and recommendations in Indian education and mental health policies (13, 14, 52), as summarized in our review of relevant policies (119).

Building on the foundational work of SEHER (59), we consider our SAMA program to balance complexity with utility and feasibility. Our program integrates evidence of what works with culturally sensitive community preferences, needs, and values. Overall, Indian adolescents appear to want the same from a school wellbeing programs as young people in other countries, namely for a school culture that is young person friendly, with egalitarian and psychologically and physically safe relationships, in which adolescents have some agency and can access creative and actionable knowledge about mental health and wellbeing, with due care for sensitive issues and needs (120). Our ToC attempts to capture this, drawing upon the work of Markham and Aveyard (61), which argues that adolescent health is promoted when their holistic experience of school equips and motivates them to autonomously choose to function well and flourish, and directs school mental health programs away from only focusing on prescriptive psychoeducation of young people. Within our ToC, our SAMA program is theorized to work across intraindividual, interindividual, and organizational processes, working together to foster a caring school environment centered on positive relationships, psychological safety and skills to manage problems, whilst also working to directly reduce known risks to youth mental health. Whilst globally the need for relational orientations in safe and supportive school environments is not new, actionable interventions to promote this in India and elsewhere remain rare.

### 4.4 Next steps

We have initiated feasibility testing of the SAMA whole school program, as per Hugh-Jones et al. (63) protocol in eight schools in the state of Karnataka, India. We include both government and non-government schools across rural and urban areas, documenting school characteristics which may explain any variation in feasibility. As Duncan et al. (111) sets out, as well as establishing feasibility, such studies should be considered another part of the iterative cycle of intervention development, where we identify problems, solutions, and creative additions, as well as potential unintended harms of the intervention. Having four inter-related interventions in one program allows for refinement in one without dependencies on another. Recognizing schools as complex social adaptive systems (65), we anticipate flexible rather than rigid implementation (121), which requires empowering delivery teams to innovate “on the job” (within-systems innovation), and to address missed or non-prioritized key implementation determinants during implementation (121, 122). Planned process evaluations for the feasibility study will help us to understand participants' experiences of each intervention, mechanisms of change, unintended consequences or harms, and areas for improvement. The feasibility study will also aim to identify the best primary outcome measures for adolescent anxiety and depression. That work has begun and is sensitized to both varied adolescent literacy levels and the importance of culturally valid measures (123). Identifying what is needed to sustain the program if effectiveness is established is a future key concern. We have begun preliminary cost-effectiveness estimates [as per Hugh-Jones et al. (63)] which will inform fuller analysis in the feasibility study. Finally, that adolescent boys and girls may need different things from a whole school mental health program was an important consideration for us. However, knowledge gleaned on the importance of gender, and also gender identity, from the review and co-production stages was minimal and we still have much to understand about this. This consideration is distinct from the need to optimize school programs to promote gender equality, which remains a significant challenge in India, with consequences for mental health and economic and social wellbeing (124). India To optimize how SAMA might, in future, embed more work toward gender equality, we have conducted a systematic review of Indian school-based interventions which aim to promote gender egalitarian attitudes and behaviors among adolescents (83). Our protocol for the feasibility study indicates that we will analyse for gender effects.

Looking ahead, we anticipate that the SAMA whole school program could be scaled in a phased manner. Our feasibility study will need to be followed by a definitive effectiveness trial, expanding to more diverse regions and including more varied school types, especially under-resourced and marginalized communities. Since India is a multilingual and multicultural country, materials will be translated and culturally adapted for different states, preserving the core components. Such work would require substantial resources. If the program demonstrates effectiveness, later expansion would require inter-sectoral coordination, sustained financing and state-level adaptation. Upscaling will need to be planned in alignment with existing national programs and policies. To support progress, we have conducted a mental policy review and interview study with Indian policy actors to understand the uptake of evidence into policy (119).

Key national and state-level enablers include existing school-health platforms, teacher training structures, and digital capacity-building tools. Potential barriers include inter-state variation in administrative capacity and financing, language and cultural diversity, stigma, limited buy-in from education and health departments, and shortages of mental health professionals. To address these, we propose early adoption in states with stronger infrastructure, creation of state and district hub teams and master-trainer cascades to reduce dependence on specialists, and embedding monitoring frameworks into routine reporting systems. Long-term sustainability will be pursued integration into pre- and in-service teacher training, convergence across health and education departments, and strategic use of public–private partnerships or corporate social responsibility funding in initial waves until recurrent state funding is secured.

### 4.5 Strengths & limitations

By adopting the ADAPT framework, our study demonstrates a systematic approach to combining evidence with the needs and aspirations of target communities in the creation of a whole school mental health program for secondary schools in India. We included those often excluded from co-production of school interventions (e.g., rural schools) and, in adopting Lundy's model (80), ensured that the perspectives and needs of Indian adolescents were elicited and respected. We respect open research practices by sharing comprehensive and detailed data generation and decision reporting in Supplementary material. Comprehensive and transparent reporting of our intervention development process may assist future understanding of success or failure and full details of the intervention content (as per TIDierR checklist, Supplementary File 10) may assist others in building on this work. We report the involvement of all stakeholders via the Guidance for Reporting Involvement of Patients and the Public (GRIPP 2) (125) in Supplementary File 11.

Findings should be considered in light of several limitations. Our reviews of best available evidence highlighted a dearth of knowledge about what works in LMICs. Our co-production stage was limited to one Indian state and eight schools, who are likely to have been particularly motivated to promote student wellbeing. Our approach to recruiting schools for co-design may have introduced selection and participation bias, although our purposive recruitment of government and rural schools enhances the relevance of findings for low-resources schools. Perspectives of schools who are less positive about school wellbeing programs, are who are highly resource-constrained, are lacking. Adolescent and adult literacy in India is highly varied, shaped by socio-economic, gender and regional disparities (85). This can present challenges for inclusion and participation in research. Whilst we invested considerable effort in creating co-production workshops activities not reliant on high levels of literacy, and conducted them in the dominant local language, it is possible that some adolescent and parent/carer participants felt less able to contribute than others (although overall engagement was high across all groups). Using ADAPT (67) (i.e., systematically building on evidence and taking this to cultural co-adaptation and co-production with local communities) was very resource intensive. In addition, considerable resources were required to deploy the multiple available frameworks and standards in participatory practices, co-production, and intervention design. Resource demand has been noted by others attempting to develop evidence-based school interventions for low resource settings (126).

### 4.6 Conclusions

Our approach advocates the importance of generating a whole school approach to mental health promotion for adolescents in India by combining available global evidence with local stakeholders' values, need and preferences and in ways that align with regional and national policies and initiatives relevant to adolescent mental health. Stakeholders' perspectives in co-production were crucial in identifying which program content and implementation strategies could work, and which should be avoided, and which program components could be led by an embedded lay counselor and which needed to be delivered by external professionals. Whilst there were some competing priorities among our stakeholders, there was comprehensive and strong endorsement for an integrated suite of interventions targeting multiple risk and protective factors, directed at adolescents, teachers, parents and school environment. The final co-adapted and co-produced SAMA whole school program will be taken to a feasibility trial as per our protocol, where we will learn how to train, embed and support lay counselors for delivery of this program (63).

## Data Availability

The original contributions presented in the study are included in the article/Supplementary material, further inquiries can be directed to the corresponding author.
