# Supplementary material for: Investing in youth public mental health in India: multi-stakeholder co-production of a whole school program to promote the mental health of Indian adolescents
Source: Front Public Health. 2025 Nov 28;13:1670439. doi: 10.3389/fpubh.2025.1670439 (PMC12698484; doi:10.3389/fpubh.2025.1670439)
Supplement: Supplementary file 1 [file Table_1.docx]

## SUPPLEMENTARY FILES

CONTENTS

**Supplementary File 1. Quality Intervention Development**

Supplementary File 1. Quality Intervention Development

***Steps 1 – 3 of the Six Steps of Quality Intervention Development (Wight et al., 2016).***

As part of the funding bid for this study, we followed steps 1 –3 of the Six Steps in Quality Intervention Development (6SQuID) (Wight et al., 2016) which allowed us to set out our position with regards to the nature of the problem and its causes, along with which causal or contextual factors might have greatest scope for change.

**Step 1: Define the nature and causes of the problem**

| **Questions** | **Answers** |
| --- | --- |
| **Nature and extent of main problem** | |
| What is the nature and extent of the problem? | India homes the largest number of adolescents in the world (Unicef, 2019). Of adolescents aged between 13 and 17 years in India, an estimated 9.8 million have clinical mental health disorders (pooled prevalence: 7.3%) (Gururaj et al., 2016). Alongside this, India has one of the highest youth suicide rates in the world, with suicide being a leading cause of adolescent death (Patel et al., 2012). Depression prevalence in school-going adolescents has been found to be between 3%–68% (Grover et al., 2019). Anxiety prevalence rates have been found to be 3.6% (National Mental Health Durvey of India, 2016). |
| For whom and at what levels does the problem exist? | Anxiety and depression are high in both male and female adolescent in India, with some studies reporting higher rates in females (e.g. Jayashree et al., 2018; Sandal et al., 2017) and some reporting higher rates in males (e.g. Deb et al., 2010). It is suggested that although males and females both experience high levels, they may manifest differently due to different gender roles and expectations (e.g. Ram et al., 2014). Anxiety and depression have also found to be high at all SES levels, however data on this is contradictory, with some studies reporting higher rates in higher SES groups (e.g. those attending private schools) and some in lower SES groups (e.g. Mishra et al., 2018). |
| What is the history? | As only one national mental health survey has been conducted in India (2016) the change over time is currently unknown (Gautham et al., 2020) |
| Causes and contributing factors | **Individual:**  **Poor mental health literacy;** lack of knowledge of mental health among Indian adolescents contributes to high levels of disorder (e.g. Attygalle et al., 2017; Ogorchukwu et al., 2016).  **Academic pressure;** Young people in India report extreme academic stress (Arun et al., 2017) resulting in high school related anxiety and suicide attempts (Deb et al., 2020; Verma & Gupta, 1990).  **Bullying**; Bullying and victimisation is widespread among Indian adolescents in schools and has significant impacts on mental health (e.g. Malhi et al., 2014; Malik; 2016).  **Substance use**; Substance use among Indian adolescents has increased significantly in the past two decades, leading to mental health disorders and suicide (Kumar, 2020; Sahu, 2012).  **Low access to resources**; Resources for mental health are scares and inequitably distributed in India, enhancing the problem of mental ill-health, as young people do not have access to adequate support (e.g. Patel et al., 2008; Saxena et al., 2007).  **Interpersonal:** Low teacher mental health literacy has also been found to enhance the problem of adolescent mental health in India (e.g. Venkataramen et al., 2019), with high levels of teacher corporal punishment negatively impacting young people’s mental health (e.g. Deb et al., 2017).  **Community/ Sociocultural:** Cultural views on mental health and high levels of mental health stigma, including discrimination in schools, have been found to lead to low help seeking behaviours (e.g. Kumar, 2019). Additionally, community initiatives in schools are slow, with initiatives mostly targeting physical health, rather than mental health (e.g. Srikala et al., 2010). |
| **Which causes are more important?** | |
| Consequences for those directly affected? (young people) | *Immediate*: anxiety, depression, other mental health conditions; poor school performance; low self-esteem; low quality of life; school absence. *Long-term*: chronic mental health condition; suicide risk; low economic and life opportunities; poor relationships; risky behaviours; substance abuse; stigma; social isolation / exclusion. |
| What are the consequences for those indirectly affected? | Peers may be affected by feeling disconnected to the person affected; may exclude / bully / stigmatise them.  Parents / family may worry, not knowing how to respond, feeling stigma. May exacerbate the problem by further school pressure.  Teachers may be affected by not knowing how to respond to the young person; may exacerbate the problem by further school pressure or harsh discipline.  Schools may be affected by low performance and stigma.  Community may be affected by burden on mental health services.  Country may be affected by economic costs of care of youth affected by anxiety and depression; unable to benefit from the potential of youth population. |

**Step 2: Identify modifiable factors at school and family level**

| **Factor** | **Evidence that is modifiable** |
| --- | --- |
| Youth mental health literacy | Interventions to improve multiple areas of life for young people, including mental health literacy: Balaji et al. (2011).  Interventions to improve adolescent girls’ mental health awareness: Das et al. (2010).  Interventions to improve adolescent mental health: Balaji et al., (2011). |
| Youth help-seeking | Classroom based program to increase adolescent help seeking behaviour: Parikh et al. (2021). |
| Teacher mental health literacy | Interventions to improve teacher mental health literacy:  Kumar (2011); Naik et al.(2015); Shah et al.(2011). |
| Parent mental health literacy | Parental group intervention to increase parental understanding of adolescent mental health: Janardhana & Manjula (2020). |
| School climate | SEHER Program e.g. Shinde et al. (2020).  School mental health program to improve young people life in multiple aspects in school: Srikala & Kishore (2010). |

**Step 3: Anticipated change mechanisms at school and family level**

| **Factor** | **Change mechanisms** | **Is this sufficient to reduce the problem?** |
| --- | --- | --- |
| Youth mental health literacy | Curriculum based education which includes new knowledge, self-awareness and actionable strategies to manage mental health; de-stigmatising mental health; promoting positive peer relationships. | Possibly but better in conjunction with other components. |
| Youth help-seeking | Curriculum based education; Easy access to support; de-stigmatising support-seeking; providing effective support. | Yes, but not on its own. |
| Teacher mental health literacy | Psychoeducation to understand adolescence as a period of vulnerability to the onset of mental health conditions; de-stigmatising mental health conditions. | Yes, but not on its own. |
| Teacher behaviour / class management practices | Developing insights into effects of harsh school practices; being equipped with new skills for positive practices; strengthening capacity and motivation to implement this new knowledge. | Yes, but not on its own. |
| Parent mental health literacy | Psychoeducation; strengthening capacity and motivation to implement this new knowledge. | Possibly but least know of modifiable factors. |
| School climate | Whole school changes to policy and practices around addressing bullying, promoting positive school activities; promoting youth voice; promoting school connectedness and belonging. | Possibly but may face the most implementation challenges. |

**References**

Arun, P., Ramamurthy, P., & Thilakan, P. (2022). Indian medical students with depression, anxiety, and suicidal behavior: why do they not seek treatment? *Indian Journal of Psychological Medicine*, *44*(1), 10-16.

Attygalle, U. R., Perera, H., & Jayamanne, B. D. W. (2017). Mental health literacy in adolescents: ability to recognise problems, helpful interventions and outcomes. *Child and Adolescent Psychiatry and Mental Health*, *11*, 1-8.

Balaji, M., Andrews, T., Andrew, G., & Patel, V. (2011). The acceptability, feasibility, and effectiveness of a population-based intervention to promote youth health: an exploratory study in Goa, India. *Journal of Adolescent health*, *48*(5), 453-460.

Das, P., Pal, R., & Pal, S. (2010). Awareness on psychosomatic health among adolescent girls of three schools in north Kolkata. *Indian journal of psychiatry*, *52*(4), 355-359.

Deb, S., Chatterjee, P., & Walsh, K. (2010). Anxiety among high school students in India: Comparisons across gender, school type, social strata and perceptions of quality time with parents. *Australian Journal of educational and developmental psychology*, *10*, 18-31.

Deb, S., Kar, S., Deb, S., Biswas, S., Dar, A. A., & Mukherjee, T. (2022). A cross-sectional study on mental health of school students during the COVID-19 pandemic in India. *Data*, *7*(7), 99.

Deb, S., Sathyanarayanan, P., Machiraju, R., Thomas, S., & McGirr, K. (2017). Are there differences in the mental health status of adolescents in Puducherry?. *Asian journal of psychiatry*, *27*, 32-39.

Gautham, M. S., Gururaj, G., Varghese, M., Benegal, V., Rao, G. N., Kokane, A., ... & Shibukumar, T. M. (2020). The National Mental Health Survey of India (2016): Prevalence, socio-demographic correlates and treatment gap of mental morbidity. *International Journal of Social Psychiatry*, *66*(4), 361-372.

Grover, S., Raju V, V., Sharma, A., & Shah, R. (2019). Depression in children and adolescents: a review of Indian studies. *Indian Journal of Psychological Medicine*, *41*(3), 216-227.

Gururaj, B., Satishkumar, M., & Kumar, M. K. (2016). Analysis of factors affecting the performance of exports in India. *International journal of agriculture, environment and biotechnology*, *9*(4), 613-616.

Janardhana, N., & Manjula, B. (2020). Parental-group interventions for parents of children with mental health problems admitted in a tertiary care center: An experience from India. *Indian Journal of Community Medicine*, *45*(1), 48-53.

Jayashree, K., Mithra, P. P., Nair, M. K. C., Unnikrishnan, B., & Pai, K. (2018). Depression and anxiety disorders among schoolgoing adolescents in an urban area of South India. *Indian Journal of Community Medicine*, *43*(Suppl 1), S28-S32.

Kumar, A. (2011). Mental health services in rural India: challenges and prospects. *Health*, *3*(12), 757-761.

Kumar, M. M., Pathak, V. K., & Ruikar, M. (2020). Tribal population in India: A public health challenge and road to future. *Journal of Family Medicine and Primary Care*, *9*(2), 508-512.

Malhi, P., Bharti, B., & Sidhu, M. (2014). Aggression in schools: psychosocial outcomes of bullying among Indian adolescents. *The Indian Journal of Pediatrics*, *81*, 1171-1176.

Malik, A., & Mehta, M. (2016). Bullying among adolescents in an Indian school. *Psychological Studies*, *61*, 220-232.

Mishra, S. K., Srivastava, M., Tiwary, N. K., & Kumar, A. (2018). Prevalence of depression and anxiety among children in rural and suburban areas of Eastern Uttar Pradesh: A cross-sectional study. *Journal of family medicine and primary care*, *7*(1), 21-26.

Naik, V., Nithyananda, S., Chandrakanth, C., Virupaksha, H. G., Reshma, B. K., Basavaraj, S., ... & Amaresha, A. C. (2015). School mental health programme for teachers recruited through corporate social responsibility: A quasi experimental study. *Indian Journal of Mental Health*, *2*(3).

National mental health survey of India. (2016). [002076402090794120240407-1-fkghsv-libre.pdf](https://d1wqtxts1xzle7.cloudfront.net/113107823/002076402090794120240407-1-fkghsv-libre.pdf?1712503181=&response-content-disposition=inline%3B+filename%3DThe_National_Mental_Health_Survey_of_Ind.pdf&Expires=1732620455&Signature=X1~J3Je9fbZTtlFajk-U~m8b1cDZBbulsYooGxhZEtR~V6tAW2zgkpMXoinM7BYhcRwnF-WzOb24prgu3QBOgQ4ddYCsUYG87txhfhNJdg4BNAHt1--sf2pQ1XcV2BM~Cyr47ot6Kmctwz3ZcAjDliR3QjGAX3qD-VC5g4ZAsc6TUPrRhZzG~3R65muniF4-Xicx7lqHsqvpelY6FtMGde1Ep5IwxAMOx~-IH9dK8db--AHlygFG9LHdoGJRriM3~OxaSYu25VUjRfPmnnY4TN87q3kaQ7EEIrt12TlZIr2tyJXUW0G1YXCO-JPqNUbtm7cnGhRL-6g40jfAco0sIw__&Key-Pair-Id=APKAJLOHF5GGSLRBV4ZA)

Ogorchukwu, J. M., Sekaran, V. C., Nair, S., & Ashok, L. (2016). Mental health literacy among late adolescents in South India: What they know and what attitudes drive them. *Indian journal of psychological medicine*, *38*(3), 234-241.

Parikh, R., Hoogendoorn, A., Michelson, D., Ruwaard, J., Sharma, R., Bhat, B., ... & Patel, V. (2021). Increasing demand for school counselling through a lay counsellor-delivered classroom sensitisation intervention: a stepped-wedge cluster randomised controlled trial in New Delhi, India. *BMJ global health*, *6*(6), e003902.

Patel, V., Araya, R., Chowdhary, N., King, M., Kirkwood, B., Nayak, S., ... & Weiss, H. A. (2008). Detecting common mental disorders in primary care in India: a comparison of five screening questionnaires. *Psychological medicine*, *38*(2), 221-228.

Patel, C., Ramasundarahettige, L., & Vijayakumar. (2012). Suicide mortality in India: a nationally representative survey. *Lancet*, 379, 2343-2351

Ram, U., Strohschein, L., & Gaur, K. (2014). Gender socialization: Differences between male and female youth in India and associations with mental health. *International Journal of Population Research*, *2014*(1), 357145.

Sahu, K. K., & Sahu, S. (2012). Substance abuse causes and consequences. *Bangabasi academic journal*, *9*(12), 52-59.

Sandal, R. K., Goel, N. K., Sharma, M. K., Bakshi, R. K., Singh, N., & Kumar, D. (2017). Prevalence of

depression, anxiety and stress among school going adolescent in Chandigarh. *Journal of family medicine and primary care*, *6*(2), 405-410.

Saxena, S., Thornicroft, G., Knapp, M., & Whiteford, H. (2007). Resources for mental health: scarcity, inequity, and inefficiency. *The lancet*, *370*(9590), 878-889.

Shah, S. M. A., Kiani, K. M., Mahmood, Z., & Hussain, I. (2011). In-service training of secondary level teachers: A follow up of teachers’ performance in comparative perspective. *Journal of Education and Practice*, *2*(11), 40-50.

Shinde, S., Weiss, H. A., Khandeparkar, P., Pereira, B., Sharma, A., Gupta, R., ... & Patel, V. (2020). A

multicomponent secondary school health promotion intervention and adolescent health: an extension of the SEHER cluster randomised controlled trial in Bihar, India. *PLoS medicine*, *17*(2), e1003021.

Srikala, B., & Kishore, K. K. (2010). Empowering adolescents with life skills education in schools–School mental health program: Does it work?. *Indian Journal of psychiatry*, *52*(4), 344-349.

UNICEF. (2019). <https://www.unicef.org/media/90761/file/India-2019-COAR.pdf>

Venkataraman, S., Patil, R., & Balasundaram, S. (2019). Stigma toward mental illness among higher secondary school teachers in Puducherry, South India. *Journal of family medicine and primary care*, *8*(4), 1401-1407.

Verma, S., & Gupta, J. (1990). Some aspects of high academic stress and symptoms. *Journal of Personality and Clinical Studies*.

Supplementary File 2: ADAPT Step 2 Intervention Search and Selection

This file contains information on review search strategies, data screening, extraction and quality assessments as part of ADAPT Step 2.

For all reviews, searches were conducted for papers published between January 2011 - January 2022. Articles were required to be published in a peer-review journal. Searches were restricted to the last ten years to identify up-to-date systematic reviews. Language restrictions of either English or an Indian language known to the research team were applied (we did not include articles published in other languages). Electronic database searches were supplemented with a review of reference lists of included papers and forward-citation tracking of included papers.

**Review 1: The effectiveness of universal school interventions for prevention or treatment of adolescent anxiety and depression symptoms.**

Relevant databases (PsycINFO, PubMED, Cochrane Database of Systematic Reviews and Google Scholar) were searched to identify published systematic reviews of randomised controlled trials (including individual, parallel, cluster or quasi structure) with comparator controls (including ‘school-as-usual’ and active controls, counting that of waitlisting). Papers were eligible for inclusion if their primary outcome was a validated measure of anxiety and / or depression in 13-18 years-old; or a sample mean age of ≥13 years old). Interventions had to be delivered universally in a mainstream secondary school setting.

*PsycINFO Search Terms*

(Systematic review OR meta-analysis OR meta-analytic) AND (Adolescent OR adolescents OR young people OR youth) AND (Universal OR whole OR curriculum-based OR school-based OR program OR intervention OR early intervention OR program OR secondary school OR high school) AND (Depression OR anxiety OR mental health OR psychological) NOT Physical activity, green space, screen, self-harm, sexual abuse, alcohol, drugs, violence, tobacco, violence, HPV, HIV, diabetes, sleep, obesity, nutrition, yoga, indicated, COVID-19, primary, selective, targeted, autism, ADHD, PTSD, cancer, pain, neurodevelopment, eating disorders, indicated, selective, psychosis.

Filters Applied: Published 2011-Current; Peer Reviewed Journal; Adolescents 13-17 years old.

Hits 12/01/2022: 156

*PubMED Search Terms*

(Systematic review[Title/Abstract] OR meta-analysis[Title/Abstract] OR meta-analytic[Title/Abstract]) AND (Adolescent[Title/Abstract] OR adolescents[Title/Abstract] OR young people[Title/Abstract] OR youth[Title/Abstract]) AND (Universal[Title/Abstract] OR whole[Title/Abstract] OR curriculum-based[Title/Abstract] OR school-based[Title/Abstract] OR program[Title/Abstract] OR intervention[Title/Abstract] OR early intervention[Title/Abstract] OR program[Title/Abstract] OR secondary school[Title/Abstract] OR high school[Title/Abstract]) AND (Depression[Title/Abstract] OR anxiety[Title/Abstract] OR mental health[Title/Abstract] OR psychological[Title/Abstract])

Filters Applied: Published 2011-Current; Adolescents 13-17 years old.

Hits 12/01/2022: 347

*Google Scholar Search Terms*

allintitle: Systematic OR review OR meta-analysis OR meta-analytic AND adolescent OR youth OR young-people AND universal OR school-based OR program OR curriculum-based AND secondary-school OR high-school AND depression OR anxiety OR mental OR health -physical-activity, -green-space, -screen, self-harm, -sexual-abuse, -alcohol, -drugs, -violence, -tobacco, -violence, -HPV, -HIV, -diabetes, -sleep, -obesity, -nutrition, -yoga, -indicated, -COVID-19, -primary, -selective, -targeted, -autism, -ADHD, -PTSD, -cancer, -pain, -neurodevelopment, -eating-disorders, -indicated, -selective, -psychosis.

Filters Applied: Published 2011-Current.

Hits 12/01/2022: 20

*Cochrane Search Terms*

Systematic review OR meta-analysis OR meta-analytic” in Title Abstract Keyword AND Adolescent OR adolescents OR young people OR youth in Title Abstract Keyword AND Universal OR whole OR curriculum-based OR school-based OR program OR intervention OR early intervention OR program OR secondary school OR high school in Title Abstract Keyword AND Depression OR anxiety OR mental health OR psychological in Title Abstract Keyword NOT Physical activity, green space, screen, self-harm, sexual abuse, alcohol, drugs, violence, tobacco, violence, HPV, HIV, diabetes, sleep, obesity, nutrition, yoga, indicated, COVID-19, primary, selective, targeted, autism, ADHD, PTSD, cancer, pain, neurodevelopment, eating disorders, indicated, selective, psychosis in Title Abstract Keyword.

Hits 28/06/2021: 0

**Review 2: The effectiveness of interventions to improve secondary school teachers’ mental health literacy.**

Relevant databases (PsycINFO, PubMED, ERIC Cochrane Database of Systematic Reviews and Google Scholar) were searched to identify published systematic reviews of randomised controlled trials (including individual, parallel, cluster or quasi structure) with comparator controls (including ‘school-as-usual’ and active controls, counting that of waitlisting). Papers were eligible for inclusion if they had a primary outcome of mental health literacy and/or mental health knowledge of secondary school teachers or teacher mental health stigma. Primary outcomes needed to be measured by a validated instrument.

*Search terms (for all databases):*

Systematic reviews OR meta-analy* AND School* OR secondary school* OR high school* AND Teacher* OR staff AND Training OR education* OR workshop OR program* OR intervention AND Mental health literacy OR Mental health knowledge OR Mental health stigma NOT ADHD, ASD, Asthma, HIV, Exercise, physical exercise, chronic disease, eating disorder, athlete, parent, preschool, trauma, race, impairment, diseases, depression, anxiety, cancer.

**Review 3: The effectiveness of interventions to improve school climate in secondary schools.** Relevant databases (PsycINFO, ERIC, Cochrane Database of Systematic Reviews and Google Scholar) were searched to identify published systematic reviews of randomised controlled trials (including individual, parallel, cluster or quasi structure) with comparator controls (including ‘school-as-usual’ and active controls, counting that of waitlisting). Papers were eligible for inclusion if they had a primary outcome of school climate in secondary schools (targeting students and teachers in mainstream schooling). ‘School climate’ for the context of this study was conceptualised as latent property of the school spanning physical (perception of the material environment), cultural (the system of norms and rules), social (relationships between agents in the school), and individual (the attitude of students to learning and a sense of belonging to the school) elements of this environment. Interventions had to be delivered universally in a mainstream secondary school setting.

*Search terms (for all databases):*

Systematic review OR meta-analy* AND Secondary school* OR high school* AND Culture OR climate OR engagement OR safety OR connectedness OR mental health stigma OR stigma reduction OR environment NOT Sexual minority, ADHD, physical activity, counsellor, mindfulness, arrest, transgender, autism, ADHD.

**Review 4: The effectiveness of interventions to improve parent mental health literacy.** Relevant databases (PsycINFO, PubMED, Cochrane Database of Systematic Reviews and Google Scholar) were searched to identify published systematic reviews of randomised controlled trials (including individual, parallel, cluster or quasi structure) with comparator controls (including ‘school-as-usual’ and active controls, counting that of waitlisting). Papers were eligible for inclusion if they had a primary outcome of parent (including carers and guardians of adolescents) mental health, and a validated instrument was used to measure this.

*Search terms (for all databases):*

Systematic review* OR meta-analy* AND Parent* OR carer* AND Adolescen* OR youth OR young person OR young people OR teenager Mental health OR psychological OR emotional OR well-being OR depress* OR anxiety Training OR literacy OR education OR intervention OR program* OR support NOT bullying, chronic illness, migration, autism, ADHD, ASD, abuse, behav* problems, sports, PTSD.

**Data screening and extraction for all reviews**

Two reviewers independently screened title and abstract and full texts of included papers with conflicts resolved by discussion to reach a consensus. The screening process was managed within Rayyan (<https://www.rayyan.ai/>). Data extraction was supported by Microsoft Excel to allow data relating to citation details, objectives, participants, setting/context, intervention details, search details, sources searched, range of years, number of studies, type of studies, country of origin (or geographical reach), appraisal, appraisal instrument, appraisal rating, method of analysis, outcomes assessed, results, significance, heterogeneity to be collated following the JBI guidelines for data extraction for umbrella reviews. Data was independently double extracted by two researchers and cross-checked for accuracy.

**Quality Assessment**

Quality assessment was performed using the Cochrane Risk of Bias assessment tool. ‘Low’, ‘some concerns’, or ‘high’ risk of bias judgments were assigned to each domain and the overall quality. Two authors completed the process independently and disputes were resolved by consensus.

Supplementary File 3. Examples of Co-Production Workshop Activities

This file shows a selection of workshop activities used with adolescents to explore the prototype interventions, safeguarding, implementation and evaluation protocols***.***

**Example Activity #1: SAMA for YOUTH**

Adolescents were introduced to the concept of personas and asked to generate additional personas for us to hold in mind during co-production.


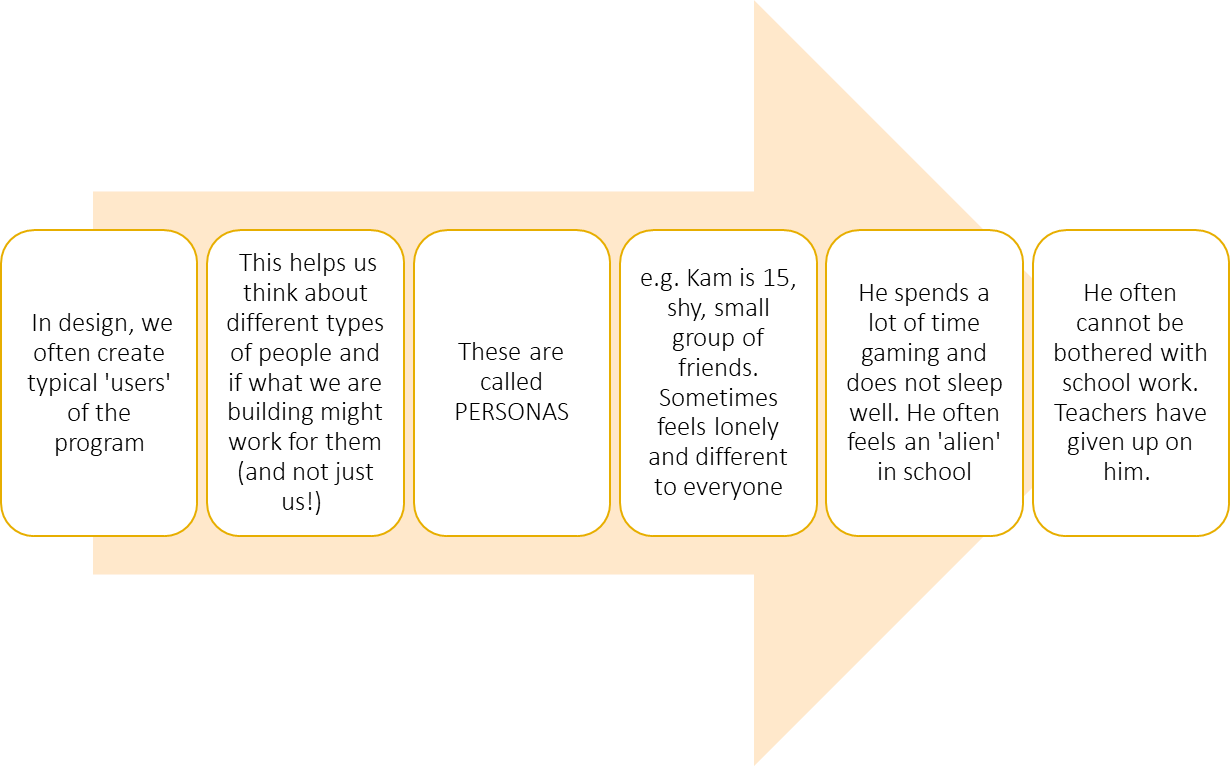


**Example Activity #2: Youth priorities for SAMA for YOUTH**

Adolescents were asked to place different coloured stickers to indicate their ratings for the intervention’s purpose.


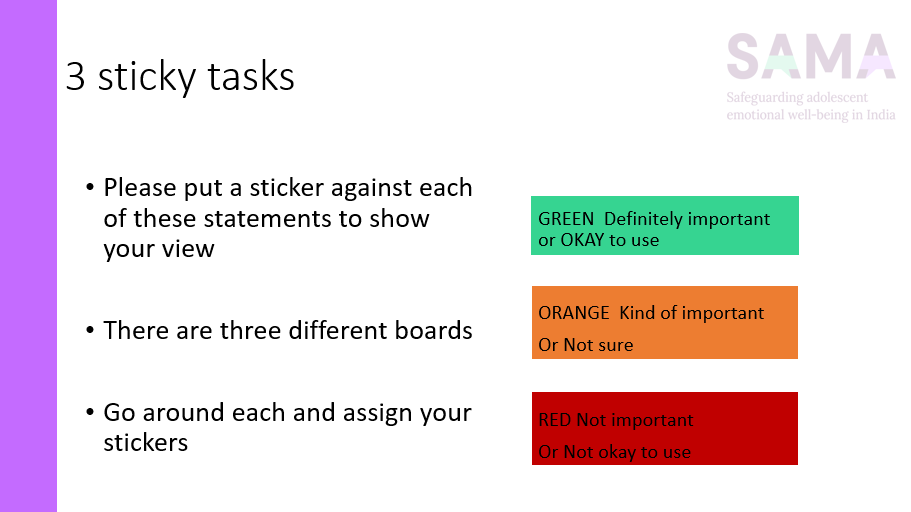


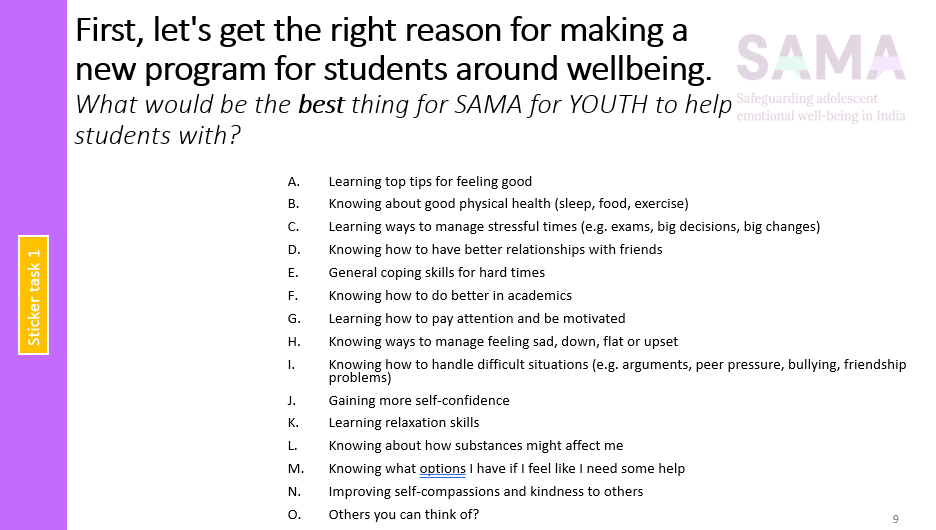


**Example Activity #3: Imagining one year after SAMA for YOUTH**

Adolescents were asked to imagine what might have influenced how much young people learnt from SAMA.


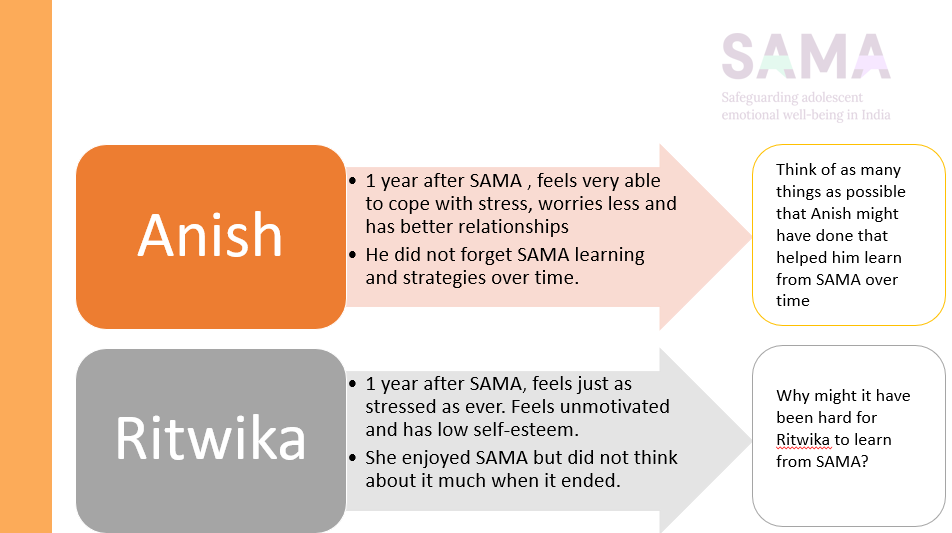


**Example Activity #4: Vignettes for youth-informed safeguarding protocols**

In groups, adolescents discussed three vignettes to explore youth perspectives on safeguarding protocols in SAMA.


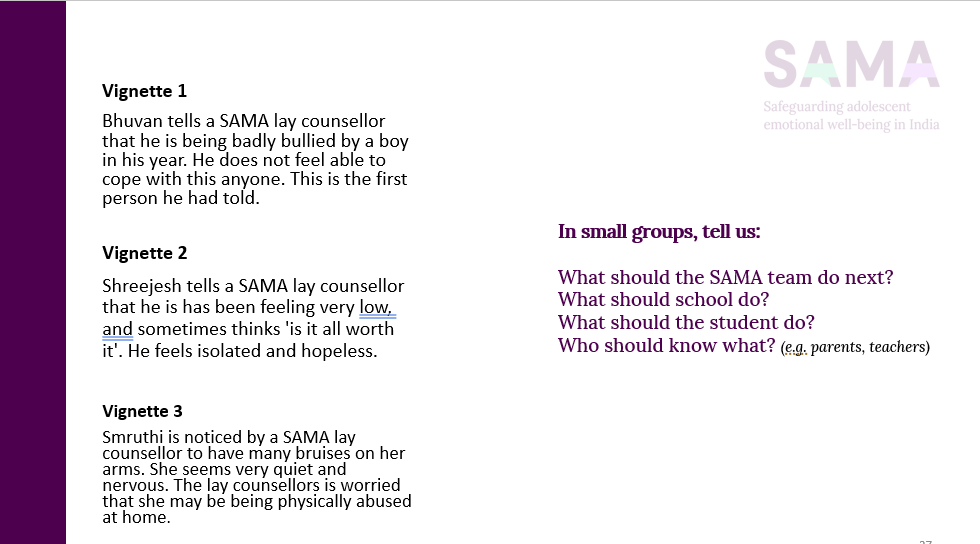


**Example Activity #5: Youth-informed views on researching the effectiveness of SAMA for YOUTH**

Adolescents choose to discuss whichever of the following questions they wanted to and joined the groups that was focusing on that issue.


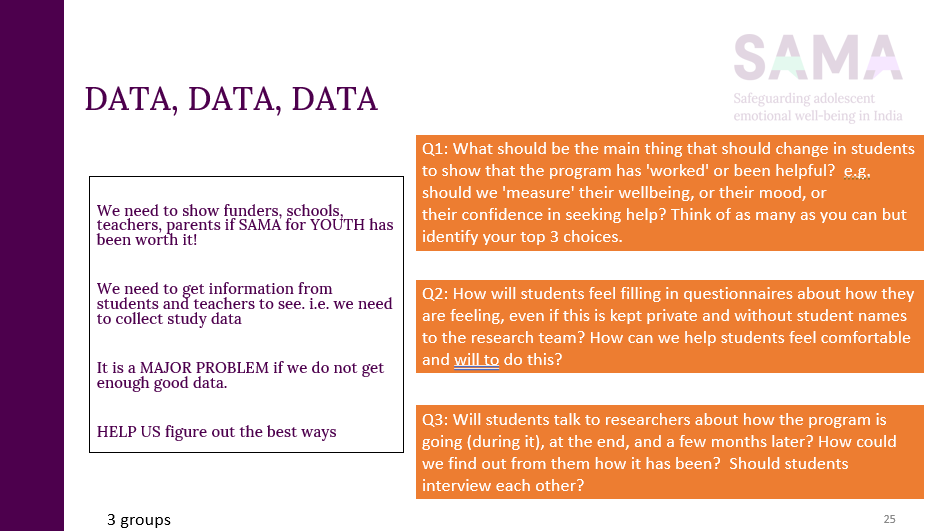


**Example Activity #6: Youth perspectives on SAMA for TEACHERS**

Adolescents were invited to draw or write about the perfect teacher. This was used to stimulate the teacher’s attitudes and behaviours that adolescents find supports their wellbeing in schools.


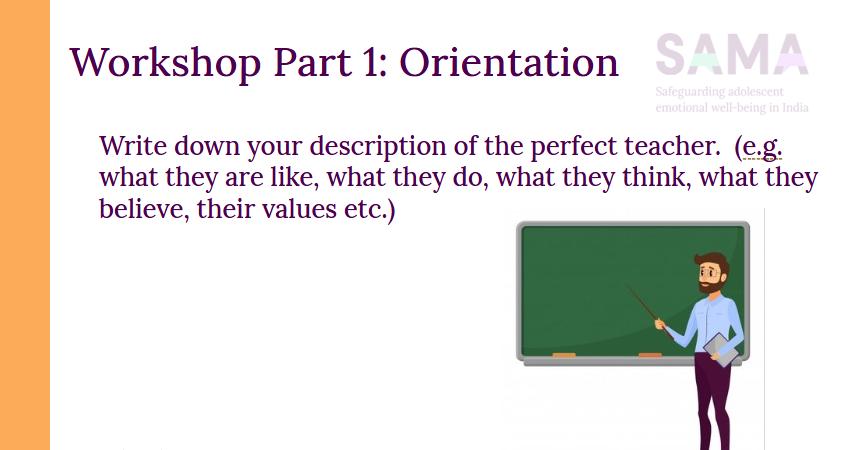


**Example Activity #7: Youth perspectives on SAMA for TEACHERS**

Adolescents were asked to give their perspectives on SAMA for Teachers and Teacher’s role in student mental health.


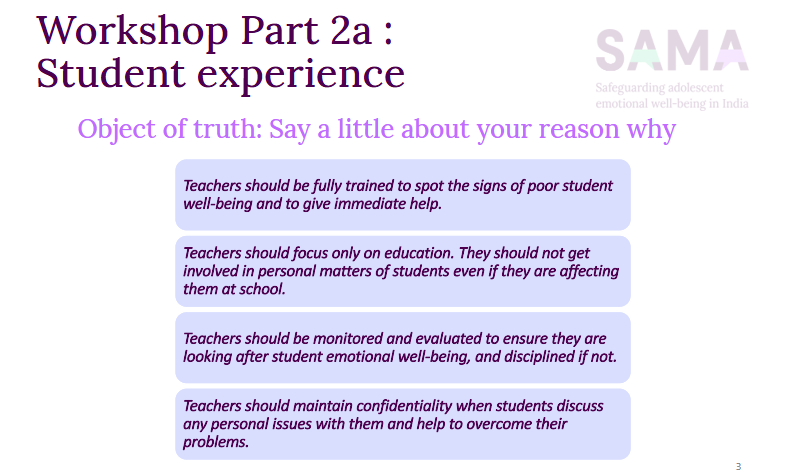


Supplementary File 4. Summary of key outcomes from each stage of our use of ADAPT

**Table S4.1:** **Summary of applying the ADAPT Framework to the development of SAMA whole school program.**

| **ADAPT Steps** | **Objectives** | **Outcomes** | **Learning / Decisions** |
| --- | --- | --- | --- |
| 1. Local Needs   Assessments  (ADAPT Step 1) | Map relevant local priorities and ongoing initiatives & policies | - Must not impact curriculum, exam, teacher or student workload - Experientially focused to support engagement and learning - Lay counsellors must be an asset to the schools - Must embed in school cycle for sustainability - Must address youth priorities and functional outcomes - Must recognise the power assigned to teachers by parents - Parent engagement sensitised to low literacy - Teacher components should align with teacher training curriculums | - School and educational authorities and policies are supportive of action on adolescent wellbeing where they adopt and strengthen Indian values and are likely to have impacts on youth functional outcomes (e.g. school performance, attendance, behaviour). - Sensitivity to mental health vocabulary is essential given stigma and fear of potential reputational damage to schools seen to be ‘needing’ mental health interventions. - High status of teachers in Indian culture should be recognised. - We published a review of Indian mental health policies to ensure alignment (Ivory et al., 2024) |
| 1. Evidence   Review (ADAPT  Steps 2-4) | Identify global and national evidence (content and implementation) available at the point of review on effective school-based programs which: |  | |
|  | 1. Prevent adolescent anxiety and / depression | - Very few effective whole school programs focusing on prevention - Very few intervention manuals accessible - Common effective elements (see Supplementary File 7) for individual focused interventions include emotion regulation, interpersonal skills, stress management and problem-solving - May be helpful to include mindfulness as one component - Could safely limit focus on addressing negative thinking (CBT effective for depression but cognitive restructuring not a clear-cut effective component of general well-being interventions) - Could include elements of social and emotional learning interventions which are inconsistently effective: self-management, responsible decision-making, self-awareness and social awareness | - Prevention approaches remain under-developed and under-researched (especially long-term effects) - Reporting and sharing of intervention content is poor - Knowledge of effective psychological approaches not always translated into programs with practical guidance for adolescents |
|  | 1. Promote teacher mental health literacy | - Very few effective interventions to promote teacher mental health literacy. - Effective elements to enhance literacy are information about mental health and illness, specific mental illnesses and stigma. - It may be helpful to add information on teacher wellbeing, promotion of help-seeking, and building resilience in students | - Effective ways to promote teacher mental health literacy in Indian teachers is under-researched. - Although there are resources to promote teacher mental health literacy, there is no evidence for outcomes. |
|  | 1. Promote teacher use of positive classroom practices | - There are very few evidence-based interventions to promote positive classroom practices in teachers. - Effective elements may include understanding the differences between positive practices and punishment, positive discipline practice techniques. | - The topic is under researched, especially in India. - Resources are available on enhancing teacher positive classroom practices do not have evidence for outcomes. |
|  | 1. Promote positive school environment | - Very few effective programs. - Whole-school and classroom activities were perceived as being helpful to promote mental health awareness, emotional wellbeing, connections and engagement. | - It is difficult to access manuals for programs that have claimed to have positive outcomes. |
|  | 1. Promote parent mental health literacy | - Very few effective programs. - ‘Parenting an adolescent’ is an element that is safe to add. | - Available resources are not associated with any evaluations. |
|  | Review evidence of delivery agents and effective implementation of whole school approaches. | - Lay counsellors can be effective delivery agents of whole school programs - An embedded intervention coordinator in school can promote intervention effectiveness - A school-based intervention committee can support intervention activities | - Implementation can be supported by lay counsellor + model, where they build school community engagement towards program ownership - Bring model to co-production stage |
|  | Review national and international safeguarding protocols for use in school mental health program and school-based research | - Sufficient global literature is available but there are significant differences between the context in which they have been developed and Indian schools. - Indian laws, regulations, and policies can drive safeguarding practice in schools; however, they are not easily understood by lay counsellors. | - Only one safeguarding protocol developed by an Indian organization was suitable and could be adapted to SAMA. This protocol was aligned with Indian laws and regulations. - Ensure lay counsellors understanding - Take to Step 6. |
| 1. Review of candidate intervention content   (ADAPT Step 5) | 1. To prevent adolescent anxiety and / depression | - SEHER and SHAMIRI were the only programs eligible for co-adaptation | - Combine programs and bring to co-adaptation and (as per Moore et al., 2021), extend with new content based on additional evidence of ‘what works’ for intervention for anxiety and depression and to promote wellbeing. Bring new prototype called SAMA for YOUTH to Step 6. |
|  | 1. To promote teacher MHL | - Go-To-Educator-Training was the only program eligible for co-adaptation. | - Combine components from other intervention sources on teacher mental health literacy. - Create the prototype called SAMA for TEACHERS and bring to Step 6. |
|  | 1. To promote teacher use of positive classroom practices | - A few resources were available but none were suitable for direct co-adaptation. | - Combine components from suitable resources on enhancing positive discipline practices and classroom management in teachers. - Create the prototype SAMA for TEACHERS and bring to Step 6. |
|  | 1. To prevent positive school environment | - Some resources were available although not directly relevant to Indian school - SEHER’s school climate intervention component was the only program eligible for direct co-adaptation. | - Combine some components from other resources with SEHER to create prototype SAMA for SCHOOLS and bring to Step 6. |
|  | 1. To promote parent MHL | - A small number of resources were identified but were inaccessible. | - Draw on our needs assessment, local knowledge and available evidence to develop prototype SAMA for PARENTS and bring to Step 6. |
| 4.Co-Adaptation / Co-Production  Workshops  (ADAPT Step 6) | Identify consensus and / or resolve barriers to a whole school approach | - Whole school approach welcomed by all stakeholders but likely to be sustained only if it enjoyed continued high profile of program in schools - Consensus to run four integrated interventions with staggered starts | - High-profile program launch scheduled in each school, with advance ‘teaser’ posters placed in schools. Schedule of continual within-school communications about program activities. |
|  | Determine consensus and / or improve proposed intervention aims and outcomes | - Consensus that aim should be to prevent anxiety and / or depression as well as to promote overall student levels of emotional wellbeing - Outcomes should include reduced bullying and attendance | - Proceed with planned primary aims - Extend outcomes to prevalence of bullying and self-reported attendance (to reduce burden on schools) |
|  | Determine if our prototype intervention content was acceptable or could be improved (adapted or new content co-produced) | - SAMA for YOUTH prototype was comprehensively endorsed. Wanted: engaging and fun activities; informal but confidential relationship with lay counsellors; practical and memorable skills and tips for stress and difficult situations; address teacher bullying and favouritism; address need for fun in school; have clear route for help seeking. Young people less concerned about self-esteem, gratitude and living with values. Adults requested coverage of substance use and romantic relationships. - SAMA for TEACHER prototype was completely endorsed. Suggested: including time and anger management, recommended: quarterly booster sessions, sessions (1.5-2 hours) every 2-3 weeks, scheduling between June-August, involving teachers from different schools, facilitating peer discussions, including all teachers, and convincing headmasters to support the program. - SAMA for SCHOOL prototype broadly endorsed. Uncertainty about inter-school communication and within-school committee feasibility (interest).  Suggested: success depends on ongoing awareness and communication, with an emphasis on non-stigmatizing language, clear roles for lay counsellors, and proper training on confidentiality and referral protocols. - SAMA for PARENTS prototype endorsed. Suggested: Intervention focus on sensitizing parents to adolescent needs and challenges, using accessible, culturally sensitive materials, addressing barriers like parents’ literacy, financial constraints, and time availability. | - SAMA for YOUTH with suggested new content / emphasis. Dropped coverage of values and gratitude, Opted to not include coverage of substance use, self-compassion, or self-esteem. - Lay counsellors training to emphasise confidentiality and its boundaries, and how to build trusting relationships in school - SAMA for TEACHERS prototype with some suggested new content and emphasis on peer discussion - SAMA for SCHOOLS - SAMA for PARENTS |
|  | Identify if our implementation plan, including lay counsellors) was acceptable and how to optimise | - Simultaneous delivery of SAMA for SCHOOLS with SAMA for YOUTH endorsed. Preferences for stepped delivery of SAMA for TEACHERS and SAMA for PARENTS - Lay counsellors endorsed with conditions (training, clear role boundaries and remit, effectively embedded in school) | - Final implementation timeline for each intervention component, cognisant of exam and holiday / festival breaks |
|  | Determine if the safeguarding protocol was feasible and / or could be improved | - Protocol approved, with emphasis on ensuring lay counsellors are well trained | - Train lay counsellors in safeguarding protocol |
|  | Identify if the proposed research methodology was feasible and / or could be improved | - Evaluation protocol accepted, including randomisation and ethics procedures - Ensure guardian consent information sent in local language - Ensure administration of youth measures is supported by staff | - All study materials made available in English and local language (Kannada) - Plan with data collection assistants how to support youth completion of measures |
| 5.Consultations with Steering Group, Youth Advisory Board and Independent Experts (ADAPT Steps 1 and 6) | Consult with project boards to ensure wide review of intervention prototypes and implementation plans | - Steering group approved prototypes. Suggested ‘train the trainer’ model and use of contemporary examples. - Independent experts approved the prototypes. Suggested: - SAMA for YOUTH: Reduce individualistic focus, increase attention to Indian values, use more age-appropriate examples. Address academic competence, need for extra-curricular activities and temperament. Reduce CBT and bring problem-solving earlier - SAMA for TEACHERS: Reduce the topics covered, value teachers’ inputs and understanding their experiences. Be ready to adapt material as you go. Consider how bodies store memories (corporal punishment) and practise stress release in sessions. - SAMA for PARENTS: Consider approaching mental health as physical health. Include triggers for adolescent ‘risky’ behaviour. Be ready to respond to parent vulnerabilities and anxieties | - SAMA for YOUTH increased focus on non-individualistic determinants of mental health but not academic competence nor temperament as not aligned with intervention aim. Incorporated suggestions for contemporary, age-appropriate examples. Added reflection on Indian and personal values. - Extra-curricular activities included under behavioural activation and doing what you enjoy. - SAMA for TEACHERS reduced content to ensure time for discussion and emphasis on valuing teachers and stress management. Did not include embodied ‘trauma’ as insufficient time to do so safely. - SAME for PARENTS framed mental health in similar ways to physical health; contextualised adolescent behaviour. |
| 6. Produce final intervention (ADAPT Step 7) | Based on all previous stages, the project team produced final versions of each intervention prototype, implementation protocol, evaluation protocol and safeguarding protocol. | | |

Supplementary File 5: ADAPT Step 1 multi-level needs assessment

This file shows the findings of the multi-level needs assessment for the ADAPT Step 1 intervention, outlining key considerations that researchers (SAMA Team) must consider when working with various stakeholders, including education departments, school authorities, staff, young people, and families.

**Table S5.1 ADAPT Step 1 Outcomes of the multi-level needs assessment**

| **Intervention** | **State Education Departments / Regional stakeholders** | **Schools** | **Young People** | **Families** |
| --- | --- | --- | --- | --- |
| **General** | Permission required to conduct work in schools.  Aligns with Indian policies on education and mental health. | Headteacher permissions required. | Assent required. | Consent required. |
|  | - Must respect value of educational achievement in schools; must respect the cultural positions of adolescence and teachers. - Delivered by lay counsellors to be an asset to the school. Must not require any resources from schools. - Must be sensitive to the fact that many mental health terms are stigmatised (e.g. anxiety, depression) and that school communities may perpetuate this stigma and be affected by it. - Embedding all work in school cycle for sustainability. - Must be a whole school program that the National Institute of Mental Health and Neurosciences can deliver in the future. | | | |
| **Youth focused intervention** | Be informed / involved in project. | - Does not impact on curriculum time or exam periods. - Does not over burden teaching staff. - Works with large classes. - Compatible with focus on academic achievement. - Avoid use of labelling of YP (school may then see them as problems). - Confidence that the work will have a credible impact in school. - Address priorities identified in Parikh et al (2019) i.e. conduct problems, such as attendance, exam performance, career prospects. They should have functional outcomes (especially academic functioning). - Address priorities identified by Vranda (2015) e.g. self-image/ peer pressure/ sexuality. | - For 15-year-olds, as reaching older adolescents will impact exam periods and will not be permitted. - Experiential. - Engaging multi-media rather than heavy text based. - Confidentiality. - Use of 1-2-1 counselling is acceptable. - Should not draw too heavily on cognitive-behavioral therapy as mind-body distinction not as culturally relevant. - Should address priorities identified in Parikh et al (2019) i.e. **practical** help with stress reduction/ coping with life difficulties (e.g. exams, future career, relationships, bullying). 1-2-1 counselling preferred over group sessions (counsellor: same gender/ slightly older than them/ warm/ friendly/ relatable). Confidentiality assurance. Face to face sessions (over digital) preferred. - Address priorities identified by Vranda (2015) e.g. knowledge of mental health, stress (e.g. exams) self-image, sexual abuse and harassment, peer pressure, sexuality, substance abuse career planning, gender related issues. | - Parents informed of safeguarding protocols. - Engaging multi-media rather than heavy text based. - Address priorities identified in Parikh et al (2019) i.e. increasing ‘good’/ obedient behaviors, exam performance and career prospects. Parents wanted updates on YP progress (conflicting with adolescent wanting confidentiality). - Address priorities identified by Vranda (2015) e.g. suicide prevention/ life skills |
| **Teacher focused intervention** | Need to see relevance to potential teacher training (NCERT, CBSE). | - Involve the school head during the planning stage. - Should not interfere with teacher main duties. - Recognise the power that parents give to teachers. - Should focus on teacher well-being as high levels of stress and burnout. - Intervention should be collaborative and experiential not text / resource heavy). - Include mental health education in culturally appropriate way. Should focus on (1) understanding how mental health / ill health manifests in young people ; (2) de-stigmatising and debunking myths; and (3) teachers feeling skilled to observe and notice young people and mental health in class. - Should be aware of mental health labels sometimes used by teachers (e.g. ADHD) and stigmatising language / labelling of young people (e.g. as problem students). - Deliver over time so teachers have chance to implement their learning. - Address priorities identified by Vranda (2015) e.g. Increasing teacher knowledge of mental health. |  |  |
| **School climate** | Need to explain how school psychological climate will relate to existing policy focus on the physical school environment and healthy behaviours by school staff. | Must not interfere with regular school activities. | - Must involve the students with appropriate permissions from school. - Should be experiential and not rely on written work. | To be informed about the activities. |
| **Parent intervention** |  |  |  | - Suitable for low-literacy levels. - Sensitive to stigma around mental health. - Respect cultural values around adolescent – parent relationships. - Address priorities identified by Vranda (2015) e.g. how to approach exam failure/ stress and school dropout with their child. |
| **Safeguarding** |  | - Need to involve school leadership in protocols. - Need non-stigmatising local referral pathway for mental health support; teachers, students and lay counsellors need to know this. - Consider role of local wellness centers. | | |

**Locally used vocabularies**

Summary and description of the terminology used in India surrounding mental health and wellbeing.

| **Word / term** | **Refers to** |
| --- | --- |
| Tension | Feelings of stress and anxiety. |
| Emotional wellbeing | Used to describe a state of happiness, coping well and things being under control. |
| Depression | No equivalent in Kannada language. However, ‘depression’ is commonly used to describe one's non pervasive low mood with higher intensity and duration that sadness. |
| Learning disability | Difficulties in reading and writing, that affect academic performance and doesn’t improve with personal tutoring either. |
| Anxiety | Not often used. |
| Being on track | A young person being stable and in line with adamic ad behavioral expectations for age. |
| Resilience | Managing difficulties. |
| Self-esteem | Having positive opinion about self and being confident. |

Supplementary File 6: ADAPT Step 2 Review findings

**Umbrella review 1: The effectiveness of universal school interventions for prevention or treatment of adolescent anxiety and depression symptoms.**

Following title and abstract screening, 29 systematic reviews progressed to full-text screening, with 18 systematic reviews meeting our inclusion criteria. These 18 reviews contained 23 studies evaluating 15 unique interventions. Each of these interventions were then candidate reviewed, resulting in the exclusion of 8 studies spanning 7 unique interventions. In total, 8 unique interventions, reported across 15 studies, and included in 17 systematic reviews, progressed for in-depth review following the ADAPT steps. A manual search for publications since the most recent eligible systematic review publication date did not identify any further eligible interventions.

**Figure S6.1** **Prisma flow diagram for Review 1**


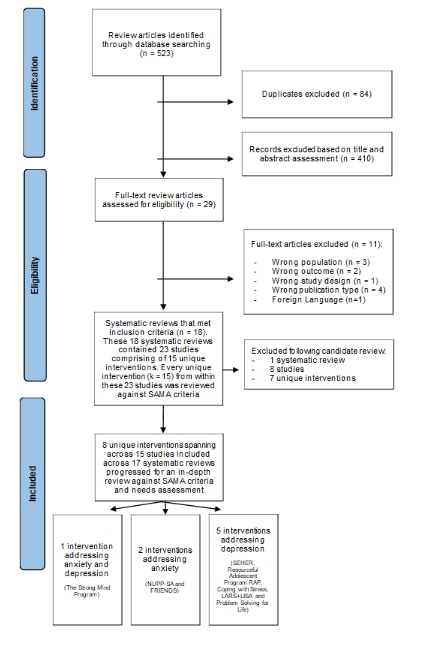


**Table S6.1**  **Overview of the 15 studies covering 8 unique interventions that progressed to ADAPT Step 2**

| **Study** | **Country** | **Prevention Type** | **Focus** | **Setting** | **Age Range** | **Format** | **N** | **Program** | **Program Content** | **Mode of Delivery** | **Number of Sessions** | **Intensity**  **(minutes)** | **Outcome Measures** | **Control** | **LT Follow up(s)** |
| --- | --- | --- | --- | --- | --- | --- | --- | --- | --- | --- | --- | --- | --- | --- | --- |
| Anue and Stilesl (2009) | HIC | Universal | Anxiety | Secondary School-based | 10-15 | F2F | 1633 | NUPP-SA | CBT | Psychologists | 3 | 135 | SPAI-C, SCARED, SMFQ, SDQ | NI | Baseline, post-intervention |
| Burckhardt et al (2016) | HIC | Universal | Anxiety + Depression | Secondary School-based | 15-18 | F2F | 267 | The Strong Minds Program | 3^RD^ | Psychologists | 16 | 480 | FS, DASS-21 | UC | Baseline, post-intervention |
| Horowitz et al (2007) | HIC | Universal | Depression | Secondary School-based | 14-15 | F2F | 380 | Coping with Stress | IPT | Clinical psychology graduate students | 8 | 720 | CDI, CES-D | UC | Baseline, post-intervention, 6-month |
| Lock and Barret (2003) | HIC | Universal | Anxiety | Secondary School-based | 9-16 | F2F | 977 | FRIENDS | CBT | Teachers | 10 | 750 | RCMAS, SCAS, CDI, CSCY, ADIS-C-IV | NI | Baseline, post-intervention, 12-month |
| Merry et al (2004) | HIC | Universal | Depression | Secondary School-based | 13-15 | F2F | 392 | Resourceful Adolescent Program (RAP) | CBT+IPT | Teachers (trained school staff) | 11 | 660 | BDI-II, RADS | AC | Baseline, post-intervention, 6-month, 12-month, 18-month |
| Possel et al (2004) | HIC | Universal | Depression | Secondary School-based | 13-14 | F2F | 324 | LARS+LISA | CBT | Psychologists or graduate students | 10 | 900 | CES-D, ATQ, FESU | UC | Baseline, post-intervention, 3-month, 6-month |
| Possel et al (2011) | HIC | Universal | Depression | Secondary School-based | 12-13 | F2F | 301 | LARS+LISA | CBT | Psychologists or graduate students | 10 | 900 | SBB-DES, SDQ | UC | Baseline, post-intervention, 3-month, 6-month, 12-month |
| Possel et al (2013) | HIC | Universal | Depression | Secondary School-based | 14-16 | F2F | 518 | LARS+LISA | AC+CBT | Psychologists or graduate students | 10 | 900 | CDI | MC | Baseline, post-intervention, 4-month, 6-month, 12-month |
| Rivet-Duval et al (2011) | HIC | Universal | Depression | Secondary School-based | 12-16 | F2F | 160 | Resourceful Adolescent Program (RAP) | CBT+IPT | Teachers | 11 | 660 | RADS-2, BHS, HSC, YCI, RSE | WL | Baseline, post-intervention, 6-month |
| Rodgers and Dunsmuir (2015) | HIC | Universal | Anxiety | Secondary School-based | 12-13 | F2F | 62 | FRIENDS | CBT | Psychologists | 10 | 600 | SCAS, CRS | WL | Baseline, post-intervention, 4-month |
| Rose et al (2014) | HIC | Universal | Depression | Secondary School-based | 9-14 | F2F | 210 | Resourceful Adolescent Program (RAP) | CBT+IPT | Psychologists | 11 | 495 | RADS-2, CDI, PSSM, CAIR, MSLSS, DISCAP | WL | Baseline, post-intervention, 14-month |
| Shinde et al (2018) | LIC | Universal | Depression | Secondary School-based | 13-15 | F2F | 15232 | SEHER | Health Promotion | Lay counsellors and teachers | N/A | N/A | BBSCQ, PHQ-9, BVQ, GEMS, IQIS | UC | Baseline, post-intervention, 8-month, 17-month |
| Shochet et al (2001) | HIC | Universal | Depression | Secondary School-based | 13-14 | F2F | 260 | Resourceful Adolescent Program (RAP) | CBT+IPT | Teachers with the assistance of clinically trained postgraduate students | 11 | 495 | CDL, RADS,BHS | UC | Baseline, post-intervention, 10-month |
| Spence et al (2003) | HIC | Universal | Depression | Secondary School-based | 12-14 | F2F | 1500 | Problem Solving for Life | CBT | Teachers | 8 | 380 | BDI, DY, ADIS-C, | UC | Baseline, post-intervention,12-month |
| Stallard et al (2013) | HIC | Universal | Depression | Secondary School-based | 12-16 | F2F | 5030 | Resourceful Adolescent Program (RAP) | CBT+IPT | Facilitator | 9 | 495 | SMFQ, CATS, RCADS | AC | Baseline, post-intervention, 6-month, 12-month |

*Note: HIC = high income country, LIC = low income country, F2F = face-to-face, NUPP-SA = Norwegian Universal Preventative Program for Social Anxiety, CBT = cognitive behavioural therapy, 3^rd^ = third wave, IPT = interpersonal therapy, AC = attention control, SPAI-C = X, SCARED = The screen for Child Anxiety-Related Emotional Disorders, SMFQ = Short Mood and Feeling Questionnaire, SDQ = Strengths and Difficulties Questionnaire, FS = Flourishing scale, DASS-21 = The depression anxiety and stress scale, CDI – Children’s Depression Inventory, CES-D = Centre for Epidemiological Studies Depression Scale, RCMAS = The Revised Children’s Manifest Anxiety Scale, SCAS = The Spence Child Anxiety Scale, CSCY =* Coping Scale for Children and Youth, ADIS-C-IV = Anxiety Disorder Interview Schedule for Children, BDI-II = The Beck Depression Inventory, RADS = Reynolds Adolescent Depression Scale, ATQ = Automatic Thoughts Questionnaire, FESU = The questionnaire of social support, SBB-DES = The Self-Report Questionnaire – Depression, RADS-2 = Reynolds Adolescent Depression Scale-2, BHS = The Becks Hopelessness Scale, HSC = The Hopelessness Scale for Children, YCI = Youth Coping Index, RSE = Rosenberg Self-Esteem Scale, CRS = Child Rating Scale, PSSM = Psychological Sense of School Membership, CAIR = Clinical Assessment of Interpersonal Relations, MSLSS = Multidimensional Students’ Life Satisfaction Scale, DISCAP = X, BBSCQ = Beyond Blue School Climate Questionnaire, PHQ-9 = Patient Health Questionnaire, BVQ = Bullying Victimisation Questionnaire, GEMS = Gender Equitable Men Survey, IQIS = WHO’s Illustrative Questionnaire for Interview-Survey, BDI = Becks Depression Inventory, DY = Dysthymia, ADIS-C = s. The Anxiety Disorders Interview Schedule for Children, CATS = Children’s Automatic Thoughts Scale, RCADS = Revised Child Anxiety and Depression Scale. NI = No intervention, UC = Usual curriculum, MC = Multiple controls, WL = waitlist.

**Review 2:** Ef**fectiveness of interventions for improving secondary school teachers’ mental health literacy**

Following title and abstract screening, three systematic reviews progressed to full-text screening, with all three systematic reviews meeting our inclusion criteria. These three reviews contained 20 studies evaluating nine unique interventions. Each of these interventions were then candidate reviewed, resulting in the exclusion of 15 studies spanning 5 unique interventions. In total, three unique interventions, reported across five studies, and included in three systematic reviews, progressed for in-depth review following the ADAPT steps. A manual search was conducted for studies published since the most recent eligible systematic review publication date. However, this search did not identify any new eligible interventions.

**Figure S6.2 Prisma flow diagram for Review 2**


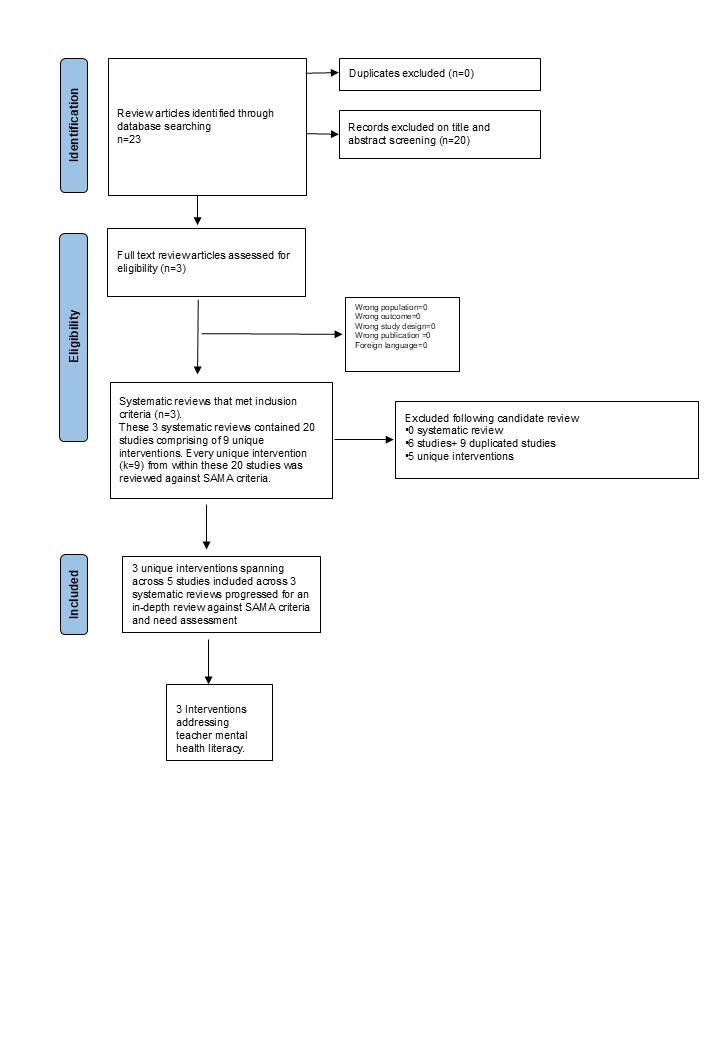


**Table S6.2** **Overview of the 5 studies covering 8 unique interventions that progressed to ADAPT Step 2.**

| **Author** | **Target** | **Setting** | **Program** | **No. Sessions** | **Instruments** |
| --- | --- | --- | --- | --- | --- |
| Eustache et al. (2017) | Promoting student mental health | Secondary school | Brief mental health training program | 2.5 d | Mental health knowledge and attitude assessment which was not standardised |
| Kidger et al. (2016) | Mental Health First Aid (MHFA) youth MHFA training | Secondary school | WISE (Wellbeing in Secondary Education) | 2d | Staff and student questionnaires, Warwick Edinburgh Wellbeing Scale (WEMWBS), PHQ-9, self-report Strengths and Difficulties Questionnaire (SDQ) |
| Kutcher et al. (2015) | Enhancing mental health literacy for educators | Primary; Secondary school | The African Guide: Canadian school mental health curriculum resource Malawi version | 3d | 30-item questionnaire to measure knowledge and attitude towards mental health and mental disorders (Cronbach’s α = 0.63). 8 items to measure attitude (Cronbach’s α = 0.54) |
| Kutcher et al. (2016) | Mental health literacy for secondary school teachers | Secondary school | The African Guide African Guide culturally adapted from a Canadian T*he Guide*) | 3d | 30-item questionnaire to measure knowledge and attitude towards mental health and mental disorders. |
| Wei and Kutcher (2014) | Help educators identify youth with mental disorders and link them to appropriate services | secondary school | ‘Go-To’ Educator Training | 1d+ 0.5d (trainer session) | Anonymous Questionnaire with 30 knowledge questions and eight questions regarding attitudes toward mental illness. |

**Review 3: Effectiveness of interventions for improving school climate in secondary schools**

Following title and abstract screening, six systematic reviews progressed to full-text screening, with five systematic reviews meeting our inclusion criteria. These three reviews contained 49 studies evaluating seven unique interventions. Each of these interventions were then candidate reviewed, resulting in the exclusion of 49 studies spanning 7 unique interventions. None of these studies and interventions progressed for in-depth review. A manual search was conducted for studies published since the most recent eligible systematic review publication date. This returned two interventions: SEHER (Shinde et al., 2018) and the Canadian Guide (Go-to-Educator Training, Milin et al., 2016).

**Figure S6.3 Prisma flow diagram for Review 3**


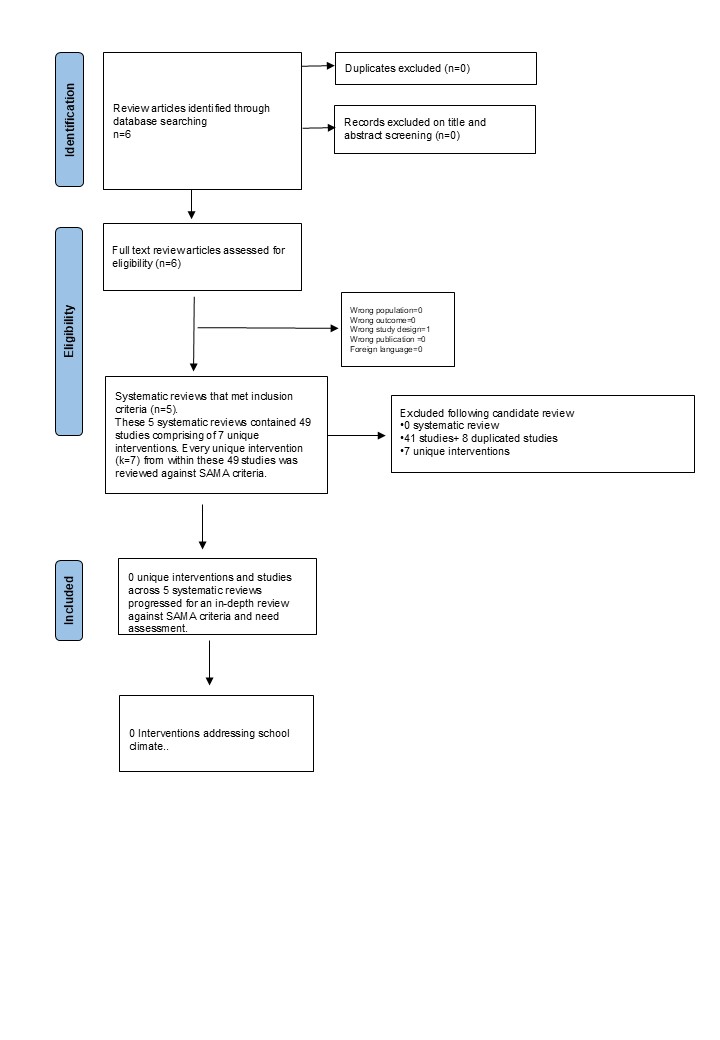


**Table S6.3** **Overview of the 7 studies covering 8 unique interventions that progressed to ADAPT Step 2.**

| **Author** | **Country** | **Target** | **Setting** | **Age** | **Program** | **No. Sessions** | **Control** | **Intensity** | **Instruments** |
| --- | --- | --- | --- | --- | --- | --- | --- | --- | --- |
| Economou et al, 2011 | HI | Anti-stigma towards Schizophrenia | School | 13-15 | Anti stigma programme run by University Mental Health Research Institute | 1 | Nutrition and healthy living | 120 | Beliefs and attitudes measured by Alberta Pilot Site Questionnaire Toolkit (Thompson et al., 2002), |
| Chan et, 2009 | HI | Reducing stigma towards schizophrenia (attitude, social distance and knowledge) and the intervention condition effect | School | 13-18 | ‘‘The Same or Not the Same.’’ | 1 | No control group | 35 | Measures related to stigmatizing attitudes (Public Stigma Scale), social distance (Social Distance Scale), knowledge about schizophrenia (Knowledge Test) and familiarity with schizophrenia (Level of Contact Report) |
| Aseltine et al, 2004 | HI | 1) Self-reported suicide attempts & suicidal ideation (2) knowledge & attitudes about depression & suicide, (3) help-seeking behaviour | High School | 14 -18 | Signs of Suicide (SOS) | Stretched for 2 days | UC |  | Questionnaire measuring: Self- reported suicide attempts & ideation, Knowledge & attitude about depression & suicide, Help seeking behaviour adapted from different scales. |
| Strunk et al, 2014 | HI | Understanding the risk for suicide in teens and helping themselves in the present or in the future when dealing with depression or suicide ideation | High School | 13-18 | Surviving the Teens | 4 | WL | 50 | A survey instrument was developed and validated |
| Pejovic et al, 2009 | UMI | Overcoming stigma-related problems, attitudes of students towards their peers with mental health problems, acceptance active support and help | High school | 15 | Anti-stigma program | 6 | No control | 60 | Opinion about Mental Illness Questionnaire (OMI) |
| Swartz et al, 2010 | HI | Improving high school students’ knowledge about depression. | High school | 14-15 | Adolescent depression awareness program (ADAP) | 2-3 | NI | 180 | Adolescent Depression Knowledge Questionnaire (Says, its available from the authors) |
| Karcher et al., 2009 | HI | connectedness, self-esteem & attachment | School | 15 - 17 | Cross - age peer mentoring program |  | NI | 8 hrs | The Hemingway: Measure of Adolescent Connectedness, Self-Esteem Questionnaire, Inventory of Parent and Peer Attachment  (IPPA). |

**Review 4: Effectiveness of interventions for improving parent mental health literacy**

Following title and abstract screening, two systematic reviews progressed to full-text screening, with one systematic review meeting our inclusion criteria. This one review contained four studies evaluating four unique interventions. Each of these interventions were then candidate reviewed, resulting in the exclusion of one study spanning one unique intervention. In total, three unique interventions, reported across three studies, and included in one systematic review, progressed for in-depth review following the ADAPT steps. A manual search was conducted for studies published since the most recent eligible systematic review publication date. This search returned one intervention (Morgan et al, 2019) - Youth Mental Health First Aid Course delivered to parents.

**Figure S6.4 Prisma flow diagram for Review 4.**


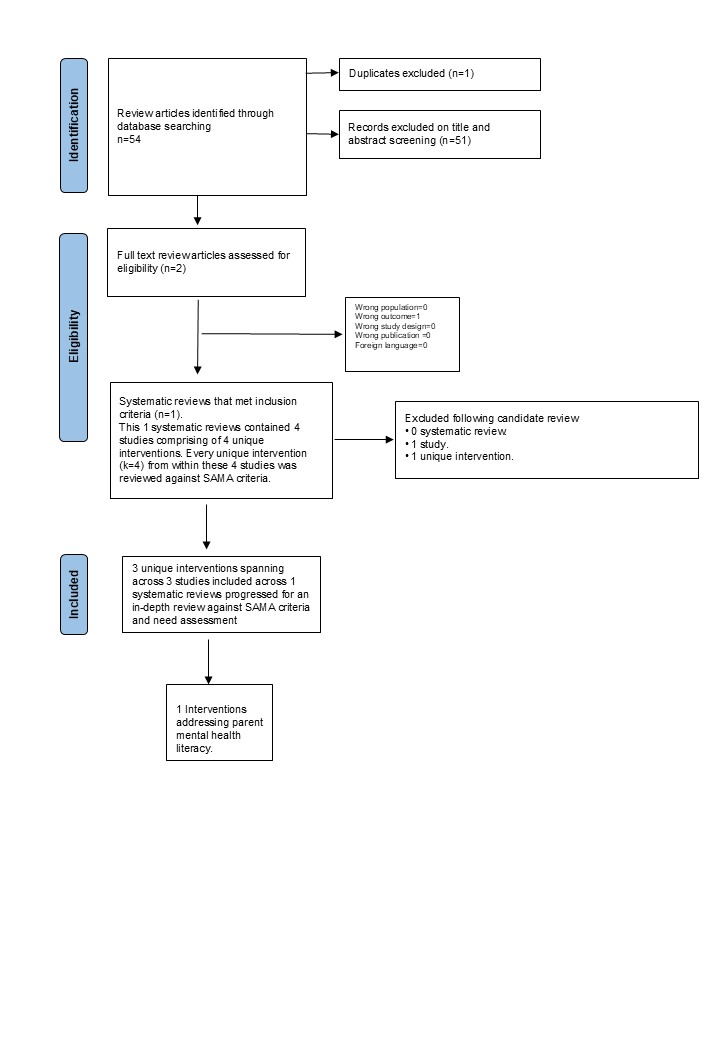


**Table S6:4**  **Overview of the 3 studies covering 8 unique interventions that progressed to ADAPT Step 2.**

| **Author** | **Target** | **Setting** | **Age** | **Program** | **No. Sessions** | **Mode of delivery** | **Intensity** | **Instruments** |
| --- | --- | --- | --- | --- | --- | --- | --- | --- |
| Deitz et al. (2009) | a) knowledge of mental health issues (ability to detect signs of depression and anxiety), (b) self-efficacy in handling mental health issues in youth, (c) better family cohesion, adaptability, and positive communication; and (d) more positive attitudes toward seeking mental health services. | Through the workplace | 42 (mean age) | Web based program, Youth mental health: A parent's Guide (YMH) | To complete watching by two weeks | Multimedia Web-based- Recorded |  | Family Functioning Measures (FACES III Scales, (Olson, Portner, & Lavee, 1985), Parent Adolescent Communication (Barnes and Olson’s (1982), Knowledge of Childhood Depression and Anxiety, Attitudes about emotional and mental health problems in youth, Treatment seeking professional psychological help and attitudes about emotional and mental health problems in youth, Treatment seeking, self-efficacy and confidence in ability to address mental health issues in children, Reaction to the program. Only first two are validated measures. Rest were adapted for this study. |
| Lopez et al. (2009) | To increase (a) knowledge of psychosis, (b) efficacy beliefs that one can identify psychosis in others, (c) attributions to mental illness, and (d) professional help-seeking. | Community | 42 | LaClave | 1 | Psychoeducation about psychosis | 35 | Open ended questions asked to participants to measure the following: Efficacy belief, Illness attribute, help seeking, acceptability of training. The responses were rated by the researchers. Inter rater agreement was measured |
| Hurley et al. (2018) <https://link.springer.com/article/10.1007/s10826-018-1071-y> | (a) to raise awareness of parents’ role in promoting  and supporting positive adolescent mental health, and (b) to  increase parental mental health literacy. | Community | 44.86 | No Name | 1 | Education | 55-80 | Adapted version of The Anxiety Literacy questionnaire (A-LIT; Gulliver et al. 2012) and the Depression Literacy questionnaire (D-LIT; Griffiths et al. 2004), Mental Health Literacy scale (O’Connor and Casey 2015) The Kessler-6 (K6; Kessler et al. 2002) to measure parent psychological distress, Single item measure: Parental confidence to provide help |

Supplementary File 7: ADAPT Step 3 Intervention Exploration

ADAPT STEP 3 following Review 1: The effectiveness of universal school interventions for prevention or treatment of adolescent anxiety and depression symptoms.  A reporting template was completed for each candidate intervention to enable cross-team discussion of suitability to take forward to ADAPT Step 3. These were compiled on the basis of intervention manuals (where available) and published studies of the interventions.

**1. Interventions targeting anxiety and depression (n=1)**

| **Intervention Name** | The Strong Minds Program |
| --- | --- |
| **Paper Name** | Burckhardt et al. (2016) |
| **Country** | Australia |
| **Institute** | Black Dog Institute |
| **Available** | Not yet publicly available – clarified with Rowan Burckhardt.  They stated they could not let us see more than was in published literature. |
| **Feasibility / Development studies** | <https://capmh.biomedcentral.com/articles/10.1186/s13034-017-0164-5> smaller version. |
| **Aim** | **(a)** to improve subjective well-being for all participants; **(b)** to reduce symptoms of anxiety and depression for those who began the program with elevated symptoms (i.e., treatment of psychopathology); and **(c)** to reduce the likelihood of symptoms emerging in participants who began the program with low levels of anxiety and depression. The current study reports on the first two aims of: improving subjective wellbeing and reducing existing symptoms of psychopathology. |
| **Research Question / Hypothesis** | Does the program lead to reductions in symptoms of anxiety and depression in those with elevated symptoms and does it lead to improved subjective wellbeing for all participants? It was hypothesized greater benefits in anxiety and depression would be shown in indicated group and that all students would show increased in wellbeing. |
| **Intervention** | |
| Developed by | Burckhardt and Manicavasagar (paper authors) |
| Therapeutic approach | Acceptance and Commitment Therapy combined with Positive Psychology.Evidence for the application of ACT with adolescents is sparse but 4 RCTs have found that it can reduce depressive symptoms, unsafe sexual behaviours, chronic pain, anxiety, and stress |
| Logic model / Theory of change | None cited. |
| Content | **ACT component**:  6 areas ‘Values’ is the identification of one's values or the personality qualities one wishes to espouse. ‘Committed action’ is taking action that is consistent with one's values’. Contact with present moment’ is present-moment awareness. ‘Observer-self’ is the notion that thoughts, feelings, and body sensations may pass but the self remains constant. ‘Acceptance of emotions’ is the acceptance rather than avoidance of negative emotions. ‘Thought Defusion’ is where negative thoughts are regarded as incidental internal dialog.**PP component**: derived from a report by two Australian PP experts, which was commissioned for a previous PP project {not referenced in paper}. Experts identified 11 components based on a review of the literature that they considered to be important for subjective wellbeing: mindfulness, gratitude, kindness, character strengths, flow, optimism, hope, meaning, social relationships, healthy lifestyles, and goals. Four components were selected for the intervention meaning, kindness, social relationships, and healthy lifestyle (exercise and managing stress) - based on (a) an additional review of the research indicated that they were important for subjective wellbeing; (b) they did not contradict the message of ACT; and (c) they were considered to be relevant to young people. Mindfulness was already largely covered by the ACT component. |
| Sessions | *Table 1 Overview of the Strong Minds Program:*  \| Total contact time = 8 hours (16, 30-minute sessions delivered over 3 months) \| \| \| \| --- \| --- \| --- \| \| ACT Based \| Session 1 \| Introduction to the program; myths of happiness; introduction to values \| \| ACT Based \| Session 2 \| Values \| \| ACT Based \| Session 3 \| Committed action \| \| ACT Based \| Session 4 \| Utility of emotions and the mind as a problem-solving machine \| \| ACT Based \| Session 5 \| Emotional avoidance and acceptance \| \| ACT Based \| Session 6 \| Thought fusion and diffusion \| \| ACT Based \| Session 7 \| Contact with present moment \| \| ACT Based \| Session 8 \| Contact with present moment and observer self \| \| PP Based \| Session 9 \| Applying all ACT components using the example of bullying \| \| PP Based \| Session 10 \| Assertiveness part 1 \| \| PP Based \| Session 11 \| Assertiveness part 2 \| \| PP Based \| Session 12 \| Kindness \| \| PP Based \| Session 13 \| Introduction to wellbeing; being human; meaning; managing stress \| \| PP Based \| Session 14 \| Relationship between money and happiness; and physical exercise \| \| PP Based \| Session 15 \| Social relationships \| \| PP Based \| Session 16 \| Final session – wrap up and students sharing how workshops have helped \| \|  \|  \|  \| |
| Delivery | During the timeslot allocated to pastoral care classes. |
| Dosage | 8h (16 x 30-minute sessions) twice per week. |
| Duration | 3 months. |
| Delivery agent | External psychologist (lead author Burckhardt) assisted by RA. |
| Delivery Strategies / tools | Verbal explanations, metaphors, personal examples, videos, experiential exercises, and images, ppt slides. Each lesson did not follow a rigid format. Rather, during each lesson the facilitator aimed to ensure that the concepts were explained and that students were instructed in how they could be used. Due to the large group size, workshops were delivered in a lecture-style manner. |
| **Control condition** | Usual ‘Pastoral Care’ classes by school staff on facing challenges in their lives such as social justice, managing social media and online harassment, substance use and volunteer work. |
| **Randomisation** | Cluster randomization was conducted, with tutorial groups (8 in each Year) being randomized to receive either the intervention or control condition. |
| **Fidelity checks** | Independent clinical psychologist experienced in ACT scored the audiotaped sessions using an adherence scale specifically developed for this study but based on previous ACT fidelity measures. |
| **Outcome measures** | Depression anxiety and stress scale—short form (DASS-21) [only conducted analysis on sub-groups who scored high on this at baseline n=63 intervention group n= 55 control]Flourishing scale (of subjective well-being) |
| **Findings** | Main effects: When Y10 + Y11 were examined together, significant reduction in int group of depression, stress, and DASS-Total scores. d = large for stress and medium for depression and DASS-Total scores. Improvements were clinically meaningful.Effect on WB for Y10 only. May be related to the increasing responsibility and concerns in Y11. |
| **Moderators / mediators** | None measured. |
| **Author comments** | The observed changes in the present study are similar in magnitude to the changes observed in other early intervention programs utilizing CBT. e.g., Stice et al. (2009) found a medium effect size across CBT early intervention for high-risk adolescents and in Neil and Christensen (2007) SR. Use of an active control may have led to smaller d.There is merit in teaching young people skills in the emotion regulation technique of acceptance. Meta-cognitive ability increases with age. Thus, non-cognitive approaches may be more effective with younger populations compared to adults. |
| **Limitations** | Small, indicated group (n= 63). No blinding of participants.Students may have missed particular workshops.Design of the study did not allow differentiation of the effects of the ACT content from the PP component. Reasons for dropping out were not systematically collected. |
| **Team review** | Good contender as not CBT based. But no effects on older group's WB is a limitation. May meet in-country needs assessment for work which is values based. Unsure if it is possible for lay workers to deliver although authors advocate a train the trainer’s approach and in feasibility study teachers delivered some sessions. Strong Minds may have secured good effects as was delivered by an ACT expert. We could consider delivering just the ACT component and not the Positive Psychology component, but study did not identify active ingredients. Intervention in principle amenable to cultural adaptation. |
| **Decision** | Reject. Can not be taken forward as intervention manual not available. However, some elements informed SAMA. |
| **2. Interventions targeting depression (n=5)** | |
| **Intervention name** | SEHER |
| **Paper Name** | Shinde et al (2020) |
| **Country** | India |
| **Institute** | Sangath |
| **Available** | Yes |
| **Feasibility / Development studies** | Shinde et al (2017) - The development and pilot testing of a multicomponent health promotion intervention (SEHER) for secondary schools in Bihar, India. |
| **Aim** | To examine the effects of the intervention after two years of follow up and to evaluate the consistency of the findings observed over time. |
| **Research Question / Hypothesis** | To develop and evaluate the effectiveness and cost-effectiveness of a comprehensive adolescent health promotion intervention delivered by teacher-as-SEHER Mitra (TSM) and SEHER Mitra (SM), compared with the Tarang Adolescence Education Programme implemented by the State Government of Bihar. |
| **Intervention** | |
| Developed by | Developed by Sangath, in partnership with the Directorate of Education, Government of Bihar |
| Therapeutic approach | N/A |
| Logic model / Theory of change | The SEHER multicomponent , whole school intervention was inspired by the Health Promoting Schools Framework and adapted elements from previous work using that framework led by the investigators in Goa and the Gatehouse Project. In brief, the intervention’s conceptual framework emphasises the importance of positive school climate, which was defined as comprising supportive relationships among school community members, a sense of belonging to the school, a participative school environment, and student commitment to academic values. |
| Content | \| **Level** \| **Activities** \| \| --- \| --- \| \| **Whole School** \| Awareness generation \| \| Speak-out box \| \| Wall magazine \| \| Competitions \| \| School Health Promotion Committee \| \| Healthy school policies \| \| **Group** \| Peer groups for grades 9 and 10 students \| \| Workshops for students and teachers \| \| **Individual** \| Individual counselling and referral \| |
| Sessions | The SEHER intervention addresses the following concerns of adolescents:Health and hygieneBullyingSubstance useGender and violenceMental health*****Reproductive and sexual healthEffective study skillsRights and responsibilities*****For the specific “Mental Health component” subtopics included:Different emotions and their effectsRecognising signs of anger and sadnessEffects of anger and sadnessDealing with anger and sadnessStress and its effectDealing with stressWhat is mental health? Building positive mental healthWhat is depression, suicide, anxiety, and its symptoms?How to handle depression, suicide, and anxiety.These subtopics were covered using various formats and levels within the schools including: assemblies, wall magazines, classroom sessions, workshops etc and could be delivered in the format of a lecture, display, discussion, quiz, activities or case vignettes. |
| Delivery | Multiple delivery slots due to being a whole school approach with multiple components working collectively. |
| Dosage | Whole school approach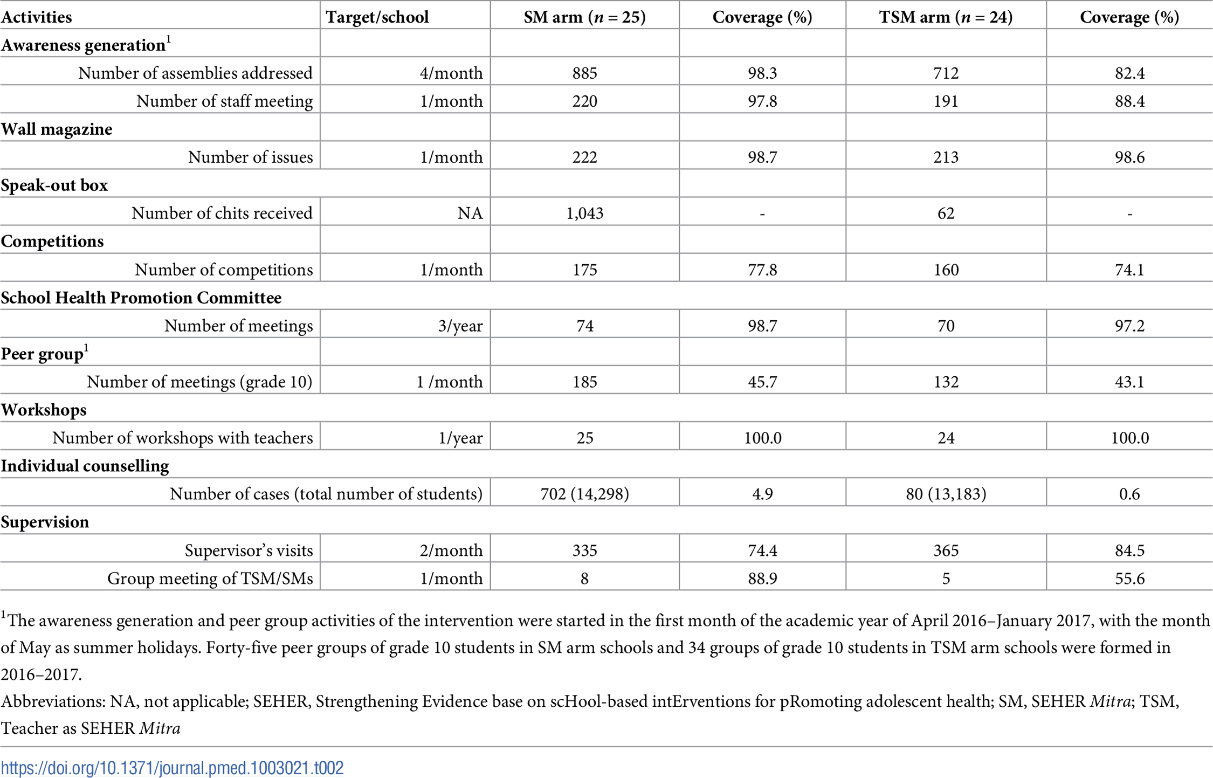 |
| Duration | 2 years |
| Delivery agent | Delivery leads were lay counsellors and teachers. Both delivery leads completed 7 days residential training and 3 days of refresher training. Supervision session were also provided.**Lay counsellors** = SEHER Mitra (SM)– Appointed by Sangath. Above 20 years of age. Minimum bachelor’s degree completed. Good communication and facilitation skills. Fluent in the local language (Hinda).**Teachers** = SEHER Mitra (TSM) - Nominated by the school. Minimum experience of 5 years of teaching in secondary schools. Minimum of 15 years of service remaining. Not teaching TARANG-AEP curriculum.There was 1 SM or one TSM per school. |
| Delivery Strategies / tools | Appointment of 1 counsellor for a cluster of 2/3 schools.Training of all teachers and dividing the components among them for implementation.Orientation of 2-3 teachers per school who can jointly deliver intervention activities.Linking RKSK counsellors to schools. |
| **Control condition** | A standardised classroom-based life skills Adolescent Education Program (AEP). |
| **Randomisation:** | Three arm cluster randomisation (1:1:1) comprising of 74 schools in the trial. As the intervention was implemented at the whole-school level, the school was considered as the unit of randomisation.Of the 136 government-run secondary and higher secondary schools in the Nalanda district of Bihar, 112 were eligible for inclusion in the trial based on three criteria:currently implementing AEP.>100 students in grade 9. >4 employed teachers.Of the 112 eligible schools, 75 were randomly selected to participate in the trial. To ensure that the selected schools were representative of larger government secondary schools in the district, 68% of coeducational (63/93), 69% of girls-only (9/13), and 50% of boys-only (3/6) schools were selected. The schools were allocated in a 1:1:1 ratio using minimisation, balancing on:***Type of school*** (only secondary or combined secondary and higher secondary school).**School size** (101–300, 301–600, or >600 students).**Gender composition** (coeducational, boys only, or girls only).The schools were randomly assigned to the three interventions (government-run AEP, government-run AEP plus SEHER intervention delivered by SM, or government-run AEP plus SEHER delivered by TSM). An independent statistician carried out the random allocation. |
| **Fidelity checks** | Implementation indicators were obtained from monthly logs and counselling case records maintained by the SMs and TSMs, the field visit reports by supervisors, and students’ self-reported coverage of intervention activities collected at the 17-months assessment. These indicators mainly included coverage of each component of the intervention, the quality of intervention implementation, and the students’ self-reported exposure to the intervention. |
| **Outcome measures** | School Climate = Adapted version of BeyondBlue School Climate (BBSCQ)Experience of bullying = Bullying Victimization QuestionnaireViolence = Global school-based student Health SurveyAttitude towards gender equality = Gender Equitable Men Survey (GEMS)Knowledge and attitude towards reproductive and sexual health = Adapted version of WHO’s Illustrative questionnaire for interview-surveys with young peopleDepressive symptoms = Patient Health Questionnaire-9 |
| **Findings** | The SEHER intervention delivered by **lay counsellors** was superior to both the control and **teacher** delivered arms on the primary outcome, and most secondary outcomes, in both cohorts. The lay counsellor-delivered intervention resulted in large improvements in school climate, bullying, violence and **depression** and moderate effects on attitudes towards gender equity.The teacher-led intervention showed no effects on the primary outcome, or most of the secondary outcomes, in either cohort.Year 1: moderate to large effects of the lay counsellor–delivered intervention on school climate and most health-related secondary outcomes, including DEPRESSION (depression: aMD=−4.64;95%CI:−5.83–3.45;p<0.001) but no effects of the teacher delivered intervention.Year 2: sustained effects but larger effect sizes on most outcomes compared with one year of the lay counsellor intervention. No effect of the teacher-delivered intervention on any outcome |
| **Moderators / mediators** | None measured. |
| **Author comments** | Recommendations:Appointment of one counsellor for a cluster of 2-3 schools.Training of all teachers and dividing the components among them for implementation.Orientation of 2-3 teachers per school who can jointly deliver intervention activities.Linking RKSK counsellors to schools. |
| **Limitations:** | Outcome measures based on self-report.27% of baseline survey respondents did not complete the 17-month follow up survey. |
| **Team Review** | \| **PROS** \| **CONS** \| \| --- \| --- \| \| Multi-component, whole school, strong evidence base for in-country effectiveness \| Not yet clear on clinical basis for content on depression \| \| Whole school, class and individual components \| Not yet clear on whether includes any self-help \| \| Outcomes relevant to SAMA, including school climate \| Active ingredients not identified \| \| Impact on depression scores captured – could be augmented to include anxiety / stress \| Focus on problem-solving unclear \| \| Broad topic range – builds YP leadership and self-esteem with impact on school climate \|  \| \| Easy scalability (lay counsellor model, fully costed) \|  \| |
| **Decision** | Propose |

| **Intervention name** | Resourceful Adolescent Program (RAP-A) |
| --- | --- |
| **Paper(s) Name** | Shochet er al (2001); Rivet-Duval et al (2011); Merry et al (2003); Stallard et al (2013); Rose et al (2014) |
| **Country** | Program was developed in Australia and has been implemented in the flowing countries: Australia; Mauritius; New Zealand; UK |
| **Institute** | Queensland University of Technology (QUT) |
| **Available** | Resources available to order for a cost, however materials will only be released to individuals who have completed approved training and received an accreditation number |
| **Feasibility / Development studies** | Shochet & Ham (2004) Universal School-based Approaches to Preventing Adolescent Depression: Past Findings and Future Directions of the Resourceful Adolescent Program. International Journal of Mental Health Promotion, 6(3), 17-25. |
| **Aim** | To prevent adolescent symptoms of depression and hopelessness in the school setting. RAP-A incorporates cognitive behaviour therapy and interpersonal psychotherapy principles and teaches adolescents how to recognize and affirm their strengths, increases self-esteem, stress-management skills, cognitive restructuring of negative thinking styles, conflict resolution with parents, and problem-solving skills. |
| **Research Question / Hypothesis** | Shochet er al (2001)- To determine, in the first instance, whether it was feasible to effectively recruit and keep adolescents in a universal school-based program and whether the intervention would achieve adequate program fidelity in the classroom context. Next, we needed to assess **(a)** whether such a program (RAP-A) would be effective in reducing and preventing depressive symptoms and **(b)** whether the adolescents would approve of the program and consider it beneficial.Rivet-Duval et al (2011)- to partially replicate the study by Shochet et al. (2001) in Mauritian school settings. They hypothesized that RAP-A would be significantly better than a waitlist control condition in preventing symptoms of depression and hopelessness, and increasing self-esteem and coping skills, at postintervention and at a 6-month follow-up. The assessment of aspects of positive mental health was important in the light of our aim to increase psychological resilience in young people, which included the promotion of positive coping strategies and maintenance of self-esteem in the face of a variety of stressors.Merry et al (2003)- Our hypotheses were that **(a)** the intervention program called **RAP-Kiwi** would reduce depressive symptoms immediately after delivery compared with an active placebo; **(b)** the effect would persist for 12 months; and **(c)** the program would be equally effective for Pakeha and Maori people.Stallard et al (2013)- The aims of this study are to investigate the clinical effectiveness and cost-effectiveness of classroom-based CBT in reducing symptoms of depression in high-risk adolescents compared with a school's usual Personal, Social and Health Education (PSHE) curriculum and an attention control PSHE group.The objectives of this project were to address the following research questions:**(a)** Is classroom-based CBT effective in reducing symptoms of depression in high-risk adolescents (aged 12–16 years) 12 months from the baseline assessment compared with a school's usual PSHE curriculum and an attention control PSHE group?**(b)** Does classroom-based CBT improve other aspects of psychological well-being (negative thoughts, self-esteem and anxiety) compared with the control groups at 6 and 12 months?**(c)** Are the effects of classroom-based CBT and the control programmes on depression at 6 and 12 months different in specific groups, that is according to age, year group and sex, or to reports of depression, bullying, self-harm, alcohol, and drug use at the start of the project?**(d)** Is classroom-based CBT cost-effective in terms of changes in depressed mood and health-related quality of life over a 12-month period?**(e)** How successful was the process of implementing classroom-based CBT in schools, that is how many people took part, how many completed the programme and did service recipients and providers believe that it was acceptable and sustainable?Rose et al (2014)- evaluated the effectiveness of a friendship-building skills program—the Peer Interpersonal Relatedness (PIR) program—in producing larger effects when used in conjunction with RAP. |
| **Intervention** | |
| Developed by | Shochet, Holland & Whitefield, (1997). |
| Therapeutic approach | The interpersonal component stresses the importance of promoting harmony and dealing with conflict and role disputes by developing an understanding of the perspective of others. The common thread that runs through the program is the teaching of techniques to maintain self-esteem in the face of a variety of stressors. |
| Logic model / Theory of change | N/A. |
| Content | Sessions 1 through to 7 follow cognitive–behavioural therapy (CBT) approaches.Sessions 8 to 10 address interpersonal risk and protective factors in adolescent development.In the interpersonal components of the RAP program, groups discuss role transitions during adolescence (such as moving towards greater independence while maintaining positive relations with parents) and encourage use of skills to promote harmony, avoid escalation of conflict, and find ways of repairing conflictual situations. Interpersonal components also cover the skills of perspective taking, that is, the ability to understand another person’s perspective, and skills to broaden social support. |
| Sessions | *Table 2 Overview of the RAP-A*  \| Total contact time = ~8.25 hours (11, 40/50-minute sessions delivered weekly over 3 months) \| \| \| \| \| --- \| --- \| --- \| --- \| \|  \| **Session** \| **Key Message** \| **Goal** \| \| Session 1 \| Getting to know you \| We're interested in you! Let's work together as a team. \| Establish rapport and build trust between group members and group facilitator. \| \| Session 2 \| Building self-esteem \| I'm OK. I'm building on my strengths. \| Introduction to program elements and concepts of self-esteem and personal strengths explored. \| \| Session 3 \| Introduction to the RAP model \| Our body clues and our self-talk affect the way we feel and behave. \| Links between behaviour, body clues, self-talk and emotions are explored, concepts of ‘risky’ and ‘resourceful’ responses are introduced. \| \| Session 4 \| Keeping calm \| Be a detective. Find your body clues and keep calm. \| Detailed exploration of body signals related to positive and negative feelings, strategies to relax and manage stress and anger are explored. \| \| Session 5 \| Self-talk \| I am what I think. \| Exploration of how thoughts affect feelings and behaviour; the skills of cognitive restructuring. \| \| Session 6 \| Thinking resourcefully \| You can change your thinking. \| Continues session 5 with a focus on challenging risky negative thoughts and promoting positive self-talk. \| \| Session 7 \| Finding solutions to problems \| There are solutions to my problems. \| Outlining of a problem-solving model and applying it to interpersonal situations. \| \| Session 8 \| Identifying and accessing support networks \| There is always help at hand. \| Identification and development of a social support network for good times and bad times. \| \| Session 9 \| Considering the other person's perspective \| There are two sides to every story. Take time out, stop and think. \| Developing skills to identify the body clues, self-talk and emotions that the other person might be experiencing in interpersonal situations. \| \| Session 10 \| Keeping the peace and making the peace \| Keep the peace and make the peace. \| Strategies to prevent or manage conflict or to move on from conflict situations. \| \| Session 11 \| Putting it all together \| Being a resourceful adolescent really works! Let's celebrate. \| Review of program content, termination and celebration. \| |
| Delivery Slot | During school class time. |
| Dosage | 8.25 hours (11 X 40/50-minute sessions delivered weekly over 3 months). |
| Duration | 3 months. |
| Delivery agent | Shochet er al (2001)-  Group leaders for the adolescent program were **psychologists** with varied training and experience, ranging from experienced clinicians to **graduate psychology students** on practicum.Rivet-Duval et al (2011)- Program facilitators were eight experienced **teachers** (six females, two males), four from each school. Facilitators attended a 2-day training workshop involving 16 hours of training conducted by one of the research team who was a certified RAP trainer; this trainer also provided ongoing support for the teachers when required. The training workshop involved: 1) information on adolescent mental health, and specifically adolescent depression; 2) theory underlying the program; 3) program content and implementation techniques. To maintain program integrity, one half-day booster training session was organised 6 months following initial training.Merry et al (2003)- The Australian team who developed the program trained the research team who then trained **teachers** from the two schools over 2.5 days.Stallard et al (2013)- delivered by trained facilitators.Rose et al (2014)- Groups were facilitated by **graduate students** (all provisionally registered psychologists) enrolled in doctoral-level clinical training and thus were external to the schools. Psychologists were trained in facilitating  the RAP program from the accredited trainer (http:/www.rap.qut.edu.au/training.jsp).*Note:* Who is eligible to deliver the program?The role of group leader is critical to the effectiveness of the RAP program. Group leaders need to be educational or mental health workers with specific training in the facilitation of RAP-A groups. The following people may be suitable for facilitating the RAP-A program:•Psychologists/ Social workers / Occupational therapists / Psychiatrists / Mental Health Nurses•School counsellors/ Guidance officers/Chaplains•Teachers•Community workersTraining events are offered at regular intervals in Brisbane and Sydney, and throughout Australia on request.  Individual or small group Skype training is available for overseas consumers. |
| Delivery Strategies / tools | A 139-page manual has been designed to provide detailed information about the content of each of the eleven sessions. In addition, the manual describes each of the activities used to demonstrate the concepts being introduced throughout RAP-A. Each session begins by outlining the resource requirements and ends with a copy of the hand-outs. Found in the Participant Workbook.The  RAP-A Workbook comprises all of the activities and information required for the program's individual and group activities. A Participant Workbook is required for each adolescent to write in and keep at the end of the program. (75 pages)Supplementary DVD - With the purchase of a RAP-A or RAP-P Group Leader Manual, a Supplementary DVD is provided. This DVD provides supplementary material to be used in activities throughout the programs. |
| **Control condition** | Shochet er al (2001)-  ‘adolescent watch’ comparison group, which involved the normal school curriculum and three assessments.Rivet-Duval et al (2011)- waitlist control conditionMerry et al (2003)- ‘placebo program’ contained the following sessions: introduction, complete depression rating scales identify activities that are fun, spending time doing collage, use of body language in communications, design a chill-out room, discuss effect of environment on mood, design a family coat of arms, “change your mind” a debate, group activity to solve a problem, art activity, discuss how to give support and help others, community service, use activities to identify differences in beliefs, group decision making in designing a planet, fun activity, fun activity and party, certificate presentation.Stallard et al (2013)- ‘Usual PSHE’ was the usual school PSHE curriculum provided by the school staff and did not involve any external input from the research team.Rose et al (2014)- A placebo program and manual were created that included exercises thought to be therapeutically inactive. Some of these exercises were modelled on those designed by Merry et al. (2004) in their RAP-Kiwi study, although the present program also incorporated relaxation and had a larger emphasis on group discussions of topics relevant to adolescents. Similar to PIR, the placebo program was administered during class time in nine weekly 40 –50 min sessions. The ‘placebo program’ contained the following sessions: getting to know you, relaxation activity, Discussion starting high school, discussion homework, discussion balancing leisure and work, relaxation activity, discussion resisting peer pressure, discussion cyberspace and then a review. |
| **Randomisation:** | Shochet er al (2001)-  no randomisation : Given that this was a universal program being conducted in one school, it was considered important to control for contamination from the active treatment to the comparison group. This was achieved by conducting the program with two different cohorts in the same school (1996, 1997) with the 1996 Year 9 students serving as the control group, and the 1997 students serving as the active intervention groups (RAP–A or RAP–F).Rivet-Duval et al (2011)- Students were randomly assigned at an individual level to either the RAP-A (Intervention) or waitlist (Control) condition within their class groups. Twenty students were assigned to each condition, within each grade (Year 7 and Year 9) and within each school (n = 2), resulting in 80 participants in each condition. The conditions were therefore balanced for grade and gender. Given the nature of the program, it was possible that students would share information about sessions with their peers. Therefore, while all recruitment was conducted at the same time, the Intervention and Control conditions within each school grade were run in different years, with the groups randomly assigned to either 2003 or 2004. Year 7 students in the girls’ school and both year 9 groups completed the intervention in 2003, with the remaining Year 7 boys school group completing the intervention in 2004.Merry et al (2003)- Cluster randomisation occurred resulting in 96 students from school A and 96 students from school B being randomised to the intervention arm and 82 students from school A and 90 students from school B being allocated to the placebo program. Participants were blind to which intervention they received. To check the effectiveness of the blinding, students were asked whether they thought they were in the active or the comparison program at the end of the study. However, the teachers who ran the program were not blind.Stallard et al (2013)- cluster randomised - Year groups were randomly allocated on a 1 : 1 : 1 basis once all schools had been recruited. Balance between trial arms with respect to number of classes, number of students, PSHE frequency and scheduling of PSHE lessons within the school was achieved by calculating an imbalance statistic28 of all possible allocation sequences, restricted to those in which every school had at least one year group allocated to classroom-based CBT. Using a computer random numbers generator, a statistician with no other involvement in the study randomly selected one sequence from a subset of 100 with the most desirable balance properties.Rose et al (2014)- A cluster-randomization trial design was used to assign school classes (n=14 classes) to conditions. Randomization was conducted within schools so that within each school, classes were randomly assigned across the three conditions. |
| **Fidelity checks** | Shochet er al (2001)-  The next question was whether the program would be implemented with sound program fidelity given general classroom and school constraints, such as competing scheduling priorities, unexpected interruptions, discipline problems, space constraints, and so on. The RAP–A program was implemented with good fidelity to the program protocol with an average of 89.3% session accuracy rate. (Time constraints appeared to account for any missed program content.)Rivet-Duval et al (2011)-  none reported.Merry et al (2003)- When running the programs, all teachers completed a weekly integrity checklist and met a research team member weekly to ensure compliance.Stallard et al (2013)- Of the 36 classroom-based CBT sessions observed to assess intervention fidelity, 31 covered all the core tasks, with at least 75% of core tasks being covered in the remaining five sessions. Differences in facilitator ratings of lesson content for classroom-based CBT versus attention control PSHE were examined using one-way analysis of variance and indicated that the classroom-based CBT focused more on self-esteem, emotional awareness and positive thinking, and less on topics traditionally covered in PSHE, i.e. sex education, ethical issues, diversity, religion and citizenship (all p < 0.05). There was no difference between classroom-based CBT and attention control PSHE in facilitators' views of student engagement with lessons.Rose et al (2014)- To ensure intervention integrity, we provided weekly supervision and directly observed 20% of group sessions. A further random 20% of sessions had been tape recorded and were assessed by either Kirsten Rose (1^st^ author) or Caroline J. Hunt (3^rd^ author). No deviations from the manualized programs were observed, and supervision focused on the management of participant behaviours within the group. |
| **Outcome measures** | Shochet er al (2001)-  Three depression measures were administered to provide convergent data on adolescents’ self-report of depressive symptomatology: The Child Depression inventory (CDI), the Reynolds Adolescent Depression Scale (RADS) and the Beck Hopelessness Scale (BHS). When adolescent’s depression scores were above the clinical range in either the CDI or BHS the adolescents concerned were individually assessed. As a result, 15 adolescents were individually interviewed, and 9 were subsequently referred for additional assistance, these 9 participants continued in the universal program but were excluded from data analysis.Rivet-Duval et al (2011)-  Depression measures used: Reynolds Adolescent Depression Scale-2 (RADS-2); The beck Hopelessness Scale (BHS); The Hopelessness Scale for Children (HSC). Coping skills measure used: The Youth Coping Index (YCI). Self-esteem measure used: The Rosenberg Self-Esteem Scale (RSE).Merry et al (2003)- Depression measures used: The Beck Depression Inventory II (BDI-II) and the Reynolds Adolescent Depression Scale (RADS). Students with significant levels of depressive symptoms were referred to the school guidance counsellor for appropriate intervention, they still participated in the program however these students were not included in the analysis.Stallard et al (2013)- Primary outcome: symptoms of depression at 12 months assessed using the SMFQ. Secondary outcomes where the personal failure subscale of the Children's Automatic Thoughts Scale (CATS), Rosenberg Self-Esteem Inventory, Revised Child Anxiety and Depression Scale (RCADS), School Connectedness Scale, Attachment Questionnaire for Children and items relating to bullying (Olweus Bully/Victim Questionnaire), self-harm, cannabis use, use of other ‘street drugs’ (e.g., amphetamines, LSD, ecstasy, cocaine, ketamine, crack, heroin), and alcohol use over the last 6 months.Rose et al (2014)- Measures used: Reynolds Adolescent Depression scale – Second edition (RADS-2), Children’s Depression Inventory (CDI), Psychological Sense of School Membership (PSSM), Clinical Assessment of Interpersonal Relations (CAIR), Multidimensional Students’ Life Satisfaction Scale (MSLSS) and the DISCAP (a semi-structured interview developed for the assessment and diagnosis of diagnostic and statistical manual of mental disorders). |
| **Findings** | Shochet er al (2001)-  Post-intervention=-0.48; 10m FU d=-0.29. active intervention groups had significant reductions in CDI and BHS levels largely through changes from pre- to postintervention and maintenance of these changes at 10-month follow up (resulting in significant shifts from preintervention to follow-up). For the comparison group, there was an initial increase at postintervention. Indicating significant intervention effects for both the RAP intervention groups compared to the comparison group. They also show that the two RAP intervention groups did not differ from one another.Rivet-Duval et al (2011)- Significant int effect at immediate post-int, not sustained at 6 mth follow-up. The intervention group showed lower RADS-2 scores than the control group at postintervention (ES = -0.32) but were similar to the control group at follow-up (ES = -0.02). Controlling for pre-intervention hopelessness, there was a significant main effect at post-intervention, with the intervention group showing lower hopelessness scores than the control group (F(1,157) = 19.6, p < .001, ES = -0.42). This effect was not found at follow-up (F(1,157) = 2.59, ns, ES = -0.08). Controlling for pre-intervention RSE scores, there was a significant main effect at both post-intervention (F(1,157) = 25.0, p < .001, ES = -0.67) and follow-up (F(1,157) = 8.33, p < .01, ES = -0.46). Likewise, controlling for pre-intervention YCI scores, there was a significant main effect at both post-intervention (F(1,157) = 11.0, p < .001, ES = 0.32) and follow-up (F(1,157) = 6.75, p < .01, ES = 0.27).Merry et al (2003)- Significant int effect for BDI-II at immediate post-int. Not sustained at 6, 12, and 18 mth follow-ups.Stallard et al (2013)- Post-intervention no effects.Rose et al (2014)- Significant reduction in depressive symptoms at immediate post-int for RAP-PIR relative to RAP and cont. No significant int effect at 14 mth follow-up |
| **Moderators / mediators** | Shochet er al (2001)-  It was not possible to determine which aspect of the program contributed to change in depression scores. . Although there were differences in the process evaluations of various program components, differences were small and provided no clear evidence regarding the mechanisms of change. One of the key messages from the process evaluations, in spite of being positive, is the degree of variance in individual adolescents’ reactions to different aspects of the program. Given the anonymous nature of the process evaluations, links between program preferences and outcome could not be examined.Rivet-Duval et al (2011)- none measured.Merry et al (2003)- none measured.Stallard et al (2013)- none measured.Rose et al (2014)- The significant relationships found between positive interpersonal relationships, school connectedness, and school satisfaction with lower depressive symptoms is consistent with previous research that has pointed to the importance of peer and school factors in the development of depression in adolescence. Clarifications of those factors that maintain gains and promote long-term improvement are necessary to better understand the usefulness of such interventions. |
| **Author comments** | Shochet er al (2001)-  None.Rivet-Duval et al (2011)- None.Merry et al (2003)- None.Stallard et al (2013)- None.Rose et al (2014)- None. |
| **Limitations** | Shochet er al (2001)-  Only conducted in one school. The RAP versus AW was allocated on the basis of cohort and assessed at different times (although the time span was the same across the two groups). Errors associated with cohort effects.  Reliance on self-report measures of depression.Rivet-Duval et al (2011)-  None of the measures had been previously used or validated in samples from Mauritius. No interviewer-based assessments were used, and there was no direct assessment of intellectual disability or major psychiatric disorders, relying instead on parent and teacher report. Teachers who ran the program distributed the self-report outcome measures, which may also have compromised the validity of the results, and limited resources did not allow the monitoring of treatment. Lastly, the follow-up was limited to 6 months following the intervention and a placebo control group was not included.Merry et al (2003)-  Only a single blind study. Using teachers who have no background of cognitive-behavioural therapy to deliver the program. No independent depression ratings were gathered. Students may have missed sessions.Stallard et al (2013)- Limitations included some sampling biases that need to be considered when generalising from the findings. There were initial difficulties in recruiting schools for the trial, and the participating schools had a greater percentage of white students, were more academically able and had fewer students eligible for free school meals than UK national averages. There was also selective drop-out in those who were slightly older, not living with both parents, using alcohol and cannabis and bullying others at baseline, although multiple imputation of missing data did not have any impact on the main conclusions of the trial. The assessments relied on self-report and no clinical or diagnostic interviews were used, although this was because we were interested in symptoms of and risk for depression rather than identifying established clinical cases of depressive disorder. Young people were not blinded to trial allocation after randomisation as this was not feasible, but assessments were self-completed by young people in classes so the potential for researchers to influence responses was minimal.Rose et al (2014)- The study sample was unselected, and the small effects found may well have been due to the fact that few adolescents were reporting elevated levels of depressive symptoms at the start of treatment, meaning there was little room for further decreases. It must be noted that in the absence of a PIR–placebo condition, it is impossible to tell if the PIR itself or the RAP–PIR interaction was responsible for these effects, and, as such, it is a major limitation of the study. Furthermore, group facilitators were involved in the adolescent assessments, potentially causing the adolescents to report more positively than they might otherwise have done. In relation to sampling, school or classroom effects may have differed had students been recruited from schools drawing from lower socioeconomic status areas, as differences in resources might have an impact on the PIR program. Findings are limited in that they rely solely on adolescent self-report. Finally, as the RAP and PIR programs were implemented by provisionally registered psychologists, the results might have differed had more experienced therapists or school staff been trained to implement the program. |
| **Team review** | It could be a positive that there is a manual and training, despite cost, but scope for co-adaptation is uncertain. Combination of CBT with IPR and adolescent living may be a positive. Lack of long-term effects highlight need to invest in this. Requirement for delivery agents to be specialised is a barrier. |
| **Decision** | Reject |

| **Intervention name** | Problem Solving for Life |
| --- | --- |
| **Paper Name** | Spence et al (2003) |
| **Country** | Australia |
| **Institute** | The University of Queensland, Australia |
| **Available** | The Problem Solving for Life is not available now as it is years old. An adapted version of the materials can be found here, but it is not known how different the program is to the one studied by Spence et al.<https://www.beyondblue.org.au/healthy-places/secondary-schools-and-tertiary/senseability/download-sensibility>*(note: we previously have excluded the program BeyondBlue as there was no significant effect for depressive symptoms across the 3 years of the study or at 24-month follow-up).*From website: All text copyright © Beyond Blue Limited (Beyond Blue), 2010. While Australian school communities are permitted to freely copy SenseAbility materials for communication between teachers, students, parents/guardians and others, the materials must not be modified, altered or adapted, or copied for use in any profit-making activity. While every care has been taken in preparing these materials, neither Beyond Blue nor the authors accept any liability for any injury, loss or damage arising from the use of, or reliance upon the content of, SenseAbility materials. Australian schools must ensure that teachers only use this and other SenseAbility materials in a manner that is appropriate for their target audience. |
| **Feasibility / Development studies** | Spence et al – is an evaluation of the effectiveness of the PSFL program. |
| **Aim** | The prevention of adolescent depression. |
| **Research Question / Hypothesis** | To evaluate the long-term impact of the universal teacher-implemented, classroom based PSFL program and to determine whether the effects of the program on depression were mediated by changes in problem-solving skills. |
| **Intervention** | |
| Developed by | Susan Spence, Jeanie Sheffield and Caroline Donovan. |
| Therapeutic approach | The program integrates two components: cognitive re-structuring and problem-solving skills training. The content was developed to be user friendly, relatively culture-free, age appropriate, interesting, and relevant. |
| Logic model / Theory of change | None cited. |
| Content | The PSFL program integrates two components, namely cognitive restructuring and problem-solving skills training (Beck et al., 1979; D’Zurilla & Nezu, 1980).The first component focuses on cognitive style and teaches young people to identify thoughts, feelings, and problem situations and the relationships between these. This phase also teaches cognitive techniques to identify and challenge negative or irrational thoughts that may contribute to the development of negative affect and depressive symptoms.The second component focuses on teaching life problem-solving skills, including the development of positive problem-solving orientation. This aspect makes use of cognitive restructuring methods to develop positive orientation toward problem solving. Teaching methods include didactic sessions; cartoons; individual, small group, and whole class interactive exercises and activities; home-tasks; and diary keeping. |
| Sessions: | Eight sessions. Teachers were provided with curriculum materials to teach life problem-solving skills, positive problem-solving orientation, and optimistic thinking styles. |
| Delivery Slot | Class period |
| Dosage | Eight self-contained sessions, each lasting a class period of 45–50 min, once per week |
| Duration | 8 weeks |
| Delivery agent | **Teachers** (28 teachers across 8 schools) attended training sessions 6 hr duration. Training covered the theory, program content, and implementation. To enhance teacher adherence to program fidelity, they completed a qualitative evaluation form after each session, recording the usefulness, organization, and degree of implementation of that program component. |
| Delivery Strategies / tools | Teaching methods included didactic sessions; cartoons; individual, small-group, and whole-class interactive exercises and activities; home tasks; and diary keeping. Implementation required minimal preparation by teachers, as supporting materials such as resource books, overheads, background notes, handouts, cartoons, puzzle pieces, and posters were provided |
| **Control condition** | Teaching as usual (TAU). |
| **Randomisation** | Schools were matched in pairs on the basis of state versus private funding status and size of enrolment across Grades 8–12. All schools were classified as urban, and all were coeducational, with an approximately equal number of boys and girls. Out of 16 schools, matching on both variables was possible for 7 out of 8 pairs. Schools were randomly assigned from each pair to either the PSFL intervention or the monitoring control (MC) condition. |
| **Fidelity checks** | After each session, teachers recorded whether they completed each component of the program for that session. Records permitted assessment of teacher adherence to the intervention protocol. At the end of the final session, teachers completed a final evaluation of the adequacy of the curriculum as a whole, whether they felt that the program was effective in teaching life problem-solving skills, and whether they would teach the program again. |
| **Outcome measures** | Beck Depression Inventory (BDI)Dysthymia (DY)Structured diagnostic interview with high-risk students (The anxiety disorders interview Schedule for Children)Survival analysis:To track new or continuing episodes of depression during the follow-up period, we interviewed high-risk students at 6- and 12-month follow-ups using the Longitudinal Follow-Up Evaluation (LIFE) - The LIFE interview uses a series of structured prompts to identify episodes of depression over the previous 6 months.Youth self-report form of the child behavioural checklistThe Child and Adolescent Social and Adaptive Functioning ScaleThe Social Problem-Solving Inventory—Revised Short FormThe Children’s attributional style questionnaire – revised.Five items from the self-report of family functioning inventory |
| **Findings** | Positive post-intervention in symptomatic group and to a lesser extent in low-risk group.  Not maintained at 12m FU. From pre- to post intervention, high-risk PSFL students showed significantly greater reductions in depressive symptoms compared to the control group (the mean BDI score moving into the nonclinical range). Significantly more high-risk students in the PSFL group showed not only a clinically significant and reliable improvement in depression in terms of reduction of BDI scores into the nonclinical range in comparison to the MC group but also clinical equivalence with a normative sample. The low-risk PSFL students also showed a small but significant decrease in BDI scores over the intervention period, whereas the low-risk MC group showed a slight increase in depression scores over time. A statistically significant difference between prevention and control grps on depression not maintained at 12m FU. |
| **Moderators / mediators** | None measured |
| **Author comments** | Reasons why effective long-term depression was not identified:  Age of participants may have been too young; effective long-term prevention of depression requires a greater number of sessions, in small-group format, administered by more highly trained mental health professionals, with a greater level of training in the CBT techniques of the program. |
| **Limitations** | Age of participants: preventive interventions may be more effective if introduced in mid- rather than early adolescence. The higher prevalence of depression in later adolescence may also increase the probability of detecting differences in depression levels across experimental conditions.Impact of number of dropouts.generalizability of the results to schools in general, given that participating schools volunteered to take part in the research. It is possible that staff in the participating schools may have been more enthusiasticteacher adherence to program implementation and student participation levels. It was beyond the scope and resources of their study to obtain detailed observational data relating to teacher adherence to protocols in the administration of PSFL or student participation levels (attendance and homework completion). This assessment was limited to teacher self-report.Failure to use interviewers who were blind to experimental conditions.The young person as the sole informant.The reliability and validity of the LIFE interview in providing retrospective reports of clinical depression over the follow-up period must also be questioned. |
| **Suitability for SAMA** | Problem-solving approaches well endorsed in India. Concern about no long-term effects. Major concern about teacher burden and accessing old intervention materials |
| **Decision** | Reject |
|  | |
| **Intervention name** | LARS + LISA |
| **Paper Name** | Possel et al (2004, 2011, 2013) |
| **Country** | Germany |
| **Institute** | University of Louisville |
| **Available** | No response |
| **Feasibility / Development studies** | Possel et al 2018  – discusses the history and development of the program in depth (20 years of change and consistency) and discusses each session of the program in detail.This program was first developed almost 20 years ago in Germany under the name **LISA** (Leichtigkeit Im Sozialen Alltag; Pössel, Baldus, Horn, Groen, & Hautzinger, 2005) and underwent multiple revisions (LARS&LISA; Lust An Realistischer Sicht & Leichtigkeit Im Sozialen Alltag; Pössel, Horn, Seemann, & Hautzinger, 2004) and cultural adaptations (e.g., USA: **TIM&SARA**, Together Initiating More Socially Advantageous & Realistic Attitudes; Pössel, Martin, Garber, & Hautzinger, 2013) since then.All versions of the program are implemented in gender-homogenous groups. The reason for this is that research indicates that gender homogeneous groups can create spaces where young people, and particularly boys, can share their emotions and feelings without embarrassment (Sukhnandan, Lee & Kelleher, 2000). They are also likely to be less distracted, more open and responsive, and can participate without the fear of compromising their image in front of girls (Warrington & Younger, 2003). Further, the authors report that a pilot study revealed that adolescents worked more effectively and openly as a team if no peer from the other gender was in the room (Pössel, Horn, Seemann, & Hautzinger, 2004). |
| **Aim** | To prevent the increase of adolescent depressive symptoms. |
| **Research Question / Hypothesis** | Possel et al 2004: In addition to analysing depressive symptoms, they studied factors associated with the development of depressive disorders, such as automatic thoughts and social resources. Expected differential effects for subgroups depending on the magnitude of the depressive symptoms: In particular, students without depressive symptoms and students with subsyndromal depressive symptoms should benefit strongly from the prevention program. They will have fewer dysfunctional (i.e., unrealistic, self-devaluating) automatic thoughts and more social skills. Due to improved social skills, students will enlarge their social network and use it more. Participants without depressive symptoms will show no increase in depressive symptoms between the times of measurement, whereas adolescents with subsyndromal depressive symptoms should show a decrease in depressive symptoms after participating in the prevention program. Students with clinical depression will probably not show any effects on proximal or distal objectives because this subgroup would need more intensive assistance than can be offered within this program’s framework to benefit them |
| **Intervention** | |
| Developed by | Originally developed in Germany (Pössel, Horn, Seemann, & Hautzinger, 2004) and was modified for youth in the United States. |
| Therapeutic approach | No – based on the cognitive therapy approach of Beck et al and uses cognitive-behavioural strategies and is informed by Dodge’s social information processing model (Dodge, 1993). |
| Logic model / Theory of change | The LARS&LISA intervention is based on the social information processing (SIP) model (Dodge, 1993) and uses various methods from CBT (Beck, Rush, Shaw, & Emery, 1979). Cognitive and social components of the SIP model are targeted as follows: **(a)** Four cognitive sessions focus on understanding the relations among cognitions, emotions, and behaviors and teach how to identify and challenge negative cognitions; and **(b)** four social sessions train participants in assertiveness and social competence skills (for a description of the links between the SIP and LARS&LISA, see Pössel, Adelson, & Hautzinger, 2011). In addition, the first session outlines the rationale for the program, and the 10th session is a review and celebration. |
| Content | LARS&LISA is a structured program with a manual that provides detailed instructions, examples, and tips for group leaders on how to administer the intervention. The primary materials utilised in the implementation of LARS&LISA are handouts and worksheets, which aid the adolescents in understanding and remembering elements of the program’s content.For example, “Knowledge Checks” are worksheets that the students complete individually at the end of sessions, which are designed to reinforce the concepts that were covered that day. These worksheets simultaneously provide group leaders with feedback about the students’ comprehension of the material. Poster sheets, a whiteboard or blackboard, and a projector can be employed by the group leaders as visual aids to present guidelines, concepts, examples, etc. sweets or popular snacks can be used to create a motivational reward system that encourages participation and observation of the group’s guidelines. Finally, a few specific games and activities require additional materials. For instance, in the initial session, the participants compete in a relay race that requires plastic cups, straws, and small candy-coated chocolates. The LARS&LISA manual can be requested by contacting the authors of the program. |
| Sessions | Each session of the program follows a similar structure and contains a number of common elements. Group leaders begin by presenting the agenda for the day, providing feedback about last session’s Knowledge Check and asking how the students might have used the skills taught in LARS&LISA since the last session. Most of the session is spent on group activities related to one of the five modules (Set Some Goals, Reversible Spiral, Think Tank, Just Do It, and Making Contact). Within these modules, students learn how to generate useful personal goals; understand the interconnected nature of thoughts, feelings, and behaviours; and replace maladaptive thoughts and behaviours with more helpful and realistic alternatives. Group activities may include interactive conversations, the completion of worksheets, or the creation and acting out of role-plays, depending on the session. Knowledge Checks are completed towards the end of most sessions. The group leaders end each session by making connections between that day’s content and the content presented in previous sessions, providing feedback about the observation of guidelines, and giving a preview of the next week’s session.Modifications: LARS&LISA has been modified a number of times to make improvements and accommodate various populations. The program originally contained four main modules, but a fifth was added to increase motivation and make the content more personally relevant to adolescents without depressive symptoms. The program was originally developed in German and was culturally adapted to be implemented in the USA.  \| Session \| Topic \| Objectives \| Content \| \| --- \| --- \| --- \| --- \| \| 1 \| Introductions, Build relationships \| Explain guidelines; create a cooperative atmosphere; provide overview and rationale for topics in the program \| - Get to know each other - Establish basic guidelines: fairness, respect, teamwork, and a positive working atmosphere - Consequences for breaking of guidelines - Introduce program \| \| 2 \| Setting Goals \| Identify and develop goals \| - Define goals - Setting realistic and achievable personal goals \| \| 3 \| Reversible Spiral-1 \| Learn connections among feelings, thoughts, and behaviours; teach concepts of “down” and “up” thoughts \| - Define “feelings,” “thoughts,” and “behaviour” - Reversible Spiral: associations among feelings, thoughts, and behaviours - Introduce “down thoughts” (self-critical, action-blocking) and “up-thoughts” (self-supportive, helpful) \| \| 4 \| Reversible Spiral-2 \| Identify self-critical, action-blocking thoughts \| - Experience the Reversible Spiral - Explore meaning of negative thoughts \| \| 5 \| Think Tank-1 \| Question self-critical, action-blocking thoughts; generate self-supportive, helpful, realistic thoughts \| - Introduce the “reality check” - Create one’s own counter thoughts (i.e., realistic “up-thoughts”) \| \| 6 \| Think Tank-2 \| Learn why self-supportive, realistic thoughts can be important and how to integrate them into one’s life \| - Review meaning of and rationale for “up thoughts” - Identify daily situations in which “up thoughts” can be integrated \| \| 7 \| Just Do it -1 \| Learn differences among assertive, passive, and aggressive behaviours and their consequences. Review connections between thoughts and behaviour \| - Identify signs of assertive, passive, and aggressive behaviour - Discuss pros and cons of different behaviours - Explore associations among negative thoughts; counter thoughts; and assertive, passive, and aggressive behaviours \| \| 8 \| Just Do it -2 \| Practice assertive behaviour Practice not avoiding \| - Demonstrate assertive behaviours - Practice assertive behaviour in role-plays \| \| 9 \| Making Contact-1 \| Learn how to build and maintain friendships \| - Demonstrate verbal and nonverbal strategies to signal interest in others - Role-play “making contact” \| \| 10 \| Making Contact -2 \| Practice building friendships. Obtain feedback about the program \| - Participants evaluate and provide feedback - Hand out certificates of program completion - Good-byes and celebration \| |
| Delivery Slot | classrooms during school hours. |
| Dosage | The program was originally designed to be presented in 10, 90-min weekly sessions. However, the material has been reorganized to accommodate the unique class schedules at various schools that have hosted the program. For example, it has been adapted to be delivered in 16, 60-min weekly sessions for a school that had shorter class periods. |
| Duration | 10 weeks. |
| Delivery agent | The intervention has been successfully implemented by psychologists and supervised students in graduate psychology programs. A study was conducted to evaluate whether the program could be implemented by teachers, but like other similar programs, teachers were found to be less effective as group leaders.Possel et al 2004: Each group was coached by a trainer and a cotrainer. Thus, each school class required a total of four trainers. Trainers were either psychologists (master’s degree equivalent) or graduate students experienced in working with adolescents. Before the training, each trainer went through the program as a participant. During the intervention, all trainers were seen weekly to present video recordings of each session. Recordings were also rated by independent clinicians to ensure that trainers adhered to the manual. |
| Delivery Strategies / tools | In person in a group setting. The ideal number of participants is between 8 and 12, but the program has been implemented with larger (n ≤ 20) and smaller (n = 4) groups. Recommended that two leaders co-lead a group, but groups have also been led by individuals and triads. |
| **Control condition** | Possel et al 2004: Students randomized to the NIC condition remained in their regular Wellness class, which was the same duration as the CB and NSp conditions. Control students participated in the same assessments at each time point as those in the CB and NSp conditions |
| **Randomisation** | Possel et al 2004: tried to recruit both training and control groups in each school; however, there was one school with only one class, which was assigned to the training group. In another school with three classes, randomly assigned two classes to the training group. Separation of each school’s classes with regard to certain conditions was necessary to increase statistical power with constant sample sizes. As a result of the randomization procedure, 200 students (87 girls) in seven classes received the prevention program, and 147 students (79 girls) in five classes received usual lessons. The mean age of the students in the training group was 13.82 (SD = 71). In the control group, the mean age was 14.18 (SD = 78). |
| **Fidelity checks** | To assure adherence to the manual, group leaders participate in a two-step training and while they implement the program in weekly supervision sessions using video recordings of each session. During the first step of the training, future group leaders participated in a mock version of the program, and in the second step, they study the manual, materials, and procedures and discuss their questions with their supervisors. |
| **Outcome measures:** | Possel et al 2004: CES-D, the automatic thoughts questionnaire (ATQ) and the questionnaire of social support (FESU). |
| **Findings** | Possel et al 2004: Adolescents who had participated in prevention program and who reported minimal initial depression scores did not experience significant increases in their scores during the following 6 months. In contrast, adolescents with minimal depression scores who did not participate in the program showed significantly increased depression scores at all three times of measurements.  Indicating LISA-T has a preventive effect on the development of depressive symptoms in the experimental group with minimal depressive symptoms. And reduced depressive symptoms and the number of adolescents in the high-risk group. (author interprets these results as possible intervention effects). Students with clinically relevant symptoms at premeasurement who participated in the program showed no change in depressive symptoms. There was, however, a decrease in symptoms at the 3- and 6-m follow-up when compared with premeasurement for adolescents with clinically relevant symptoms. |
| **Moderators / mediators** | None measured |
| **Author comments** | LISA-T may be an effective training program for adolescents with nonpathological scores and for adolescents with an increased risk of depression. |
| **Limitations** | Student self-reports. Results only allow conclusions about changes in depressive symptoms and not about the prevention of a depressive disorder. |
| **Team review** | Lots of detail and interesting inclusion of social processes but may be too demanding for our age group. Cannot access resource. Training demands are high. |
| **Decision** | Reject |
|  | |
| **Intervention name** | Coping with stress |
| **Paper Name** | Horowitz et al (2014) |
| **Country** | America |
| **Institute** | Kaiser Permanente - https://research.kpchr.org/Research/Research-Areas/Mental-Health/Youth-Depression-Programs |
| **Available** | Yes |
| **Feasibility / Development studies** |  |
| **Aim** | Youth at risk of future depression, but not currently depressed.  Program educates about the nature and risk for depression and how to (a) monitor daily moods; (b) identify activating events; (c) discover, challenge, realistically evaluate, and revise negative beliefs; (d) recognize the connections among activating events, beliefs, and consequences (e.g., affect and behaviours); and (e) problem solve and cope with stressful events. |
| **Research Question / Hypothesis** | Hypothesis 1—Outcome: The cognitive– behavioural and interpersonal therapy prevention programs will be significantly better than the no-intervention control group in preventing depressive symptoms measured at postintervention and at a 6-month follow-up.Hypothesis 2—Moderatorsa. Initial levels of depressive symptoms will moderate the effect of the intervention, such that those with the highest levels of baseline depression will show the greatest benefit ofthe interventions.b. Gender will moderate the effects of the interventions on outcome such that for girls, an interpersonal program will be better than the cognitive– behavioural program, whereas thereverse will be true for boys.c. Personality characteristics will moderate the effect of the interventions on depressive symptoms. The effect of the interpersonal program on depressive symptoms is expected to be stronger for those high versus low in sociotropy, whereas the effect of the cognitive– behavioural program on depressive symptoms is expected to be stronger for those high versus low in achievement orientation.Hypothesis 3—Mediatorsa. The effect of the cognitive– behavioural intervention on change in depressive symptoms will be mediated by a decrease in negative cognitions and an increase in rational and active coping skills.b. The effect of the interpersonal program intervention on change in depressive symptoms will be mediated by improvements in interpersonal relationships. |
| **Intervention** | |
| Developed by | Gregory N. Clarke and Peter M. Lewinsohn - The Coping With Stress (CWS) Course (Clarke & Lewinsohn, 1995) is a psychoeducational, cognitive-behavioral intervention for the prevention of unipolar depression in high school adolescents who have an increased risk of depression (see Beardslee et al., 1993; Muñoz, 1993). It is not meant to be a treatment for active episodes of depression. It is designed to be offered on the school premises, either as a class during regular school hours, as an adjunct to a health class, or as an after-school therapy group or workshop. The target population for this intervention are adolescents who already carry some known increased risk of depression, such as **(a)** having had a past episode of depression; **(b)** reporting persistent sub-diagnostic dysphoria and/or other depressive symptoms (Clarke et al., 1995); **(c)** having depressed parents (Clarke & Hornbrook, in progress); **(d)** being a pregnant, single teen mother; and/or **(e)** having some other known risk factor for depression. |
| Therapeutic approach: | N/A |
| Logic model / Theory of change | The theoretical orientation represented by the multifactorial model of depression proposed by Lewinsohn, Hoberman, Teri and Hautzinger (1985). This depression model is multi-factorial; that is, increased dysphoria/depression is presumed to be the result of multiple etiological elements acting either in concert or in combination, including negative cognitions, stressful events, predisposing vulnerabilities/risk factors (e.g., being female, a previous history of depression, having depressed parents), and immunities to depression (e.g., high self-esteem, coping skills, high frequency of pleasant events and activities). The intervention is based on the hypothesis that teaching individuals new coping mechanisms and strengthening their repertoire of current coping techniques and strategies provides them with some measure of "immunity" against the development of affective disorders, even if they may have several risk factors. In terms of Lewinsohn et al.'s (1985) theory, the aim of the proposed intervention is to supplement these adolescents' immunities to offset their known vulnerability for affective disorder. To take one therapy skill area for example, these adolescents are presumed to be depressed at least in part because they may be predisposed to thinking depressogenic negative/irrational cognitions (Beck et al., 1979). The interventions train these adolescents in cognitive-restructuring skills to permit them to reduce these negative cognitions, and thereby overcome their depression. |
| Content | program educates about the nature and risk for depression and teaches how to **(a)** monitor daily moods; **(b)** identify activating events; **(c)** discover, challenge, realistically evaluate, and revise negative beliefs; **(d)** recognize the connections among activating events, beliefs, and consequences (e.g., affect and behaviors); and **(e)** problem solve and cope with stressful events. |
| Sessions | The sessions consist of active guidance by a group lead and structured activities for participants. A participant workbook, including exercises for practice outside of the group was distributed to all students.  \| Session \| Lesson \| \| --- \| --- \| \| 1 \| Getting to Know each other \| \| 2 \| Coping with Stress \| \| 3 \| Changing your thinking \| \| 4 \| Stressful Situations and Negative Thinking \| \| 5 \| The power of Positive Thinking \| \| 6 \| Changing Negative Thinking to Positive Thinking \| \| 7 \| Irrational Thinking \| \| 8 \| Disputing Irrational Thinking \| \| 9 \| The C-A-B Method \| \| 10 \| Dealing with Activating Events \| \| 11 \| More C-A-B Practice \| \| 12 \| Thought-Stopping Techniques \| \| 13 \| C-A-B in Your Life \| \| 14 \| Planning for Stressful Situations \| \| 15 \| Preventing the ‘Blues’ \| |
| Delivery Slot | After school (Horowitz et al 2014 delivered sessions during student’s regular wellness class period). |
| Dosage | 15 group sessions each 60 mins long that can be offered at a pace of 2-4 times per week (8 X 90 mins sessions were delivered once a week in Horowitz et al 2014). |
| Duration | 2 months |
| Delivery agent | Group leaders were specially trained **school psychologists and counsellors**, each of whom had a minimum of a master’s degree. 40 hours of training. All sessions audiotaped and random set rated for compliance (average protocol adherence was 93.9%). No fidelity data of the program once it was implemented. |
| Delivery Strategies / tools | The program utilized cartoons, role plays, and group discussions. To account for season and school calendar factors, half of all three conditions were run during the fall semester and half were run during the spring semester. Groups were of mixed gender and had between 8 and 15 students, with a median size of 11. Follow-up questionnaires were completed during school time by participants in all three conditions a week after the last groupsession and again 6 months postintervention. Participants who had changed schools were contacted by mail and telephone. Participant safety was monitored throughout the course of the study by senior-level clinicians who supervised the therapists. In addition, school counsellors were informed about the project and were available for consultation and referrals for any student in need of more immediate attention. No serious adverse events occurred during the study. |
| **Control condition:** | Participants in the no-intervention control group attended their regularly scheduled health classes, where they were taught the standard wellness curriculum. Although no materials were provided to create an attention placebo control group, these classes were delivered in groups in a classroom setting similar to that used in the intervention groups. The size, environment, and format of the wellness classes were similar to the interventions, and the primary systematic difference was the content delivered. |
| **Randomisation** | A random number list was used by Jason L. Horowitz to assign participants to the cognitive– behavior program (CB; n = 112), the interpersonal psychotherapy–adolescent skills training (IPT–AST; n = 99), or the assessment-only control condition (n = 169). The control group had more participants because each CB and IPT–AST group was limited to no more than 15 students; in some class periods, the numbers of participants exceeded this constraint, in which case the excess (i.e., over the maximum of 15 students per intervention group) were assigned to the control condition. Within class periods, participants were randomly assigned to condition unless there were fewer than 15 students participating. This occurred for only two classes (25 of 380 participants); for these two classes, randomization was done at the class level rather than at the individual level. |
| **Fidelity checks** | Group leaders were master’s-level clinical psychology graduate students or recent clinical psychology PhDs, all of whom had received prior therapy training. Coleaders were clinical graduate students or undergraduate honors students. To ensure treatment integrity, (a) detailed treatment manuals were used for both CB and IPT–AST, (b) group leaders and coleaders participated in training workshops before beginning the study, and (c) throughout the intervention, weekly supervision meetings were held with clinical experts in the modality (i.e., CB or IPT–AST) leaders provided. During supervision, each session was carefully reviewed, and plans for the next session were outlined on the basis of the manuals. The schools did not permit taping of the group sessions. |
| **Outcome measures** | Depression = Children’s Depression Inventory (CDI) and the Centre of Epidemiological Studies Depression Scale (CES-D). |
| **Findings** | Positive int effect for depressive symptoms measured by the CDI for the CB group at post-int, not sustained at 6 mth follow-up. Both the CB group (Cohen’s d =0.37) and the IPT–AST group (d =0.26) had significantly lower composite depression scores than the control group at postintervention; the CB and IPT–AST groups did not differ significantly from each other Differences between the active intervention groups and controls were largest for individuals with initially high levels of depressive symptoms. Moreover, among those adolescents with elevated levels of baseline depressive symptoms (i.e., high risk), both CB (d = 0.89) and IPT–AST (d = 0.84) had particularly strong effects. Effects not maintained by 6m follow up. |
| **Moderators / mediators** | Moderators*Initial level of depressive symptoms*examined whether CB and IPT-AST groups had a larger effect for individuals who were more symptomatic at baseline. They used a regression model to test for interactions between initial symptoms levels and intervention condition. Baseline CDI scores significantly moderated the effect of both the CB, β =  – 0.12, t(348) = –1.95, p = .05, ΔR2 = .01; and the IPT–AST groups, β = – 0.13, t(348) = 2.05, p = .04, ΔR2 = .01. This interaction indicated that the difference between intervention groups was largest for individuals with initially high levels of depressive symptoms on the CDI.both sociotropy and achievement orientation moderated the effects of the prevention programs on depression at postintervention.*Gender*Controlling for preintervention depressive symptoms, no main effect for gender or gender by treatment condition interactions were detected for either the CES–D or CDI at either posttreatment or the 6-month follow-up.MediatorsAttributional style partially mediated the positive effect of CB, but not IPT–AST, at postintervention. This is in keeping with the content of the CB program, which includes identifying negative thought patterns, looking for alternative explanations for negative events, and challenging the accuracy of negative beliefs. This result is consistent with other studies that have found that changes in attributional style partially mediated the effect of cognitive interventions on depression. Significant effects were not found for the other hypothesised mediators.*Attribution style -* Examination of the relation between intervention condition and attributional style revealed a nonsignificant trend.*Coping -* There was no effect for group at postintervention on coping.*Conflict –* No effect for intervention group on perceived conflict.*Knowledge Checks – after controlling for prior levels of depression, CB quiz scores did not significantly predict post-treatment depression.* |
| **Author comments** | Positive intervention effects, although this likely was due largely to the effect for the high-risk adolescents. That is, those adolescents with the greatest need showed greatest benefit. Result is consistent with the finding that, in general, indicated and selective depression prevention programs produce greater effect sizes than universal programs. |
| **Limitations** | The decrease in depression scores for participants in the two intervention groups in the present study might simply have been a reflection of a demand characteristic rather than a real “treatment” effect. That is, having gone through the intervention, students may have thought that they “should” report fewer symptoms. It wasn’t possible to conduct diagnostic interviews due to the large number of participants. Independent observations of therapists were not completed, so absolute fidelity to intervention protocols cannot be assured. |
| **Team review** | Strong CBT approach to depression. No long-term effects. Maintenance work needed.  No wellbeing component to benefit those with low symptomatology. |
| **Decision** | Reject |
| **3. Interventions targeting anxiety (n=2)****NUPP-SA** (Anue & Stiles, 2009). No response received from authors with regard to intervention content or availability. Reject. | |
| **Intervention name** | Friends for Life |
| **Paper Name** | Rodgers & Dunsmuir 2015 (but participants are aged only 12-13) – Focusing on FRIENDS for Youth Program |
| **Country** | Rodgers & Dunsmuir 2015 – Ireland |
| **Institute** | Rodgers & Dunsmuir 2015 – National Behaviour Support Service, Navan Education Centre, Navan, Ireland |
| **Available** | Available at a cost – there is a licence requirement to run this programme. |
| **Feasibility / Development studies** | N/A |
| **Aim** | Aims of FRIENDS for Youth ProgramSupporting children's mental health and wellbeingEnhancing school achievement & employmentAims of Rodgers & Dunsmuir 2015The first aim of this study was to investigate the impact of the ‘FRIENDS for Life’ intervention, delivered in a school setting on  ‘overall anxiety’ and the spectrum of anxiety subtypes as detailed in the DSMIV-TR. The second aim was to examine the dyadic agreement between child and parent ratings of the child’s anxiety at different time points during the intervention. The third aim was to examine the relationship between anxiety and school adjustment in more depth. Finally, this study aimed to explore whether the ‘FRIENDS for Life’ programme may indirectly improve these outcomes through reducing anxiety. |
| **Research Question / Hypothesis** | N/A |
| **Intervention** | |
| Developed by | The ‘FRIENDS for Life’ emotional resiliency programme is a group CBT intervention that was developed by Professor Paula Barrett at the Pathways Institute in Australia.The word ‘FRIENDS for Life’ is an acronym that helps children and adolescents to remember the steps to follow when feeling anxious:**F** – Feeling Worried?**R** – Relax and feel good.**I** – I can do it!**E** – Explore solutions and coping step plans.**N** – Now reward yourself.**D** – Don’t forget to practice.**S** – Stay cool! It is a structured, 10-session CBT programme.The programme has three main components based on **CBT principles**:(a) Learning/Behaviour - involves helping children and adolescents to develop six-step problem solving plans, use coping step plans and identify role models and support networks(b) Cognitive - component involves helping children and adolescents to use positive self-talk (green thoughts), challenge negative self-talk (red thoughts), evaluate themselves realistically and reward themselves(c) Physiological -  involves teaching children and adolescents to be aware of their body clues, to use relaxation techniques and to self-regulate. |
| Therapeutic approach: | N/A. |
| Logic model / Theory of change | How does it work? Theory of Change)Coping skills help youth to manage their anxiety and protect themselves from developing anxiety disorders.The programme aims to teach coping skills such as understanding and managing emotions to assist youth in responding to uncomfortable emotions in appropriate and helpful ways.In the short term, youth have better awareness of their emotions and helpful emotion management techniques that enable them to better cope with stressful or uncomfortable situations.In the longer term, youth will be less likely to develop anxiety disorders. |
| Content | Sessions involve large and small group work, workbook exercises, role plays, games, activities, and quizzes. In addition, to reinforce and generalise the skills introduced in the sessions, homework tasks are assigned at the end of each session. It has three main components:Learning/behaviour – involves helping children and adolescents to develop six-step problem-solving plans, using coping step plans, and identify role models and support networks.Cognitive – involves helping children and adolescents to use positive self-talk, challenge negative self-talk, evaluate themselves realistically, and reward themselves.Psychological – involves teaching children and adolescents to be aware of their body clues, to use relaxation techniques, and to self-regulate. |
| Sessions: | FRIENDS for Youth is delivered in five sessions of 2-2.5 hours’ duration each, by one social worker, teacher, health worker, or psychologist to groups of children. In the best-evidenced implementation of FRIENDS, the programme was delivered in 10 sessions of one hour each. |
| Delivery Slot | Regular school hours during Social Personal and Health Education (SPHE) classes. The control groups received no active intervention during this time. |
| Dosage | 60 min sessions |
| Duration | 10 weeks |
| Delivery agent | First author of the paper delivered all sessions.The practitioner who delivers this programme is a psychologist, teacher or allied health professional with QCF-6 level qualifications and received eight hours of programme training. (booster training of practitioners is recommended). |
| Delivery Strategies / tools | N/A |
| **Control condition** | Wait-list control group |
| **Randomisation** | The 62 students were randomly allocated to an intervention or wait-list control group within each respective school. |
| **Fidelity checks** | A protocol integrity check was carried out during this study to ensure fidelity of implementation. Random ‘FRIENDS for Life’ sessions were videotaped. On viewing the sessions, a checklist indicating compliance with the manual content for these sessions was completed by a second trained researcher. The protocol integrity checks showed concordance between session and manual content (89%).What are the systems for maintaining fidelity?Training manualOther online materialFace-to-face trainingFacilitator manualApparently, supervision of practitioners is not required. |
| **Outcome measures** | Rodgers & Dunsmuir: The study assessed children’s anxiety on the Spence Children’s Anxiety Scale (SCAS) and Spence Children’s Anxiety Scale for Parents (SCAS-P), using the child self-report version and the parent-report version. In addition, the study assessed school adjustment on the child-report and teacher-report versions of the Child Rating Scale (CRS) and the related teacher measure (T-CRS 2.1) |
| **Findings** | Significant effect for anxiety symptoms at immediate post-int, sustained at 4 mth follow-up.anxiety scores reduced significantly between preintervention and postintervention (t(31) = 2.381, p < .05) whilst the control groups’ did not (t(29) = .384, p = .7). The intervention groups’ anxiety scores also reduced significantly between postintervention and follow-up (t(31) = 4.985, p < .01) whilst the control groups’ did not (t(29) = 1.885, p = .07.Follow-up t-tests revealed that the intervention groups’ parent anxiety scores reduced significantly between preintervention and postintervention (t(29) = 2.02, p < .05) whilst the control groups’ did not (t(26) = .658, p = .517) |
| **Moderators / mediators** | N/A |
| **Author comments** | N/A |
| **Limitations** | The three schools who participated in this study educated students from low socioeconomic backgrounds. Therefore, questions about the representativeness of the sample and ultimately the generalisability of the results must be raised. Analysis based on self-reported subjective interpretation of anxiety. |
| **Team review** | Age-appropriateness is a problem. But has problem-solving approach and may act as stress-reduction for general population.  Discussed with programme creators potential for sharing for cultural adapattion – not possible |
| **Decision** | Reject |

**Manual Search between 2017-2021 (since the last systematic reviews were published)**

Based on an initial search most interventions identified in the manual search did not meet our inclusion criteria due to:

- Being fully online or having a strong online component (use of an app). This was the case for the majority of additional programs identified.
- Interventions targeting age ranges outside that required for SAMA.
- Interventions being indicated or selective.
- Interventions being based fully on mindfulness / physical activity or having a strong component of them.
- Interventions not conducted in a school-based setting.
- Recruited adolescents with clinical depression or anxiety symptoms (potentially not suitable for a universal approach).
- Interventions previously excluded from the SAMA systematic review search.

Two eligible interventions were identified from the manual search:

| **Intervention name** | Discover |
| --- | --- |
| **Paper Name** | Brown et al (2019) |
| **Country** | UK |
| **Institute** | Kings College London |
| **Available** | Yes, available at a cost. Dr Irene Sclare, DISCOVER programme lead and Consultant Clinical Psychologist |
| **Feasibility / Development studies** | Brown, J.S., Blackshaw, E., Stahl, D., Fennelly, L., McKeague, L., Sclare, I., & Michelson, D. (2019). School-based early intervention for anxiety and depression in older adolescents: a feasibility randomised controlled trial of a self-referral stress management workshop programme (‘DISCOVER’). *Journal of adolescence, 71,* 150-161. |
| **Aim** | The objectives were to:1. Assess feasibility of recruiting and randomising 10 schools.2. Assess student attendance at information meetings, consent rates and participant attendance at DISCOVER workshops3. Assess participation rates for students by demographic group (e.g. gender, ethnic minority, year of schooling), help-seeking profiles (e.g. whether sought help before) and clinical profiles (e.g. severity of symptoms)4. Assess data collection rates at baseline and 3-month follow-up.5. Obtain outcome variance estimates and intraclass correlations (ICC) required for sample size calculations in a full-scale trial.6. Explore the likely ranges for candidate outcomes by obtaining intervention effect estimates and confidence intervals for depression and anxiety. |
| **Research Question / Hypothesis** | See above. |
| **Intervention:** | |
| Developed by | The DISCOVER ‘How to Handle Stress’ workshop programme was adapted from an adult version (Brown, Cochrane, & Cardone, 1999) and refined through formative field testing and feedback from a Teenage Advisory Group (Sclare et al., 2014). |
| Therapeutic approach | CBT based. |
| Logic model / Theory of change | N/A |
| Content | Workshop content: This focused on methods for coping with common personal and academic stresses experienced by older adolescents, such as social anxiety and worry, dealing with coursework, dealing with family expectations and exam anxiety. CBT principles and methods included psycho-education about the CBT model, fear exposure, thought challenging, mindfulness, problem solving, sleep hygiene and time management which were all explained and demonstrated. Video vignettes were also used to illustrate the difficulties and effects of stress on a young person's thoughts and actions, and demonstrated the techniques in practice. Participants were given workbooks and encouraged to set a personal goal as homework and offered a telephone goal review. Personal goals were selected by students and could relate to social, personal, relationships or schoolwork. Many participants chose to work on goals related to managing school-related difficulties, improving their sleep and managing anxious feelings. |
| Sessions | A one day self -referral workshop in schools. |
| Delivery Slot: | The workshop was delivered face-to-face in a quiet schoolroom over one day. |
| Dosage | 1 day – plus a Personalised telephone (for goal review): As part of the intervention, one week after the workshop, all workshop participants were telephoned by a workshop leader for a 20–30 min call, in order to monitor goal progress and support their use of CBT coping skills. Participants were offered up to two further goal reviews within the 12-week follow-up period. |
| Duration | 1 day |
| Delivery agent | Workshops were co-facilitated by two qualified clinical psychologists and one assistant psychologist, who was a graduate psychologist who had not undertaken clinical training. The team were trained to deliver the workshop according to a detailed manual. To ensure fidelity, workshop leaders completed an evaluation form after each workshop, recording the completion of manualised components. The workshop was delivered face-to-face in a quiet schoolroom over one day. |
| Delivery Strategies / tools | At the DISCOVER workshop, the team use a range of multi-media methods to deliver material, aid learning and engage students. This includes psychologist-led presentations, video material, group discussions, experiential exercises, and goal-setting, to build skills in the strategies and techniques covered and to improve students’ wellbeing outcomes.  Each student gets a DISCOVER Workbook to use within the workshop and to keep. At the end of the workshop day, students are supported to set personal goals, identifying and applying techniques that have been demonstrated during the workshop. The student also has access to the DISCOVERbot app which holds a record of their goal and has a reminder of the workshop techniques. We review students’ progress by phone approximately a week after the workshop, and then, in an individual session, three months later. |
| **Control condition** | Waitlist control condition |
| **Randomisation** | Schools were randomly allocated in a 1:1 ratio to trial arms following completion of baseline assessments. We deliberately randomised the clusters after the recruitment of students to avoid a selection and recruitment bias of participants as recommended by Murphy, Esterman, and Pilotto (2006).Block randomisation (two blocks of five schools) was performed using an online randomisation system managed by an independent Clinical Trials Unit. Randomisation was implemented by the Chief Investigator and schools were informed of their allocation by workshop leaders. The maximum number of participants per workshop was 15. If this was exceeded, extra workshops were run in those schools and a random number generator used to randomise students into two groups. Research workers were blinded to participant allocation, following a protocol requiring minimal contact with workshop leaders and the use of standardised scripts at follow-up. Controls were offered the intervention 3 months after randomisation. |
| **Fidelity checks** | N/A |
| **Outcome measures** | Primary outcomes:Depression symptoms were measured using MFQ.Anxious symptoms were measured using RCADS.Secondary outcomes:Paediatric Quality of Life Enjoyment and Satisfaction Form (PQ-LES-Q)Warwick-Edinburgh Mental Well-Being Scale (WEMWBS) |
| **Findings** | Absolute standardised treatment ES @ 3m were **d = 0.25** (95% C.I. 0.46–0.04) for **anxiety**, **d = 0.27** (CI 0.49–0.04) for **depression**, **d = 0.46** (CI 0.17–0.73) for **well-being** and d = 0.36 (CI 0.09–0.64) for quality of life compared with a wait list control.No differences were found in clinical outcomes at 3 month follow-up for those who did not have any telephone reviews, had one, or two or more reviews. There was a substantial under-representation (18.7%) of males. |
| **Moderators / mediators** | None measured |
| **Author comments** | N/A |
| **Limitations** | Small studyUsed only self report measuresSubstantial under representation of males (18.7% only) |
| **Team reviews** | One session unlikely to be acceptable to schools or young people. Used app also. No long term effects. |
| **Decision** | Reject |

| Shamiri – (“thrive”) | |
| --- | --- |
| **Paper Name** | Osborn et al, 2021 |
| **Country** | Sub-Saharan Africa (LIC) |
| **Institute** | Shamiri Institute, 115 Applewood Adams, Ngong Road, Nairobi, Kenya |
| **Available** | Yes |
| **Feasibility / Development studies** | Pilot Study - Osborn et al 2020 – Group Intervention for Adolescent Anxiety and Depression: Outcomes of a randomised trial with adolescents in Kenya. |
| **Aim** | To test if ‘Shamiri’ can alleviate depression and anxiety symptoms in symptomatic Kenyan adolescents. |
| **Research Question / Hypothesis:** | Can scalable psychological interventions that invoke simple psychological principles, rather than explicit references to psychopathology, alleviate depression and anxiety symptoms in Kenyan adolescents? |
| **Intervention:** The Shamiri Wellness Program is a low-cost, evidence-based intervention for adolescent depression and anxiety. To overcome stigma, it uses positive psychological principles. To overcome resource limitations, it uses lay-providers (local high-school graduates) and is held in schools. | |
| Developed by | The Shamiri Institute whose mission is to develop and deploy evidence-based interventions that improve the health, wellness, social, and academic outcomes of children and adolescents in East Africa.Previously tested (The above-mentioned pilot study) with students in Kibera (the largest slum in Africa). When compared to a study skills control group, the Shamiri Wellness ProgramSignificantly reduced depression and anxiety symptoms.Significantly improved academic performance.Significantly improved social support from friendships. |
| Therapeutic approach | Positive Psychology |
| Logic model / Theory of change | N/A |
| Content | For adolescents aged 13-18 years old.The main concepts of Shamiri are:**Growth:** Targeting theories about the ability to improve over time, teaching students that personality traits, skills, and situations can be improved through effort and strategies.**Gratitude:** Grateful people exhibit more positive experiences and outcomes. Students in the Shamiri program learn about gratitude and practice gratitude exercises.**Values and virtues:** Work with students to identify and reflect on specific values that are most important to them. Such a self-reflection can improve individual coping skills by re-establishing an awareness of personal worth, goals, and integrity.**Shamiri:** The Shamiri intervention included **3 modules**: **growth mindset** (2 sessions), **gratitude** (1 session), and **value affirmation** (1 session). |
| Sessions: | \| The intervention consists of **four** intervention sessions, **one-hour long and one week apart.** All include reading activities, group discussions, and writing activities. Homework exercises between sessions. In sessions one and two, participants learn about growth, neuroplasticity, and work to build their growth-mindsets. In sessions three and four, participants learn about gratitude and value affirmations respectively. \| \| \| --- \| --- \| \| Session 1 – Growth \| introductions and an ice-breaker activity; didactic introduction to growth and personal improvement in different areas—social, academic, and emotional—and their benefits. Participants read article and watch a short video that explains neuroplasticity: a phenomenon whereby the brain grows and makes new connections as a result of learning and practicing new skills. Both the article and video script are available in supplementary materials; the video is available upon request and online. Next, the participants read three growth testimonials from local Kenyan peers and a growth testimonial from their own group leader. Testimonials focus on personal growth in a plethora of domains (e.g., school performance, personality, happiness, relationships, etc.). Participants then have a discussion about the testimonials. To conclude the first session, participants are assigned a take-home activity in which they identify a challenge they faced, describe how they used effortful strategies to deal with the challenge, and reflect on how they grew as a result of the challenge. \| \| Session 2 - Growth \| In session two, participants begin by discussing their take-home activity. They then brainstorm and discuss effective strategies that they can use to apply the lessons of growth-mindset in their own lives. Next, the group leader will moderate a discussion about problem-solving skills modelled off of the problem-solving STEPS module. Afterward, participants are instructed to write a letter to a friend in which they explain the concepts that they have learned thus far—including neuroplasticity, growth- mindset, effective strategies, and problem-solving skills—in their own words. The group leader is instructed to do all of the same activities themselves while the participants do them. Lastly, participants are assigned a take-home activity in which they think of one problem that currently affects them and brainstorm solutions modelled off the problem-solving skills learned in the session. They are encouraged to try one or more of these solutions during the week. \| \| Session 3 - Gratitude \| In session three, students learn about gratitude. The group leader opens the session with a didactic introduction to gratitude and its various benefits. The participants then discuss gratitude and identify people and things for which they are grateful. After this, participants write a “gratitude letter” to a living person who has changed their life for the better. For homework, participants are instructed to complete a daily “three good things” activity during the following week. In this activity, participants identify and write three good things that happened each day and write a short reflection for each. \| \| Session 4 – Virtues \| In session four, students learn about virtues and their importance in everyday life. While the activities in this section are based on empirically tested value affirmations exercises, the discussion of values will be presented to Kenyan students as “virtues” due to differences in language and vocabulary. The group leader opens the session with a didactic introduction to what virtues are. The group then enters a discussion about virtues, noting why they are important in guiding daily behaviour and helping us find purpose in our lives. Students are then asked to select from a long list several virtues that are meaningful to them. Next, students select the one particular virtue that they feel is most important to them and describe, in writing, why this virtue is important, a time when they have lived up to that virtue, and how they can live in better accord with this virtue in the future. There is no take-home activity following this fourth and last session. At this point, participants are allowed to keep the Shamiri booklet, which contains all the lessons and exercises from the four sessions for both the Shamiri intervention and the control. \| \| **Study -Skills control** \|  \| \| Using a study skills control group (Active control) both presents participants with an opportunity to benefit from participation and provides a more rigorous standard of comparison than passive controls. The control condition consists of four modules: a note-taking module lasting one session, an effective-study-strategies module lasting one session, a time management module lasting one session, and a study cycle module lasting one session. All these modules were developed specifically for a pilot trial of the Shamiri intervention. To control for non-specific aspects of the intervention, the study skills control group (developed for the present study) mirrors the structure of the Shamiri intervention. Each condition contains the same number of exercises and group discussions, within-session activities are similar in format (e.g., reading, writing, and discussion activities), and between-session take-home assignments require similar effort. \| \| \| Session 1 – Note-Taking \| During session one, group leaders will offer a didactic introduction to the 5Rs of note-taking. Then, participants will complete an exercise to practice the new note-taking method: They will read a short article about climate change and take notes using the 5Rs strategy they just learned. For homework, participants will be asked to complete three tasks: They will 1) identify one particular class during which they used the 5Rs of note-taking, 2) describe how they used the 5Rs to take notes, and 3) reflect on whether their learning improved as a result of using the new note-taking strategy. \| \| Session 2 – Effective Study Strategies \| To begin session two, participants will reflect on the homework in a group discussion (e.g., What was rewarding or challenging about using the 5Rs? Do you plan to use them in the future?). Then, group leaders will introduce ten effective study strategies. Participants will be asked to discuss these ten strategies, examples of when they have used them, and other strategies that have worked for them. For homework, participants will be asked to complete three tasks: They will 1) identify a specific academic challenge they face over the course of the following week, 2) employ one strategy from the ten discussed in this session to address the challenge, and 3) reflect on whether their learning improved as a result of using the new strategy. \| \| Session 3 – Time Management \| To begin session three, participants will reflect on their homework assignment in a group discussion (e.g., Which strategies worked for you? Which ones did not? Why? What strategies might you use in the future?) Then, group leaders will introduce the concept of time management and lead a discussion about the four steps to improving it as a skill. For homework, participants will be asked to track and write down how they spend their time on activities like schoolwork, sleeping, and eating. This homework is intended to help participants understand how they are actually spending their time each day. \| \| Session 4 – The Study Cycle \| To begin session four, participants will reflect on their homework assignment in a group discussion. Group leaders will ask if anybody is willing to share their time management worksheet and discuss with the group. Then, group leaders will introduce the five-step study cycle and discuss it with the students. Finally, group leaders will lead a discussion about how participants can continue practicing skills they have learned from all the sessions in the future. There is no take-home activity following this fourth and last session. At this point, participants are allowed to keep the Shamiri booklet, which contains all the lessons and exercises from the four sessions of the study-skills sessions. \| |
| Delivery Slot | Groups included 7 to 15 youths (mean group size of 9 adolescents).  conducted in a combination of English and Kiswahili because all participants spoke both languages. Groups met in the schools during the afternoon time designated for extracurriculars. |
| Dosage | 4 weekly X 60-minute sessions. |
| Duration | 4 weeks |
| Delivery agent | Lay group leaders (aged 18-26 years; 8 of 13 female [61.54%]) for the Shamiri and study skills groups were Kenyan high school graduates fluent in English and Kiswahili who were hired and trained by the study team. They were selected via a semi-structured interview that gauged past experiences and interpersonal skills; they underwent 10 hours of training. |
| Delivery Strategies / tools | Study team-led training was ten hours in total, split into two consecutive days of five hours each. Training consisted of learning the protocols (for both the Shamiri intervention and the study skills control intervention), learning peer counselling techniques (e.g., validating, asking open-ended questions, etc.), and role-playing parts of the protocol. Group leaders were quizzed on intervention material before and after training in order to determine efficacy of the training. Group leaders also completed training feedback forms directly following training and again directly following the end of the four-week intervention. During each week of the intervention, the study team and group leaders met to review that week’s intervention content, discuss any issues that had arisen in groups, and answer any questions that came up. All group leaders for all conditions were supervised in this manner – Their training protocol can be found here -  *Venturo-Conerly KE, Roe E, Wasil A, Osborn TL. Training and Supervising Lay-Providers in Low-Income Settings: A Mixed-Methods Study of Task Sharing from the Shamiri Randomized Controlled Trial. Open science Framework; 2020. Doi:10.31219/osf.io/bqznm* |
| **Control condition:** | Study Skills control condition (active control) as this condition had a large component of concern for academic performance. Thus, dejection and hopelessness about poor grades, and fears of academic failure, can readily be associated with depression and anxiety symptoms. In this context, the study-skills training condition is potentially addressing a source of anxiety and depression symptoms, in addition to providing skills of genuine practical value for the students. |
| **Randomisation:** | Single-blind, parallel-group randomisation. |
| **Fidelity checks:** | Intervention fidelity was assessed using session audio recordings to evaluate layperson adherence to and competence in protocol components. Two independent raters, who were not affiliated with the study team, independently rated a randomly selected 10% of sessions. Interrater agreement was assessed for each domain, all were in the acceptable range, for delivering protocol content, for completing specific tasks (e.g., distributing worksheets), for thoroughness, skilfulness, clarity, and for including content exclusively from the relevant session protocol. |
| **Outcome measures:** | **Primary Outcomes:**Depression symptoms were assessed using the **Patient Health Questionnaire–8 item (PHQ-8);** the 8-item version of the PHQ-9.Anxiety symptoms were measured using the **Generalized Anxiety Disorder–7 item (GAD-7).****Secondary Outcomes**At the end of the last session, participants completed a program evaluation survey. On a scale of 1 to 5, participants rated “How helpful was the program as a whole to you?” and “How likely would you be to recommend the program to a friend?” |
| **Findings** | A total of 307 youths completed the 4-week intervention. Both Shamiri and study skills were rated highly useful (4.8/5.0) and reduced symptoms of depression and anxiety, but analyses with imputed data revealed that youths receiving Shamiri showed greater reductions in depressive symptoms at posttreatment (Cohen d = 0.35 [95% CI, 0.09-0.60]), 2-week follow-up (Cohen d = 0.28 [95% CI, 0.04-0.54]), and 7-month follow-up (Cohen d = 0.45 [95% CI, 0.19-0.71]) and greater reductions in anxiety symptoms at posttreatment (Cohen d = 0.37 [95% CI, 0.11-0.63]), 2-week follow-up (Cohen d = 0.26 [95% CI, −0.01 to 0.53]), and 7-month follow-up (Cohen d = 0.44 [95% CI, 0.18-0.71]). |
| **Moderators / mediators** | Underpowered and therefore did not preregister or conduct tests of potential moderators (e.g., gender or tribe). |
| **Author comments** | N/A |
| **Limitations:** | High attrition (due to government ban).Measures were not designed or fully validated for Kenyan youths.Absence of a suicide item on the PHQ-8 limited their ability to gauge intervention effects on suicidality. |
| **Team review** | Strengths – low cost, uses the equivalent of lay counsellors, duration of intervention, effectiveness. Limitations – no active ingredient identified. |
| **Decision** | Progress to include in co-adaptation. |

Supplementary File 8: ADAPT Step 5 Development of Intervention Prototypes

This file reports the content and process for the development of each intervention prototype.

**Intervention 1 Prototype: SAMA for YOUTH**

The prototype was generated from:

- Effective and freely available whole school intervention manuals to prevent anxiety and / or depression (from Review 1).
- Other evidence of ‘what works’ in secondary school mental health programs from three key publications.

**Available effective interventions (from Review 1)**

Only two interventions from Step 2 were possible to take forward to co-adaptation, namely SEHER (Shinde et al., 2018) and SHAMIRI (Osborn et al., 2020). SEHER was developed and tested in India and emphasized the importance of a positive school environment to student wellbeing. Its aims were to (i) promote adolescents’ social skills; (ii) engage the whole school community in school decision-making processes; (iii) provide factual knowledge to the school community; and (iv) enhance adolescent problem-solving skills. We had already identified SEHER as a foundational program prior to the umbrella review. SHAMIRI was developed in Kenya as a low-cost, evidence-based intervention for adolescent depression and anxiety using positive psychological principles and lay-providers. Table S7.1 documents which aspects of SEHER and SHAMIRI were retained and /or adapted in the SAMA for YOUTH (and for completeness, SAMA for TEACHERS / SCHOOLS).

**Table S8.1 SEHER and SHAMIRI subject topics and their exclusion or inclusion (with or without adaptation) in SAMA for YOUTH and SAMA for SCHOOLS prototypes**

| Topics Covered in SEHER | Yes / No  Retained in SAMA Prototype | SEHER Topic Details | Inclusion / Adaptation in SAMA |
| --- | --- | --- | --- |
| Health and Hygiene | X not mental health focused |  | |
| Zero Tolerance to Bullying | ✓ mental health focused | Types, causes and effects of bullying.  Dealing with bullying. | Extended focus on anti-bullying  SAMA for YOUTH Session 4: Problem-solving and Session 7: Building and Keeping Good Relationships.    SAMA for SCHOOLS includes a focus on bullying in peer workshops and whole school anti-bullying policies.  In SAMA for TEACHERS to emphasise harmful effects of school bullying on adolescents. |
| Substance use | X relevant to mental health but often covered by schools in different ways |  | |
| Gender and violence | X relevant to mental health but to broad for inclusion |  |  |
| Mental Health | ✓ mental health focused | Different emotions and their effects. | SAMA for YOUTH Session 1: Emotion Spiral. |
|  |  | Anger and sadness: signs, causes, effects and dealing with them. | Partly included SAMA for YOUTH Session 5: Managing difficult feelings. |
|  |  | Stress, its effect and dealing with it. | SAMA for YOUTH Session 3: Managing Pressure at school. |
|  |  | What is mental health? Building positive mental health. | SAMA for YOUTH Session 1: Getting Started and Session 2: Top Tips for Feeling Good and Session 8: Putting it all together. |
|  |  | What is depression, suicide, anxiety, symptoms and dealing with them. | SAMA for YOUTH Session 5: Managing difficult feelings (low mood). |
| Reproductive and Sexual Health | X not mental health focused |  | |
| Effective Study Skills | X not mental health focused |  |  |
| Rights & responsibilities | X not mental health focused |  |  |
| Topics Covered in SHAMIRI | Yes / No  Retained in SAMA Prototype | SHAMIRI Topic Details | Inclusion / Adaptation in SAMA |
| Growth | ✓ mental health focused (psychological flexibility and possibility of change) | Growth and personal improvement (school performance, personality, happiness, relationships); strategies to apply growth-mindset; problem-solving skills. | SAMA for YOUTH Session 1: Concept of growth mindset. Re-connecting with concept in later sessions |
| Gratitude |  | Gratitude and its benefits; daily “three good things” activity for one week. | SAMA for YOUTH Session 3: Living with Values |
| Virtues |  | Virtues and their importance in everyday life; value affirmations; purpose in life. | SAMA for YOUTH Session 3: Living with Values |

**Other evidence of ‘what works’ in secondary school mental health programs**

We reviewed the following sources for common effective mechanisms in school mental health programs for adolescents:

- Clarke, A., Sorgenfrei, M., Mulcahy, J., Davie, P., Friedrich, C., & McBride, T. (2021). Adolescent mental health: A systematic review on the effectiveness of school-based interventions. *Early Intervention Foundation*, 1-27.
- Skeen, S., Laurenzi, C. A., Gordon, S. L., Du Toit, S., Tomlinson, M., Dua, T., ... & Melendez-Torres, G. J. (2019). Adolescent mental health program components and behavior risk reduction: a meta-analysis. *Pediatrics*, *144*(2).
- Gimba, S.M., Harris, P., Saito, A. *et al.* (2020). The modules of mental health programs implemented in schools in low- and middle-income countries: findings from a systematic literature review. *BMC Public Health* 20,158. <https://doi.org/10.1186/s12889-020-09713-2>

**Table S8.2 Components of school mental health programs with evidence of effectiveness that were included in SAMA for YOUTH prototype taken to co-adaptation workshops (highlighted in bold).**

|  | Reviewed publications on components of  school mental health programs | | | | Where included in SAMA for YOUTH Prototype |
| --- | --- | --- | --- | --- | --- |
| **Publications** > | Clarke et al. (2021) Effective components of school mental health programs for adolescent wellbeing (WB) or symptoms of anxiety and depression (A&D) | Skeen et al. (2019) Effective components (ES=effect size) of face-to-face programs on symptoms of anxiety and depression (A&D) | Gimba et al. (2020) (modules in LMIC school mental health programs) |  | |
| Strengthening Positive Emotions (when delivered as activities that can be easily implemented into daily routines) | Associated with small effects on A&D and WB |  |  | Session 2: Protecting Wellbeing    Incorporated in form of IF-THEN plans in many session | |
| Self-Awareness | Associated with medium effects on A&D |  |  | Session 1: Emotion Spiral  Session 3: Living with values    Minute mindfulness practice in all sessions | |
| Interpersonal skills |  | Most consistently associated with large ES on A&D | as 'Relationships & Communication' (inc. self-awareness, empathy, being a friend) | Session 7:  Building positive relationships    Session 8: Being supported | |
| Emotion regulation |  | Associated with large ES for positive mental health and A&D | in indicated programs (including emotional awareness) | Sessions 2: Protecting wellbeing | |
| Alcohol and drug education |  | Associated with positive mental health |  |  | |
| Mindfulness | No effects on A&D; small effects on WB | Associated with reduced A&D |  | Minute practice at started and end of each session | |
| Problem-solving |  | Associated with reduced A&D | As 'Cognitive' (inc. problem-solving, anger management, decision-making and critical / creative thinking) | Session 5: Difficult Thoughts and Problem-Solving | |
| Assertiveness Training |  | Associated with large ES for prevention of substance abuse |  |  | |
| Behavioural activation |  | N/A |  |  | |
| Stress Management |  | Associated with large ES for stress reduction |  | Session 5: Difficult Feelings | |
| Cognitive Restructuring | Most evidence for effects on A&D is for programmes drawing on cognitive behavioral concepts | Associated with very small ES on A&D | As 'Coping Skills' (inc. Managing emotion and stressful situations' | Session 5: Difficult Thoughts and Problem-Solving | |
| Psychoeducation / Mental Health Literacy |  | No effects on A&D | In indicated programs,  e.g signs and symptoms | Incorporated throughout sessions | |
| Self-esteem |  | N/A | In indicated programs |  | |
| Conflict resolution |  | Associated with very small ED on A&D |  | Session 7: Building positive relationships (addressing bullying) | |
| Coping skills |  | No effects on A&D |  | Session 2:  Protecting Wellbeing | |
| Relaxation |  | No effects on A&D | In indicated programs | Session 2:  Protecting Wellbeing | |
| Goal setting |  | Associated with very small ED on A&D |  | Incorporated in form of IF-THEN plans in many session | |
| Resisting peer pressure |  | Associated with very small ED on A&D |  | Session 7: Building positive relationships | |
| Self-efficacy |  | Associated with very small ED on A&D |  |  | |
| Self-monitoring |  | No effects on A&D |  |  | |
| Social Skills |  | No effects on A&D |  |  | |
| Support networking |  | No effects on A&D |  |  | |

Additionally, the SAMA for Youth *Intervention* prototype 1 was informed by *WHO's* (92) *Helping Adolescents Thrive Toolkit* *guidance on* Health Promoting Schools [Publication Item,](https://www.who.int/publications/i/item/9789240025554) namely:

- Importance of a whole school approach.
- There is engagement and collaboration within the school community, including with students.
- The school curriculum supports physical, social-emotional and psychological aspects of student health and well-being.
- The school has a safe and supportive social-emotional environment.

**TABLE S8.3 The final SAMA for YOUTH Intervention Prototype brought to the co-adaptation workshops.**

| Sessions | Basic Content |
| --- | --- |
| 1) Getting started, setting intentions | Student-led ground rules. Exploring the concept of well-being. Identify barriers to well-being for adolescents. Students set three personal well-being goals. |
| 2) Protecting our well-being | Psychoeducation; goal setting; behavioural activation. Notion of ‘protecting’ our well-being; comparison to physical health; map well-being in inner and outer life; ways of being associated with good well-being. Identification of small changes to protect well-being. |
| 3) Living our values | Values; explore concept; why values matter to well-being; identify personal values; students practice living out a particular value in the week. |
| 4) Challenges to well-being | Psychoeducation; emotional regulation; growth mindset; what brings us down? Normalising and empathising with difficult and emotional situations; building awareness of thought and emotions at these times; Growth mindset – we can learn to manage these; identify and emphasise students’ past use of effort and strategies. |
| 5) Managing difficult feelings | Emotional regulation; identify triggers and common feelings; practice strategies for reducing emotional arousal; student practice one emotion-based strategy in the week. |
| 6) Managing difficult thoughts & problem solving | Explore concept. Common thoughts in relation to adolescent stressors. How thoughts affect feelings and behaviour. Application of thought-based strategies. Students practice on thought awareness or problem-solving strategy in the week. |
| 7) Relationships | Interpersonal skills. Why relationships matter to well-being. When relationships are hard/helpful. Getting and giving the best out of relationships (connecting to values). |
| 8) Being supported | When and how to seek help. Dispelling myths. Tackling stigma. How to help friends. |

**Intervention 2 Prototype: SAMA for TEACHERS**

The prototype was generated from

- Effective and available school interventions to promote teacher mental health literacy (from Review 2). Only one intervention from the umbrella review was eligible, namely the Go-to-Educator Training (Kutcher, & Wei, 2018). Table S7.5 document which aspects of this intervention were retained in the SAMA for TEACHERS prototype intervention.
- Other manually searched sources related to teacher mental health literacy and positive practices which were easily available/ accessible, namely: White Swan Foundation’s ‘Youth Mental Health: Teacher as Catalysts’ (2019), Fortis’ Mental Health in Classroom Curriculum (Parikh et al., 2020) and Positive Discipline and Classroom Management by the Centre for Justice and Crime Prevention and the Department of Basic Education, Pretoria (2012). Table S7.6 document which aspects of these interventions were retained in the SAMA for TEACHERS prototype intervention.

**TABLE S8.4 Components of the Go-To Educator Training intervention excluded or included and / or adapted in SAMA for TEACHERS prototype.**

| Topics Covered in GTET | Retained in SAMA Prototype  (Yes / No) | GTET Topic Details | Inclusion / Adaptation in SAMA |
| --- | --- | --- | --- |
| The stigma of mental illness | ✓ | Meaning of stigma, myths and realities of mental illness, and breaking stigma (video discussion and lived experience examples). | Retained the topic but the content was drawn from other manuals that were more culturally appropriate. |
| Understanding mental health and mental illness | ✓ | Understanding teenage brain, the common basis of mental health and mental illness, the concepts-mental health, mental disorder, mental health problems, mental illness, and importance of language. | The concepts of mental health, mental distress, mental health problems were included. Additional sub-topics were added from other sources. |
| Information on specific mental illnesses | ✓ | What happens when the brain gets sick, common mental illnesses, common mental disorders found in teenagers. | Adapted by the team to suit the Indian context (information was also added from other sources). |
| Experiences of mental illness and importance of family communication | × | Impact of mental illness on a young person (discussion through videos) and teens and parents. |  |
| Seeking help and finding support | × | Treatment and recovery, getting help, health questions (communicating with health care provider). |  |
| The importance of positive mental health | × | Understanding stress response, challenging our thinking, reviewing useful strategies for modulating the intensity of the stress response, taking charge of one’s health. |  |

**Table S8.5 Components of teacher mental health literacy and positive practices enhancement programs drawn from other sources. Components that were included in SAMA for TEACHERS highlighted in bold.**

| Components Identified | Source | Yes / No  Retained in SAMA Prototype | Topic Details | SAMA for Teachers |
| --- | --- | --- | --- | --- |
| Understanding Mental Health and Mental Illness | Mental Health in Classroom Curriculum | ✓ | Defining mental health, mental distress and mental health problems, defining mental illness, causes and treatment of mental illness, understanding specific mental illness. | Adapted to suit SAMA. Included details from other sources as well. |
| De-stigmatizing Mental Illness- Rethinking the Social Language |  | ✓ | Understanding stigma, its manifestation, factors, and its relation to mental health. Myths contributing to stigma, and changing stigma and discrimination. | Retained most of the topics. Minor adaptation done to suit SAMA. |
| Encouraging and Promoting Help-Seeking |  | ✓ | Defining help-seeking, importance of early intervention, common barriers to help-seeking, encouraging students to seek help, reaching out for help, supporting a student with mental health problems. | Retained  Defining help-seeking, importance of early intervention, common barriers to help-seeking, reaching out for help, supporting a student with mental health problems. Minor adaptations made to suit SAMA. |
| Building Resilience |  | ✓ | Building resilience, individual characteristics that build resilience, resilience can be learnt, fostering resilience within classroom, strategies for building resilience in students. | Retained as a part of SAMA for Teachers but tailored to some methods. |
| Establishing a Culture of Well-being in the Classroom |  | × |  |  |
| Teacher wellbeing, challenges and influence | Youth Mental Health: Teacher as Catalysts | ✓ | Understanding burden, challenges, and stress. Support, students’ expectations from teachers, teachers’ inspiration. | Retained as a part of SAMA for Teachers. Tailored to exclude the discussion on burden and added the importance of mental health for teachers. |
| Understanding adolescent mental health |  | ✓ | Understanding adolescents, the visible and invisible changes in adolescence, understanding the adolescent brain, labeling and stereotyping, behaviours seen in classroom, signs of emotional distress, prevailing issues that students face, investing time in emotional wellbeing. | Retained the topic ‘Understanding Adolescents’. Adapted to suit SAMA for Teachers. |
| Understanding intervention |  | × |  |  |
| Understanding the difference between punishment and discipline | Positive Discipline and Classroom Management | ✓ | What is punishment and discipline? | Retained as a part of SAMA for Teachers. Tailored the methodology to suit the SAMA participants. |
| Defining corporal punishment, emotional punishment and discipline |  | × | Understanding each of these terms. |  |
| Understanding positive discipline |  | ✓ | The teachers’ approach/ style to disciplining in classroom, why educators continue to use corporal punishment? How were the teachers disciplined? Understanding the difference between positive and negative discipline. | Some of the sub-topics were retained and adapted to be suitable for the participants. |
| A whole-school approach to positive discipline |  | × | The key actors in implementing positive discipline. |  |
| Creating a classroom conducive to a positive discipline approach |  | × | What does a well-managed class look like, the components of a well-managed classroom, managing large classes, creating a classroom procedure plan. |  |
| Implementing a positive discipline approach |  | ✓ | Implementing positive discipline in the classroom, implementing positive discipline principles, understanding and responding to child behaviors, handling conflict, practicing how to encourage. | Tailored to suit SAMA (activities were reduced and adapted). |
| Addressing misconduct |  | × |  |  |

**Table S8.6 The final SAMA for TEACHERS Intervention Prototype brought to the co-adaptation workshops.**

| Session | Modules | Topics |
| --- | --- | --- |
| 1. | Teacher well-being, challenges and influence | •Importance of mental health and well-being  •Teacher’s influence on students |
| 2. | Understanding mental health and mental disorders | •Basic concepts of mental health, mental distress and mental health problems  •Mental disorders and treatment paradigms |
| 3. | Destigmatising mental health problems | •Understanding of stigma and its manifestations (facts, factors and myths)  •Evidence-based strategies to reduce stigma in the school setting |
| 4. | Encouraging and promoting help-seeking | •Help-seeking, importance of early intervention and its barriers  •Supporting students with mental health problems |
| 5. | Mental health promotion and wellbeing activities | •Mental health promotion measures/activities- The need  •Practices to promote well-being and stress management |
| 6. | Building resilience | •Resilience, characteristics that build resilience  •Fostering resilience in the classroom |
| 7. | Positive discipline and classroom management | •Understanding positive practices  •Why harsh discipline could backfire  •Implementing a positive discipline approach  •School’s policy |

**Intervention 3 Prototype: SAMA for SCHOOLS**

The prototype was generated from:

- Effective and available school interventions to promote school climate (from Review 3). No interventions from the umbrella review were eligible. A manual search on the most recent eligible systematic review publication date returned two interventions: SEHER (Shinde et al, 2018) and the Canadian Guide (Go-to-Educator Training, Milin et al, 2016). SEHER’s school climate module was eligible for SAMA. Table S7.7 document which aspects of SEHER were retained in the SAMA for SCHOOL prototype intervention.
- Other evidence of ‘what works’ in secondary school mental health programs from: the Gatehouse Project (Bond & Butler, 2009) and School Climate Improvement Resource Package (U.S. Department of Education, Office of Safe and Healthy Students, 2016).

**Tabl3 S8.7 SEHER components and their inclusion / exclusion in SAMA for YOUTH and SAMA for SCHOOLS prototypes**

| Components | | Details | Informing SAMA Prototypes |  |
| --- | --- | --- | --- | --- |
| Whole School | School mapping and needs assessment | Pre-SEHER school questionnaire to identify school priorities and needs | Retained idea but tailored to include mental health facilities in and around school, mental health training for teachers, presence of committees in school. |  |
|  | Activities to raise health awareness | Lay counsellor facilitates activities such  role-play, speech etc. during the assemblies. Four assemblies/month. | Retained as part of SAMA for SCHOOLS prototype but tailored to mental health topics. |  |
|  | Wall Magazine on health topics | To promote knowledge on the month’s theme. Students & teachers can contribute through write-ups, poems, pictures, artwork | Retained as part of SAMA for SCHOOLS prototype but with monthly theme on mental health topic only |  |
|  | Speak out box (for anonymous raising of student concerns) | Letter box providing a platform for students to raise concerns, complaints, and suggestions anonymously. Opened once a week by lay counsellor and issues are addressed through one-to-one counselling if the student had self-identified. For anonymous inputs, concerns are addressed through discussion during various  activities of the intervention. | Fully retained as part of SAMA for SCHOOLS prototype |  |
|  | School competitions | Monthly eg debate, poster making, sports, quiz, and essay writing linked to the monthly topic of the wall magazine. | Retained as part of SAMA for SCHOOLS prototype but tailored to having only one essay/slogan writing competition in the duration of the intervention. |  |
|  | Healthy School Policies | Schools implement anti-bullying policy and anti-substance use policy | Retained as part of SAMA for SCHOOLS prototype but focused on anti-bullying and a wellbeing policy. |  |
|  | School Health Committee | To ensure that the school environment is safe, healthy and enabling for the students. Meets twice / academic year. Representatives from management, parents, teachers and students. | Retained as part of SAMA for SCHOOLS prototype but re-focused remit on designing whole school activities. Chaired by lay counsellor and including only student representatives and one teacher. |  |
| Group | Peer groups | 10-15 students from each class selected by students to form peer group to support intervention success. Lay counsellor facilitates monthly meetings with peers to discuss the topic of the month and student concerns. | Retained as part of SAMA for SCHOOLS prototype but tailored to provide basic support to the peers and process of referral to the LC, working towards a healthy class environment (preventing exclusion, respecting privacy) and informing about key concerns in class to the LC. |  |
|  | Workshops for students | By lay counsellor for students on changes occurring during adolescence and effective study skills. One per year. | Retained as part of SAMA for SCHOOLS prototype but tailored to include topics related to mental health like anti-bullying, romantic relationship, enhancing concentration and substance abuse prevention. |  |
|  | Workshops for teachers | By lay counsellor to create awareness about SEHER and to sensitize teachers about the concerns of adolescents and provide tips to address them effectively. One per year. |  |  |
| Individual | Student Counselling  Referral Services | Lay counsellor provides problem-solving based  counselling to students (self-referred or referred by teachers) for health complaints, social  difficulties, nutritional problems and academic difficulties. For those students with serious physical  or emotional and behavioural difficulties, referral pathways to specialists are provided. | Retained as part of SAMA for SCHOOLS prototype but limited to providing brief emotional support and referral to professionals. |  |
|  |  |  |  |  |

**Table S8.8 Components of school environment enhancement programs drawn from other sources. Components that were included in SAMA for SCHOOLS highlighted in bold.**

| Components Identified | Yes/No  Retained in SAMA Prototype | Source | Topic Details | SAMA for SCHOOLS |
| --- | --- | --- | --- | --- |
| Establishment | Yes | Gatehouse Project | Establishment of adolescent health team, raise awareness of the issues, involve whole community. | Some of the roles of the adolescent health team were adapted to SAMA’s school environment team. |
| Review | Yes |  | Review current policies, programs and practices; Identify priorities for action. | SAMA for Schools followed this step of reviewing existing policies related to adolescent wellbeing. |
| Planning | No |  | Plan implementation of evidence-based strategies to enhance security, communication and positive regard. |  |
| Training and implementation | No |  | Provide training and ongoing support for teachers and the broader school community. Implement strategies |  |
| . Evaluation | No |  | Monitor, evaluate and communicate progress. Celebrate achievements. |  |
| Planning for school climate interventions | No | School Climate Improvement Resource Package | Develop a plan for making school climate interventions.  Plan for school climate improvement sustainability.  Develop infrastructure and plan for collecting data.  Assess school’s readiness, needs, capacities.  Evaluate and Refine the Planning Process and Continue Planning for Improvement. |  |
| Engaging stakeholders | Yes |  | Engage Staff, families, students, and the community to roll out initiative.  Evaluate and refine engagement efforts with stakeholders and continue planning for improvement and sustainability. | Tailored this to forming a committee involving representatives from SAMA schools’ staff, parents and students. |
| Collecting and reporting school climate data | No. |  | Collect and analyze data. Disseminate findings. Evaluate and refine data collection, reporting protocols, and continue planning for improvement and sustainability. |  |
| Choosing and implementing school climate interventions | No |  | Choose, plan and implement interventions.  Evaluate and Refine the Choice of Interventions and Implementation Strategies and Continue.  Planning for Improvement and Sustainability. |  |
| Monitoring and evaluating school climate improvements | No |  | Evaluate the school climate improvement process. Evaluate and refine the overall effort and continue planning for improvement and sustainability from a multi-tiered perspective. |  |

**Table S8.9 The final SAMA for SCHOOLS intervention prototype brought to the co-adaptation workshops.**

| Session | Intervention Activity | Details |
| --- | --- | --- |
| 1. | School Network Committee | A committee that shall connect the representatives of all the SAMA Schools. |
| 2. | Whole School Awareness Generation/ Assembly announcements | Focus is on generating awareness about SAMA, SAMA activities and mental health topics. |
| 4. | School Environment Team (SET) | This team from each school supports the SAMA lay counsellors whenever required in delivering various SAMA activities. The members coordinate various activities and raise awareness about the same. |
| 5. | Speak Out Box | A box is placed near the washrooms. All the students can share their thoughts, worries, happy thoughts and suggestions through the box. The SAMA lay counsellor opens the box daily or alternatively and addresses any concerns that are dropped in the box. |
| 6. | Wall Magazine | A monthly theme-based periodical which is run on a display board in the school. |
| 7. | Intra School Competitions | Competitions are held on significant days with topics related to emotional well-being. |
| 8. | Emotional Well-Being Policies | Development and implementation of policies like anti-bullying, anti-corporal punishment policy, gender sensitivity. |
| 9. | Peer Support Group | •Provide basic support to the peers and process of referral to the lay counsellors.  •Working towards a healthy class environment (preventing exclusion, respecting privacy).  •Informing about key concerns in class to the lay counsellors. |
| 10. | Workshops | Sessions on anti-bullying and other topics requested by students are conducted in classrooms. |

**Intervention 3 Prototype: SAMA for PARENTS**

The prototype was generated from the following sources for common effective mechanisms in enhancing parent mental health literacy:

- Dawson KS, Watts S, Carswell K, et al. Improving access to evidence-based interventions for young adolescents: Early Adolescent Skills for Emotions (EASE). World Psychiatry: *Official Journal of the World Psychiatric Association* (WPA). 2019 Feb;18(1):105-107. DOI: 10.1002/wps.20594. PMID: 30600639; PMCID: PMC6313693.
- Hugh-Jones, S., Pert, K., Kendal, S., Eltringham, S., Skelton, C., Yaziji, N., & West, R. (2022). Adolescents accept digital mental health support in schools: A co-design and feasibility study of a school-based app for UK adolescents. *Mental Health & Prevention*, *27*, 200241.
- Wei, Y., & Kutcher, S. (2014). Innovations in practice: ‘Go‐To-Educator Training’ on the mental health competencies of educators in the secondary school setting: A program evaluation. *Child and Adolescent Mental Health*, *19*(3), 219-222.
- YoungMinds. (n.d.). *Parents’ guide to looking after yourself*. Retrieved September 2, 2024, from <https://www.youngminds.org.uk/parent/parents-guide-to-looking-after-yourself/>

**Table S8.10 Components of parent mental health literacy programs with evidence of effectiveness. Components that were included in SAMA for Parents are highlighted in bold.**

|  | Reviewed publications on effective components of parent mental health literacy programs | | | SAMA for Parents |
| --- | --- | --- | --- | --- |
| Effective Components Identified | Dawson et al. (2019)  EASE | Wei et al. (2014)  Go-To-Educator Training | YOUNGMINDS |  |
| Psychoeducation | Adversities and emotional distress were addressed. Further understanding own emotions were also addressed. | - | - | Was not suitable for Indian parents / parenting |
| Improving mood | Session focused on strategies to improve mood through changing actions. | - | - | Was not suitable for Indian parents / parenting |
| Problem solving skills | Session focused on managing parents’ problems. | - | - | Was not suitable for Indian parents / parenting |
| Relapse prevention | Different strategies to prevent relapses were addressed. | - | - | Was not suitable for Indian parents / parenting |
| Youth mental health: Complexities and considerations | - | Epidemiological data on adolescent mental health issues, causes and correlations and pathways to care. | - | Adapted to Indian context |
| Stigma of Mental illness | - | Myths and strategies to fight stigma. | - | Adapted to Indian context |
| Talking to parents/family | - | Tips for connecting with parents or family to facilitate identification. | - | Was not suitable for Indian parents / parenting |
| Tips and tricks to deal with adolescents | - | - | - | Adapted to Indian context |
| Coping with common issues | - | - | - | Was not suitable for SAMA |
| Adolescent brain | - | - | - | Was not suitable for SAMA |
| Parenting in digital world | - | - | - | Was not suitable for SAMA |
| Self-harm advice | - | - | - | Was not suitable for SAMA |
| Parents Guide to helping your child and Looking after Yourself | - | - | Show love and pride regularly, set clear boundaries, and be honest about your feelings. Don’t hesitate to ask for help and take time for self-care. Understand that making mistakes is okay and seek professional support if needed. You’re doing your best, and your care makes a difference. | Retained as it is and changed General practitioner to Mental health professionals |

**Table S8.11 The final SAMA for PARENTS intervention prototype brought to the co-adaptation workshops.**

| Sessions | Details |
| --- | --- |
| Session 1: About the SAMA Parent program | An overview of the project and ‘SAMA for Parents’. |
| Session 2: Unwrapping the parents understanding about adolescent mental health | Giving an overview of adolescent mental health. |
| Session 3: Understanding about your child, Parental expectations | Improving parental understanding about the changes in adolescence, discussing the parents’ expectations from their children and discussing the facilitative approach to parenting. |
| Session 4: Adolescent Mental Health and addressing stigma | Myths and facts about adolescent mental health. |
| Session 5: Identification of mental health issues among adolescent children | Details of mental health and mental disorders commonly prevalent in adolescents are given. |
| Session 6: Impact of mental health issues and Helping children with mental health issues | Impact of mental health problems, helping children with mental health problems, getting and providing help. |
| Session 7: Tips to parents’ self-management and let’s all try to practice | Managing oneself and healthy parenting. |
| Session 8: Debrief session | Summarising and concluding. |

Supplementary File 9: Preferred vocabularies and independent expert reviews of three of the intervention prototypes

**Table S9.1** **Participant preferences for intervention vocabulary and concepts**

| **Words/ Phrases** | **Stakeholder Group** | | | |
| --- | --- | --- | --- | --- |
|  | **AB** | **AG** | **T** | **MHP** |
| Emotional well-being |  |  |  |  |
| Anxiety |  |  |  |  |
| Low mood/ depression |  |  |  |  |
| Coping |  |  |  |  |
| Stigma |  |  |  |  |
| Stress |  |  |  |  |
| Mental Illness |  |  |  |  |
| Resilience |  |  |  |  |
| Mental Health |  |  |  |  |
| Help-seeking |  |  |  |  |
| *AB = adolescent boys, AG = adolescent girls, P= parents, T/ HT = teachers / head teachers; MHP = Mental health professionals.*  • *= word understood / acceptable; o = uncertain, ▪ = word confusing/ doesn’t translate well / unacceptable* | | | | |

**Modifications to interventions following co-adaptation: Expert Reviewers’ Feedback**

**Table S9.2 Rating SAMA for YOUTH (Out of 5)**

| **Questions Asked** | **Reviewer 1** | **Reviewer 2** |
| --- | --- | --- |
| The program/material aims are clear (I could understand what the program is trying to do/achieve). | 5 | 5 |
| The program content appears relevant to Indian secondary schools (I think the program will be suitable to offer to Grade 9 students in Indian secondary schools). | 3 | 3 |
| The program content addresses key issues important to adolescent mental health in Indian secondary schools (The program is focused on things that are important to adolescent wellbeing in school). | 5 | 4 |
| The program content is culturally appropriate, e.g. includes issues, values and approaches that will make sense to adolescents in Indian secondary schools. | 4 | 4 |
| The program appears youth-friendly. | 5 | 5 |
| The program is likely to be acceptable to the target audience (The content of the program is likely to be okay for most young people). | 5 | 4 |
| The program appears ethical (The program content seems respectful and safe to deliver). | 5 | 5 |

**Table S9.3 Detailed Reviewer Feedback SAMA for YOUTH**

| **Questions Asked** | **Reviewer 1** | **Reviewer 2** |
| --- | --- | --- |
| Any additional comments relating to the program/material aims? | - | The ultimate goal talks about anxiety and depression but in India, the context in which it emerges is not given much importance because in the clinical context many times family factors play an important role in an adolescent's anxiety/depression. Parent engagement doesn't come at least in this module. |
| Any additional comments relating to the relevancy of the program content? | As such the content seems fine; although I did notice that what was suggested as preferences by adolescents in the co-design process was not necessarily what was included in the module. For instance, relationships was a "maybe" for the adolescents, but occupies 2 sessions in the program. Some of the activities appear a little regressive for an adolescent aged participant. Also, the emphasis on making things humorous through all the sessions maybe unnecessary. | The Indian cultural scenario is not given importance if the program is done in schools catering to upper middle socio-economic category background, then the program is okay. Family relationships and expectations and academic success are considered important goals for parents. Hence, unless there are sessions for parents, the program may not really help. |
| Any additional comments relating to the program content addressing key issues important to adolescent mental health in Indian secondary schools? | - | It does not address academic competence, which teachers and parents value. The majority of the schools do not have remedial classes or school counsellors. Lack of opportunities for extracurricular activities for all children. The temperament plays a huge role in adjustment which should be covered somewhere while discussing individual differences |
| Any additional comments relating to the culturally appropriateness of the program content? | As mentioned earlier, some of the session tasks may be scaled-up in terms of making it developmentally appropriate for adolescents. | I am not sure whether it adequately covers our values because independent thinking and critical thinking are not all that emphasised/encouraged. Students often get frustrated because their independent review is not enabled. School management expects teachers to just focus on academics hence any school mental health program must have at least two to three orientation programs for the adolescent program to be successful. What type of schools does the program is caters to, govt schools with their limited infrastructure, and the negative attitude of the teachers/ parents are some of the hurdles. After the program is done if the students want to approach anyone for help majority of the schools do not have any counsellors. Hardly there are any group activities and opportunities for group work. |
| Any additional comments relating to the program being youth friendly? | - | Introducing problems in a spiral way is an interesting approach. It is youth friendly because it covers important domains of an adolescent's life-developmental stage.   Please refine page no 7. regarding what students can get from SAMA appears too ambitious. Kindly be more specific here. |
| Any additional comments relating to the acceptability of the program by the target audience? | - | The parents' expectations and attitudes towards digital use-use of the internet for social media are not encouraged. This creates frustration. Parents are unhappy because adolescents have not learnt balanced internet use. This is not covered in the program. Handling social media pressure is already becoming a major critical issue in the mental health of adolescents. Out of every 5 adolescents admitted in the inpatient setting of NIMHANS, at least two of them are addicted to internet use which creates feelings of sadness, depression, and self-harm behaviour. |
| Some groups of students may find this content difficult or will not engage with the content? | Yes | Yes |
| If you answered 'Yes' or 'Not Sure' to the above question, please explain your view. | The "yes" is not a reflection of the program; but the sample demographic. There were always be some adolescents who will not engage as much. | They may not open up their real problems or obstacles which they are facing especially in school. It is possible that their ability to verbalise their problems and their knowledge of solutions may be adequate but there may be obstacles either at school/home to actually solve the problems. |
| Any additional comments relating to the program being ethical? | - | Hope care is taken not to take some students for the program if he/she doesn't give assent. Reasons for the same may be explored. |
| We welcome suggestions on ideas/activities that would improve the way we present issues in this program - any ideas? | I have a concern about the long duration (number of sessions). It is currently at 8 mandated sessions plus 4 additional sessions. I am not sure of availability of time within the school curriculum and sustaining adolescent participation throughout the length of the program. It might help to bring down the number of sessions. | The problem-solving component can be introduced first before the problems in schools. CBT taken from the clinical perspective need not be emphasised much because if well-being is the goal then more activities centred around happiness, positive thoughts, and actions need to be highlighted. |
| What do you think will be difficult about delivering this program? Are there any ways to make it better? | As mentioned earlier, the availability of time and adolescents throughout the duration of the program. Bringing down the number of sessions, and possibly not having the extension sessions. | Assuming that these children do not know problem-solving skills may be a mistake. so, if we start the program focussing on what all they know (problems and solutions, what is preventing them from achieving the solution) will give us a good idea as to what to focus on. This has research evidence. several studies have shown that Indian children and adolescents are highly prosocial (parent ratings/self-rating on SDQ) and have very good knowledge of problem-solving alternatives |
| Please give one 'top-tip' to the person delivering this program to improve chances of success. | I understand this question to be a "top-tip" for the SS - Being genuine and authentic in sessions; and not trying too hard to make adolescents engage. There need to be a balance between facilitating and coercing engagement. | The obstacles to the adolescent well-being keeping school and family context is important. |
| Do you have any concerns, additional feedback or advice? | No. All the best :) | Most importantly, school mental health program research over the years have shown promising results in a cross-sectional way. Once the program is over, what will happen to the implementation? Who mentors this? Will SAMA tell that one teacher trained will continue to do some work in helping children maintain well-being? This is the most crucial question asked across the countries wherever a school mental health program is carried out. |

*Note: Feedback is in reviewers own wording and has not been modified.*

**Table S9.4 Reviewer ratings of SAMA for TEACHERS (out of 5)**

| **Questions Asked** | **Reviewer 1** | **Reviewer 2** |
| --- | --- | --- |
| The program/material aims are clear (I could understand what the program is trying to do/achieve). | 5 | 5 |
| The program content appears relevant to Indian secondary schools (I think the program will be suitable to offer to teachers in Indian secondary schools). | 5 | 5 |
| The program content addresses key issues important to adolescent mental health in Indian secondary schools (The program is focused on things that are important to adolescent wellbeing in school). | 5 | 5 |
| The program content is culturally appropriate, e.g. includes issues, values and approaches that will make sense to adolescents in Indian secondary schools. | 5 | 5 |
| The program is likely to be acceptable to the target audience (The content of the program is likely to be okay for most teachers). | 5 | 3 |
| The program appears ethical (The program content seems respectful and safe to deliver). | 5 | 5 |

**Table S9.5 Detailed reviewer feedback SAMA for TEACHERS**

| **Questions Asked** | **Reviewer 1** | **Reviewer 2** |
| --- | --- | --- |
| Any additional comments relating to the program aims? | No | - |
| Any additional comments relating to the program suitability to offer to teachers in Indian secondary schools? | Happy that it is participatory | - |
| Any additional comments relating to the program focus on things important for adolescent wellbeing in schools? | Kindly check the spellings in very few places | - |
| Any additional comments relating to the cultural appropriateness? | Good mix of Audio visuals | - |
| Any additional comments relating to the program acceptability to the target audience? | Very much - Teachers are in need of this information | While most teachers may see the necessity for and utility of such a program. They may also view it as an additional responsibility for which they may not consistently have adequate resources.     Some ways to integrate the SAMA elements with the mandatory curriculum may need to be discussed periodically with the participants, right from the beginning of the sessions. |
| Some groups of teachers may find this content difficult or will not engage with the content? | No | Yes |
| If you answered 'Yes' or 'Not Sure' to the above question, please explain your view. | - | Some sections are very information-heavy, and the corresponding activities do not seem to have adequate time allocated for processing all the information presented. |
| Any additional comments relating to the ethicality of the content? | The resource person should be open to take aspects from the teachers - The program should be conducted from the same platform -- same level as teachers and not in a hierarchical manner saying -- as mental health professionals we know it all and you all have to listen to us | - |
| We welcome suggestions on ideas/activities that would improve the way we present issues in this program - any ideas? | Every juncture check for their inputs -- let’s not forget they deal with students’ day in and day out and they have practical experience of being with students -- | Most of the activities and content are contextualized well to the school system. This is quite helpful.     The section of specific mental disorders could be presented using examples from real-life public figures and followed -up by a quiz to see how they match symptom presentations to possible issues. The focus here could be on recognizing that there is an issue of concern and the pathway they need to follow to connect the concerned student to available support.     When discussing corporal punishment, it may be useful to discuss how our bodies store memories which resurface as actions in similar situations, thereby transmitting the more familiar less helpful patterns even if one has mentally resolved not to use such patterns. The importance of recognizing bodily cues and using soothing/ healing responses in oneself can be emphasized. Vignettes or incomplete sentences can be used to elicit possible bodily responses and cues that require soothing/ healing. |
| What do you think will be difficult about delivering this program? Are there any ways to make it better? | The only challenge that i see is TIME -- May not be able to finish it -- we have to be prepared for it -- | The number of activities may be reduced, and the time allocated for each activity may be increased. Particularly when there are participant interactions and group activities involved.     Participants may benefit from a slower pace of discussion instead of covering the content under pressure of time.     It would also help to have movement-based activities to transition between the core activities mentioned. This would help participants energize themselves, reorganize attention and allow for some emotional release. |
| Please give one 'top-tip' to the person delivering this program to improve chances of success. | Go with the Flow -- Have the Important points that you have to convey - see that you convey those points | Remember to breathe, hydrate and be kind to yourself! |
| Do you have any concerns, additional feedback or advice? | Main --- The resource person should be open for feedback and be able to modify then and there | Teachers may have a lot to share when each topic is introduced and presented. It is important that they are given some protected time for such sharing. This could often be part of the debrief or closing session.     It would also be very helpful for the teachers to have first-hand experience of safety to share, sensitive responses and stress-relief during the delivery of these programs. It will facilitate ease of transfer to the classroom setting. |

**Table S9.6 Reviewer ratings for SAMA FOR PARENTS** (Out of 5)

| **Questions Asked** | **Reviewer 1** | **Reviewer 2** |
| --- | --- | --- |
| The program/material aims are clear (I could understand what the program is trying to do/achieve). | 4 | 5 |
| The program content appears relevant to Indian secondary schools (I think the program will be suitable to offer to parents of Indian secondary school students). | 4 | 5 |
| The program content addresses key issues important to adolescent mental health in Indian secondary (The program is focused on things that are important to adolescent wellbeing). | 4 | 4 |
| The program content is culturally appropriate, e.g. includes issues, values and approaches that will make sense to Indian parents. | 4 | 4 |
| The program is likely to be acceptable to the target audience (The content of the program is likely to be okay for most parents). | 4 | 5 |
| The program appears ethical (The program content seems respectful and safe to deliver). | 4 | 5 |

**Table S9.7 Detailed reviewer feedback SAMA for Parents**

| **Questions Asked** | **Reviewer 1** | **Reviewer 2** |
| --- | --- | --- |
| Any additional comments relating to the program aims? | - | None |
| Any additional comments relating to the program relevancy? | - | None |
| Any additional comments relating to the program addressing key issues important to adolescent mental health in Indian secondary schools? | - | The content could include some of the current trends seen in adolescents like bullying, low self-esteem, turning to unhelpful strategies like technology abuse, dysfunctional interpersonal relationships, emotional abuse, the lack of boundaries etc. |
| Any additional comments relating to the program cultural appropriateness? | - | It would help if parents understood the various triggers associated with troubling behaviour. An insight into the life of a teenager can increase empathy toward their perspective, and can naturally address the resistance we face with this group, helping ease the friction between parents and children. Understanding teenage troubles like low self-esteem, the fear of judgment, and unreasonable parental pressure are some things to consider addressing. |
| Any additional comments relating to the acceptability of the program by the target audience? | - | None |
| Some groups of parents may find this content difficult or will not engage with the content? | No | Not Sure |
| If you answered 'Yes' or 'Not Sure' to the above question, please explain your view. | - | The background and socioeconomic status can bring with it different challenges. Considering these factors are important. |
| Any additional comments relating to the program ethicality? | - | None |
| We welcome suggestions on ideas/activities that would improve the way we present issues in this program - any ideas? | I guess it will help if we have one or two more videos | case discussions work great, and having role-plays also helps. Parents can be divided into 2 groups- one playing the role of the parent, and the other of the teenager. This helps bring out perspectives. Reflecting on the evolution of parenting (how roles and challenges have changed over the years) and understanding the relevance of addressing teenage mental health in the present day will bring better clarity and insight.   Activities that involve visiting one's own childhood- Reflecting on the protective and harmful influences can help understand the context and triggers in one's own parenting style. |
| What do you think will be difficult about delivering this program? Are there any ways to make it better? | It covers all the content required to train the parents, but time management is something which I am concerned about. | Resistance in many forms is the biggest challenge for any mental health program- projecting the content as supportive of emotional and psychological wellness, rather than a reactive process will help.   Drawing parallels between mental and physical health and wellness and giving examples with references to physical issues always help people understand concepts better- Like our readiness and openness to being physically comfortable, managing physical illnesses, and quick action in first aid (why is the same acceptance or reaction not there when it comes to mental health?)   Addressing the need to focus on mental health is the need of the hour. This includes speaking on the importance of maintaining sound mental health, the impact of dysfunctionality etc. |
| Please give one 'top-tip' to the person delivering this program to improve chances of success. | Engaging parents as much as possible | Keep an open mind, understanding context and parental insecurities is key in responding |
| Do you have any concerns, additional feedback or advice? | No | None |

*Note: Feedback is in reviewers own wording and has not been modified.*

Supplementary File 10: Templates for Intervention Description and Replication (TIDierR Checklists (Hoffman et al., 2014) including logic models and theory of change

The file describes the proposed whole school program to be delivered in a feasibility study (as per Hugh-Jones et al., 2022).

**Item 1: Brief Name**

SAMA: a whole school intervention for Safeguarding Adolescent Mental HeAlth.

This was co-created by the University of Leeds (UK) and the National Institute of Mental Health and Neurosciences (NIMHANS, Bangalore, India).

**Item 2: Why**

Prevention and early intervention for adolescent anxiety and depression requires a multi-faceted approach which targets known risk and protective factors at the individual, social and community level (Fusar-Poli et al., 2019). At the point of intervention development, there was no intervention in India which could be delivered in secondary schools to target rick and protective factors at the level of the individual, teachers, school climate and parents - aspects of the system that are critical to mental health promotion approaches (WHO, 2020). The SAMA intervention was developed using the steps of the ADAPT Framework for adapting complex population health interventions to new contexts (Moore et al., 2021). This included systematic reviews to identify existing effective programs which could be adapted for use in India as part of a whole school program, creation of prototypes, extensive co-adaptation / co-production with adolescents, teachers, head teachers, parents and mental health professionals in Bangalore, India. Final refinements were made to the prototypes ready for feasibility testing as per our protocol (Hugh-Jones et al., 2022).

The overarching aim of SAMA was to reduce the prevalence of clinical levels of anxiety and depression in adolescents in India. SAMA involves four interventions, to be delivered broadly simultaneously, to dynamically affect risk and protective factors operating at different levels with a focus on mental health literacy, promotion of psychological and physical safety and practical skills for self-care, stress management and managing relationships. The program was developed for delivery to Grade 9 students in Indian (14–15-year-olds). The aims and hypothesised mechanisms of change for each of the four interventions are presented in the logic models below followed by a proposed overarching theory of change for the whole school program.

**Figure S10.1 Provisional Logic Model for SAMA for YOUTH intervention**


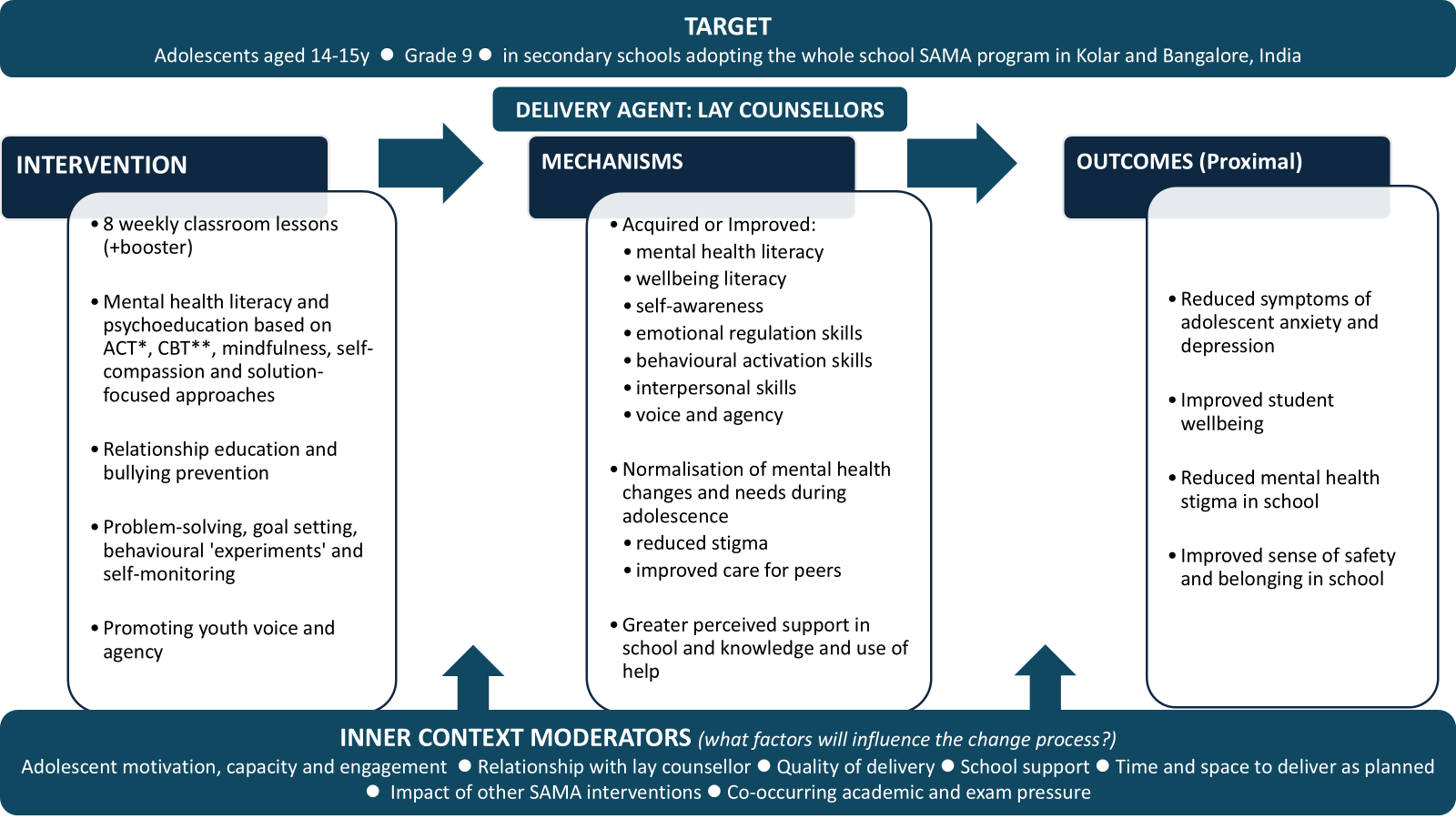


**Figure S10.2 Provisional Logic Model for SAMA for TEACHERS intervention**


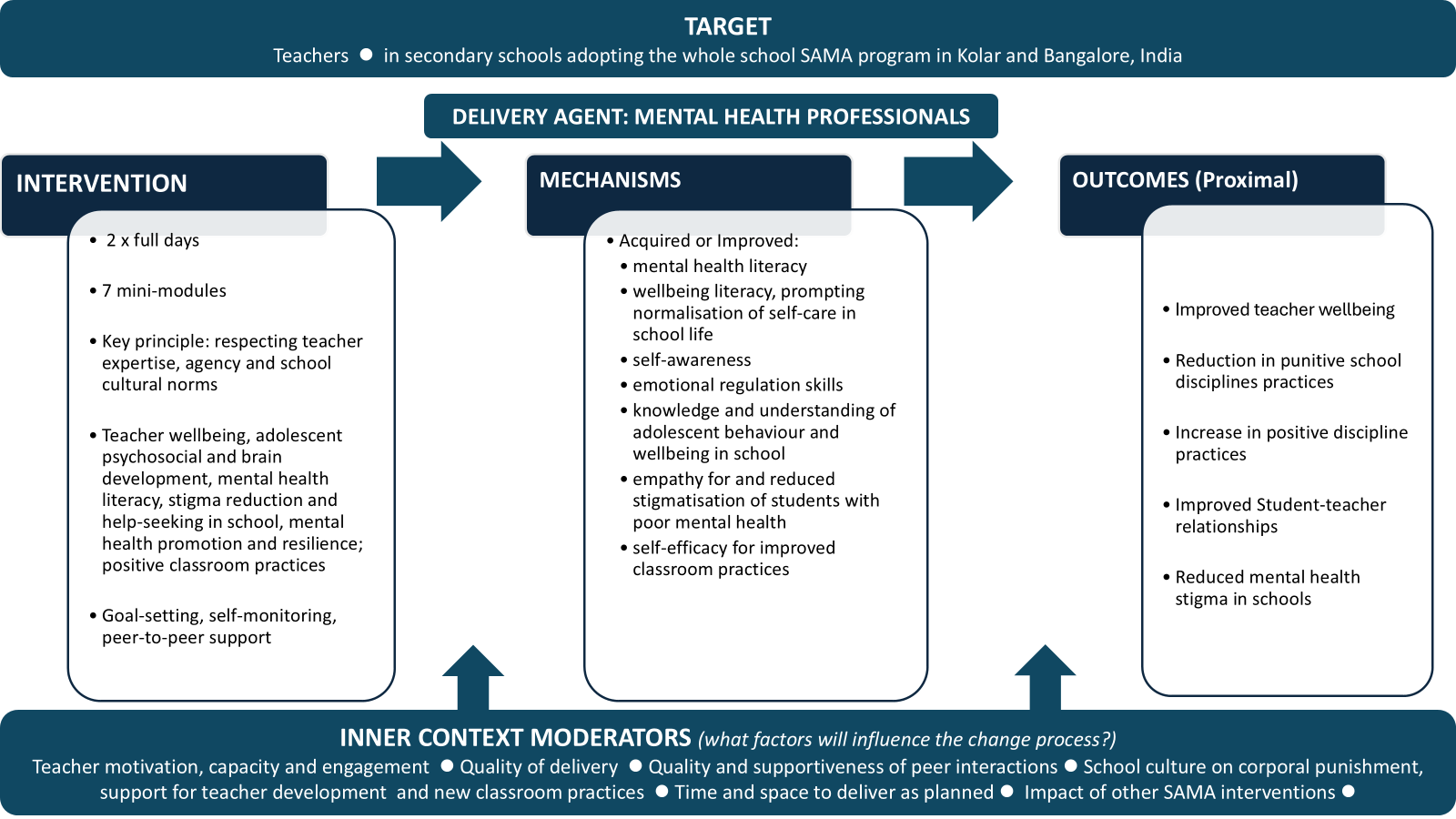


**Figure S10. 3 Provisional Logic Model for SAMA for SCHOOL CLIMATE intervention**


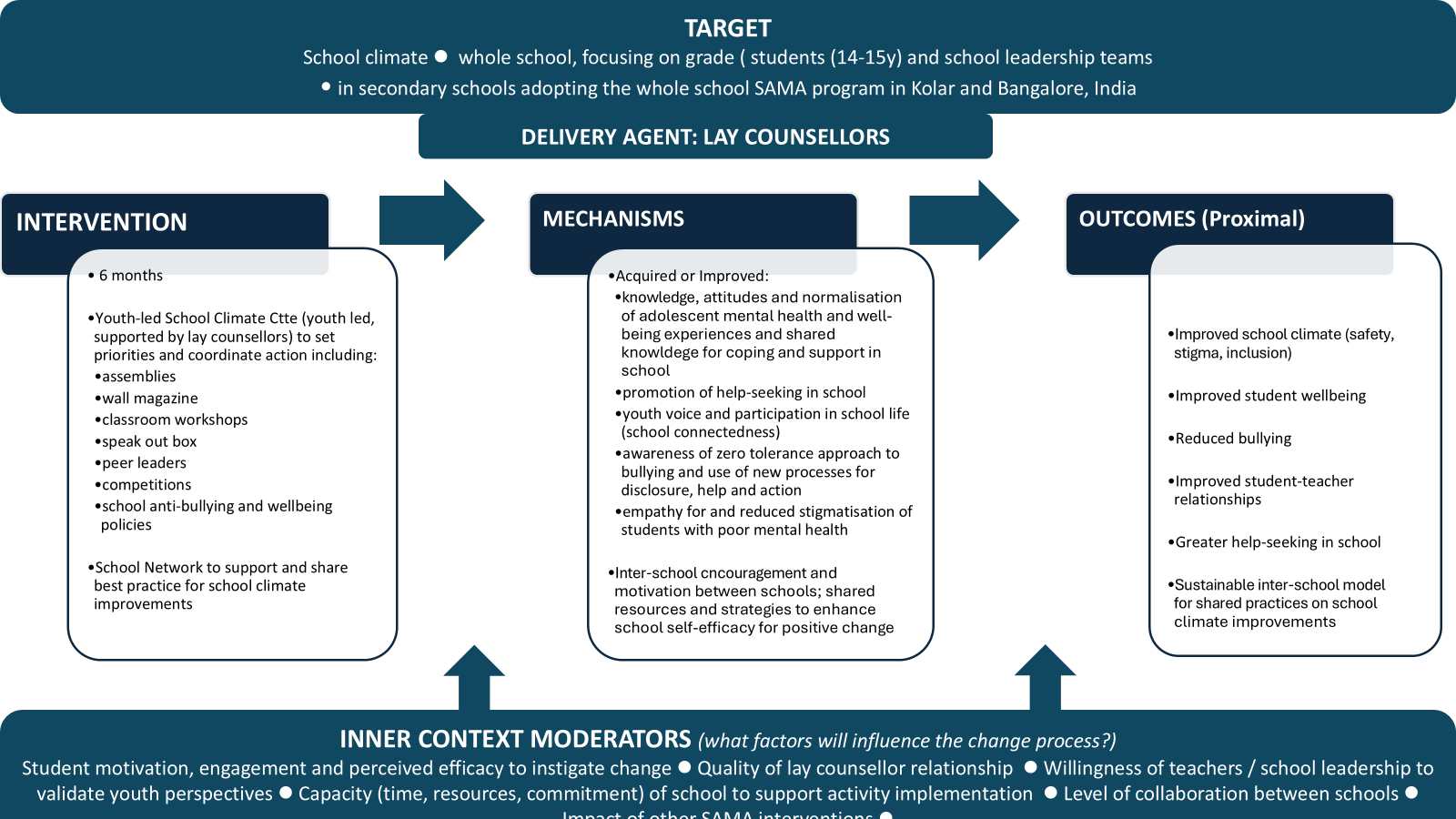


**Figure S10.4 Provisional Logic Model for SAMA for PARENTS intervention**


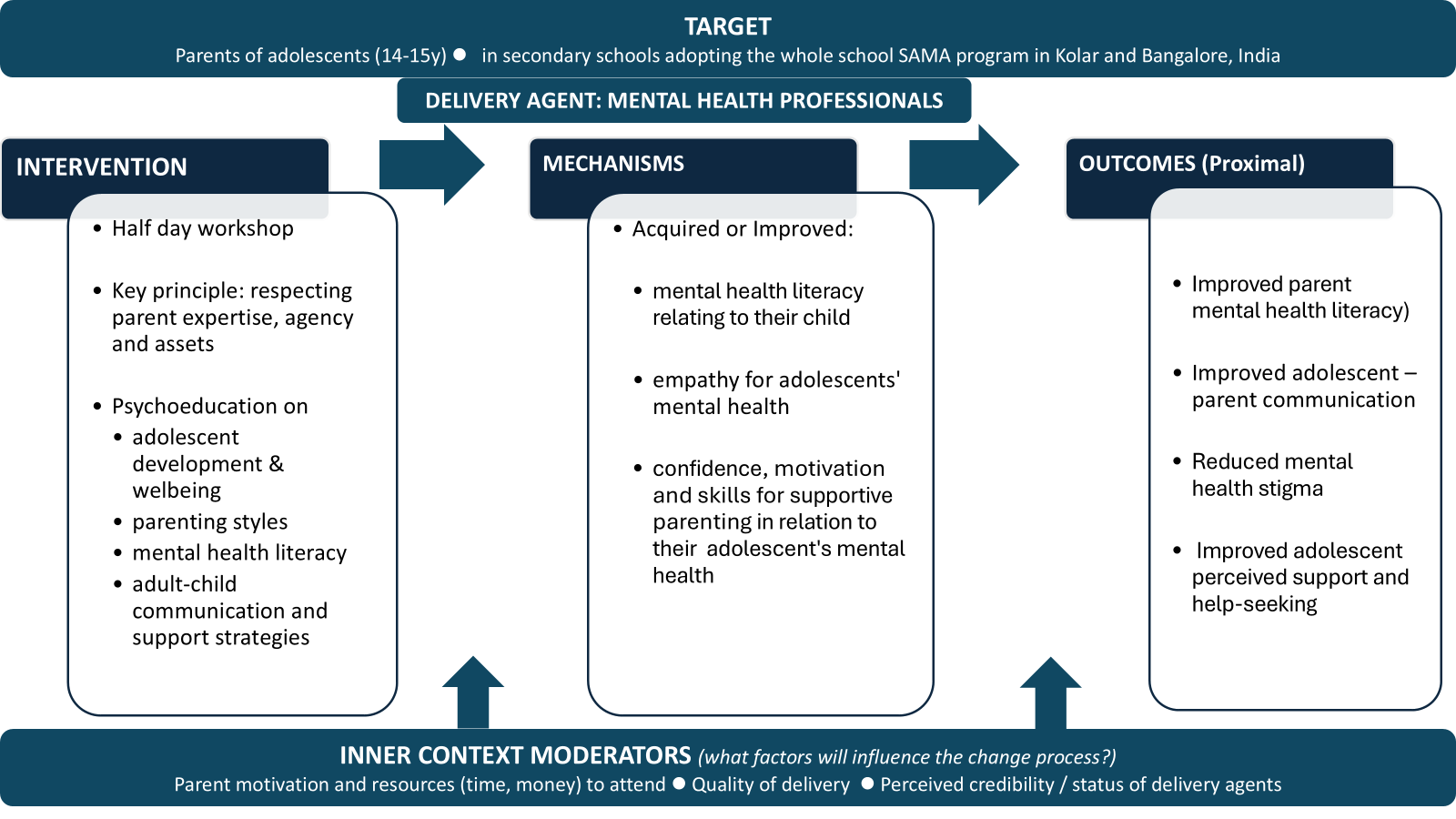


**Theory of Change**

We drew on Markham & Aveyard’s (2003) theory of health promoting schools and Ponsford et al.’s (2022) modifications.

**Figure S10.5 Provisional Theory of Change for the SAMA Whole School Program**


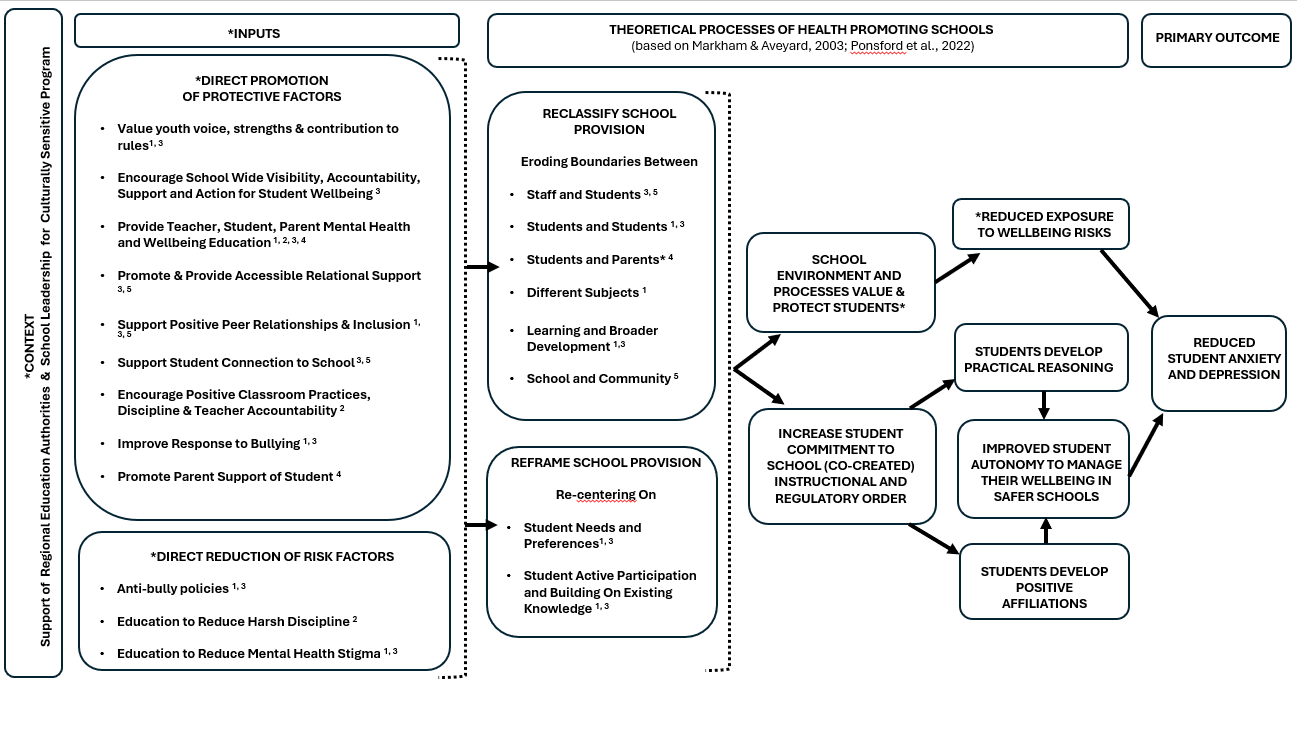


**Item 3: What Materials**

A range of paper-based materials were created for intervention content or implementation.

**Table S10.1 Examples of program content**

| **Material** | **Example** |
| --- | --- |
| Pre-implementation ‘Teaser’ posters for display in schools to create awareness and curiosity about the intervention that would be arriving in their school. |  |
| Pre-implementation Parent Newsletter explaining the whole school program to build awareness, support and engagement. | 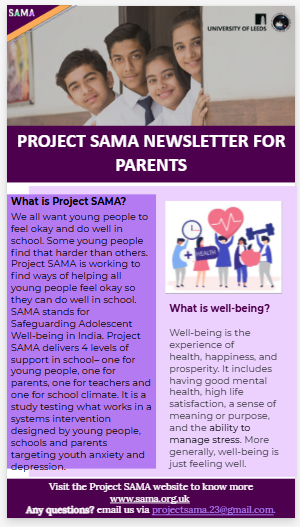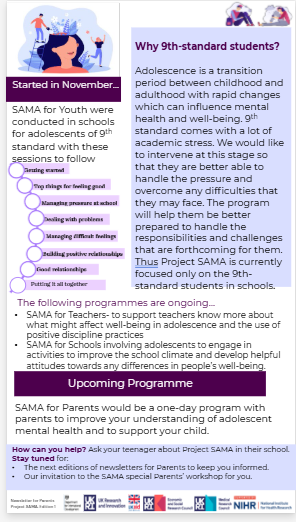 |
| Manuals were created for each of the four interventions. These are available at www.sama.org.uk or from the corresponding author of the study protocol (Hugh-Jones et al., 2022). |  |
| Manual SAMA for YOUTH | 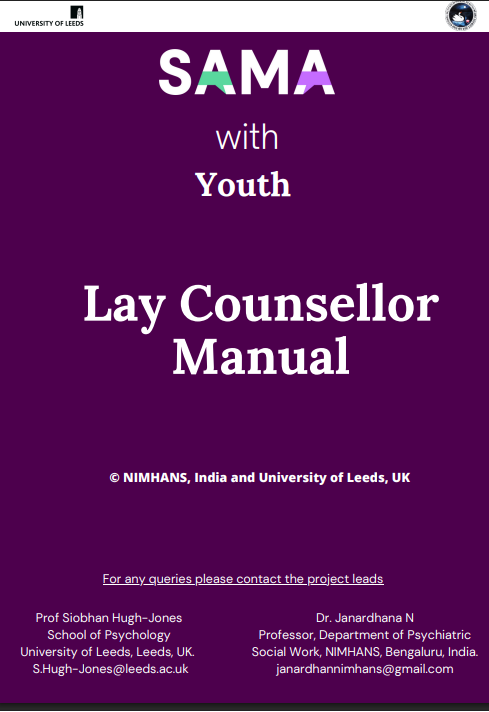 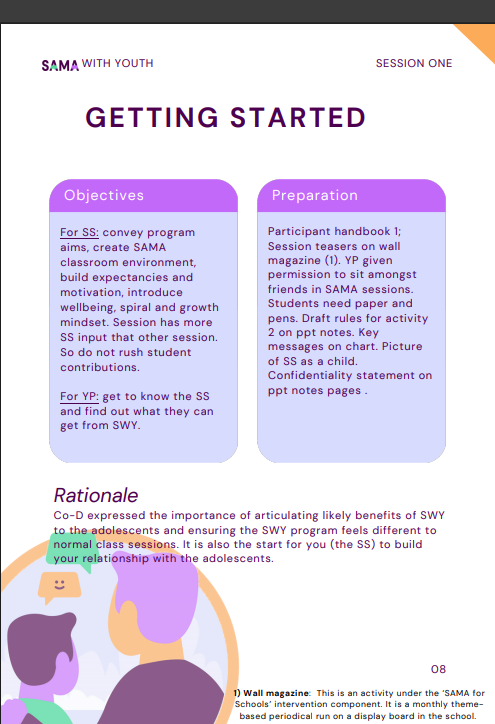 |
| In addition to the manuals, for the SAMA for YOUTH intervention, mini booklets were created for each adolescent receiving the intervention to support engagement and consolidation of the eight intervention sessions. Each adolescent to receive a plastic spiral as a representation of the SAMA wellbeing spiral which featured throughout the SAMA for YOUTH intervention. The booklet is available at [www.sama.org.uk/guides-manuals-and-tools](http://www.sama.org.uk/guides-manuals-and-tools) | 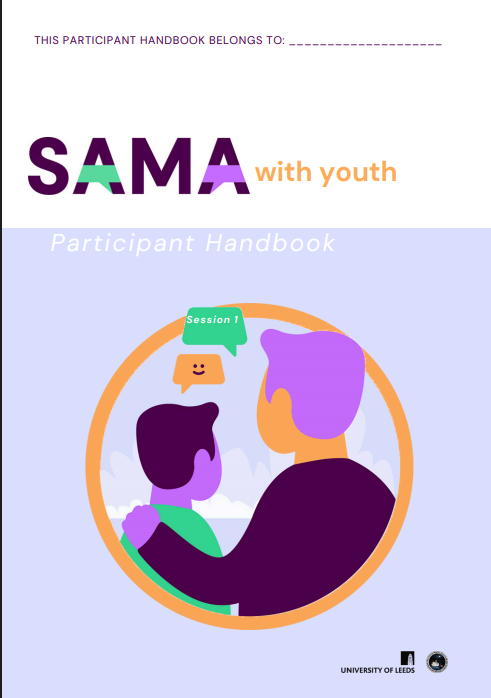 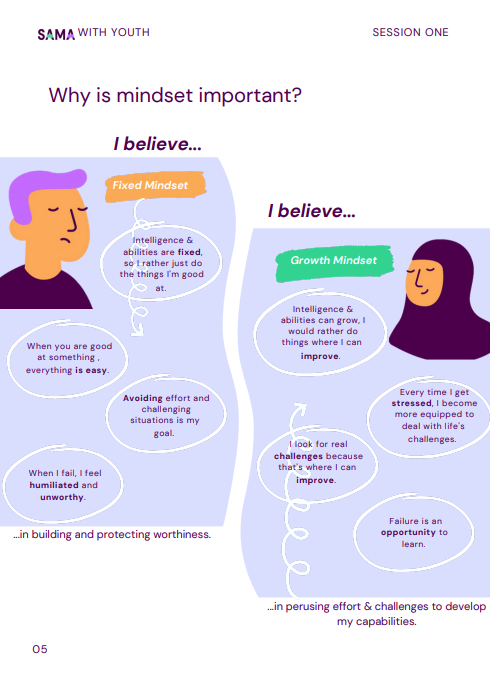  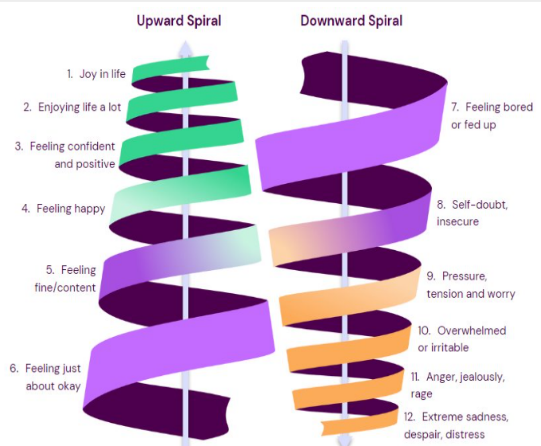 |
| Manual SAMA for TEACHERS | 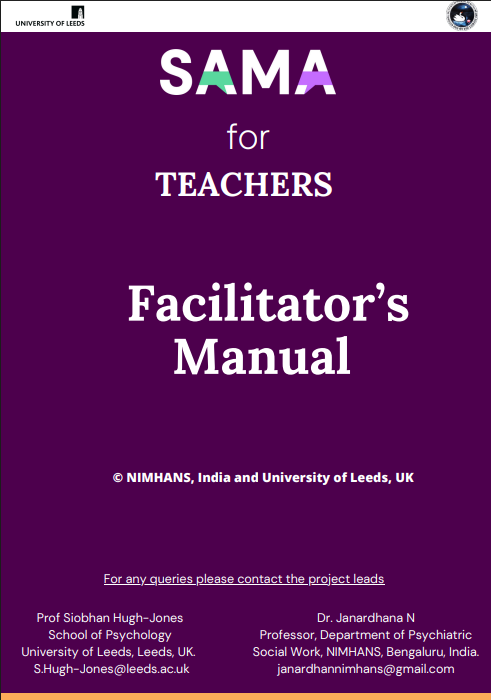 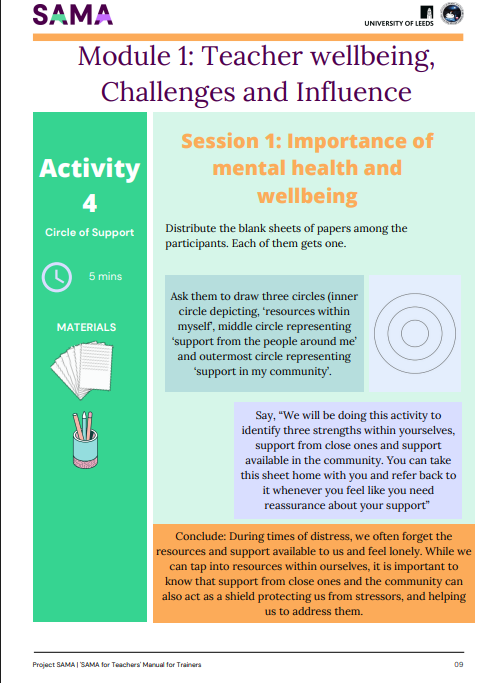 |
| Manual SAMA for SCHOOLS | 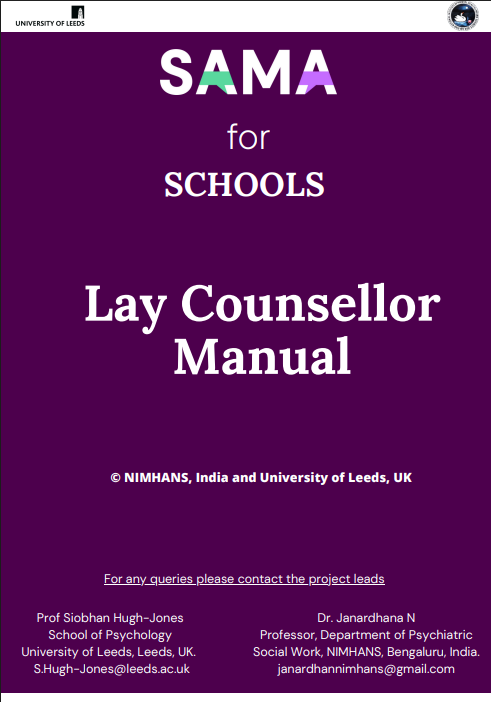 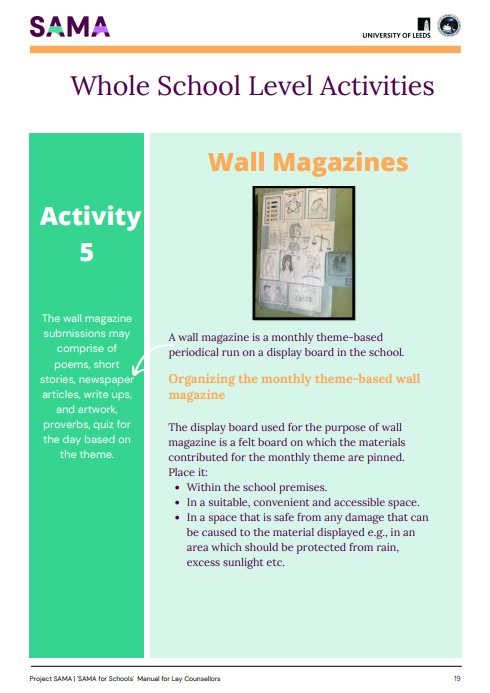 |
| In addition to the manuals, for the SAMA for SCHOOLS intervention, two policy templates were created for schools to tailor to their setting (1) Anti-bullying Policy Template and (2) Emotional Wellbeing Policy Template. These are available at [www.sama.org.uk/guides-manuals-and-tools](http://www.sama.org.uk/guides-manuals-and-tools) | 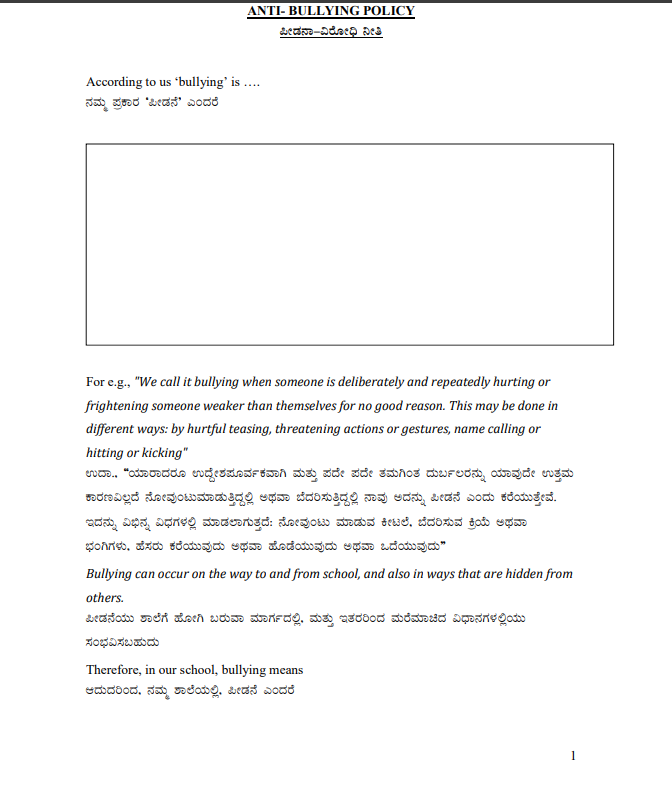 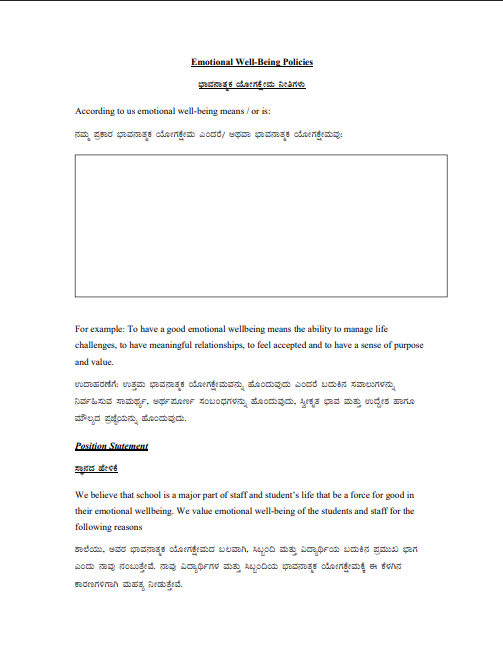 |
| Manual SAMA for PARENTS | 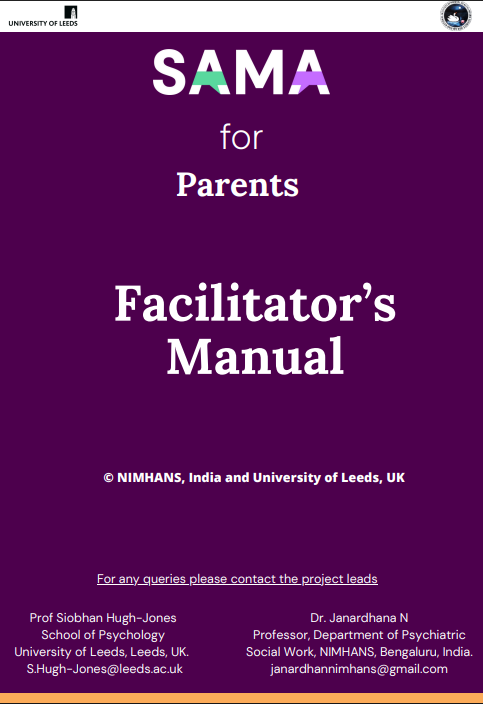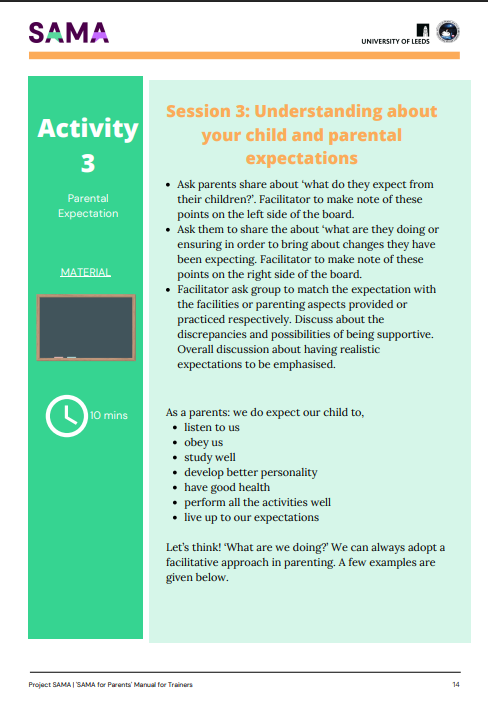 |
| Following a review of global literature and co-production with schools, adolescents, parents and mental health professionals, a protocol for the management of adolescents’ disclosure of harm, or risk of harm was developed This was manualized for training the delivery agents (lay counsellors referred to in the regional language as SAMA Snehitharus). The manual is available at [www.sama.org.uk/guides-manuals-and-tools](http://www.sama.org.uk/guides-manuals-and-tools) | 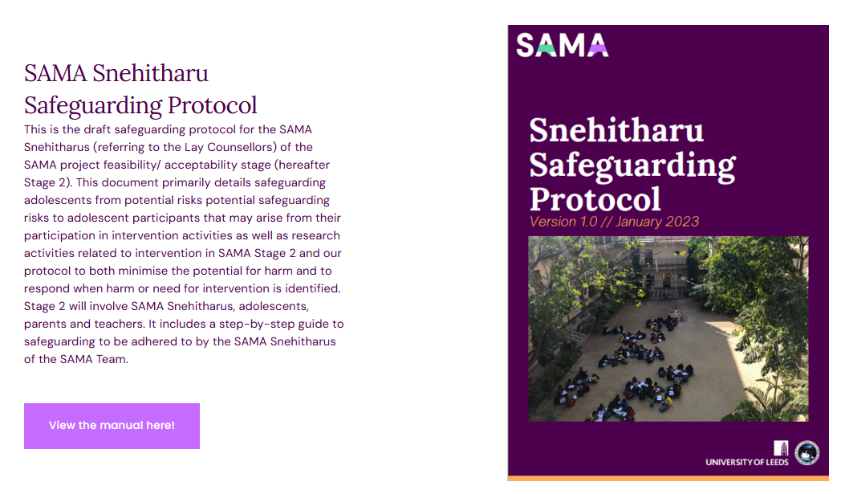 |

**Item 4: What Procedures**

The planned activities for each intervention component are available in the intervention manuals at [www.sama.org.uk/guides-manuals-and-tools](http://www.sama.org.uk/guides-manuals-and-tools). In summary:

| **Intervention Component** | **Structure** | **Content** | **Indicative Activities** |
| --- | --- | --- | --- |
| SAMA for YOUTH | 8 x weekly one hour classroom session + potential extension lessons + one booster | 1. Getting Started 2. Top Tips for Feeling Good 3. How to Manage Pressure at School 4. Dealing With Problems 5. Managing Difficult Feelings 6. Building Positive Relationships (Accepting others) 7. Building Positive Relationships (Maintaining good relationships) 8. Putting it all together | Quizzes  Role-Play  Small group discussions  Ranking activities  Art / creative activities  Vignettes  Problem-Solving  Reviewing videos  Making plans |
| SAMA for TEACHERS | 7 x 90minute sessions every 2-3 weeks | 1. Teacher wellbeing and challenges 2. Understanding mental health and mental health disorders 3. Destigmatising mental health problems 4. Encouraging and promoting help-seeking 5. Mental health promotion & wellbeing activities 6. Building resilience for wellbeing 7. Positive discipline and classroom management | Peer discussion  Vignettes  Problem-solving  Personal reflection  Making plans |
| SAMA for SCHOOLS | 6-12 months | 1. Whole school level activities   (i) Awareness raising (of youth wellbeing, agency and engagement)  (ii) Wall Magazine (mental health knowledge, youth-led)  (iii) School climate team (planning whole school activities)  (iv) Wellbeing and Anti-bullying policies  (v) Competitions (for youth expression)  (vi) Speak out box (to raise personal concerns)   1. Group (Class) level activities (fortnightly)   (i) Adolescent nominated topic- based workshops | Assembly announcements  Team meetings  Creative activities (to create wall magazine)  Advertising competitions  Making plans  Promoting the Speak Out box  Completing policies  Peer discussion  Vignettes  Role-play |
| SAMA for PARENTS | One day workshop | 1. Program orientation 2. Exploring parental understanding of mental health 3. Understanding your child and your expectations 4. Adolescent mental health & addressing stigma 5. Identification of mental health needs 6. Impact of poor mental health and ways to help 7. Tips and commitment to practice | Group discussion  Reviewing vignettes  Personal reflections  Quizzes  Making plans |

**Item 5: Who provided**

It was planned that SAMA for YOUTH and SAMA for SCHOOLS would be delivered by trained lay counsellors who had at least a bachelor’s degree in any field; were between the age of 25-30; were resident close to the schools; and had evidence of skills in key areas (working with adolescents, communication, professional conduct, mental health knowledge, and appreciation of confidentiality and safeguarding). Candidate lay counsellors would be invited onto the SAMA Foundational Training program where further assessment of their suitability and competence as delivery agents could be assessed. This would include evidence of a passion for working with young people and schools, an ability to deliver intervention content correctly, energetically and according to given values (rather than personal values), as well as an ability to communicate respectfully with peers and professionals.

Lay counsellor training and support was designed and delivered by NIMHANS, and would include:

**1. Foundational Training:** *Phase 1* would include orientation to the project and the role; working with and for adolescents (e.g. age-appropriate expectations; youth mental health literacy); working in schools (e.g. understanding school governance, coordinating with headteachers); values of SAMA (e.g. respect, curiosity, acceptance of difference, the rights of adolescents); ethics and SAMA safeguarding protocol; generic skill development (e.g. record keeping, communicating with intervention team, building relationships in schools). *Phase 2* would focus on intervention content, delivery procedures, and where tailoring and flexibility was possible. This would include extensive experiential practice in delivering the SAMA for YOUTH and SAMA for SCHOOLS sessions, as well as role-playing difficult or high-risk situations (e.g. disclosure of bullying; teacher complaints; being asked to take on unplanned activities in school). Foundational training would be delivered over a period of two weeks.

**2. On-the-job training:** It was planned that one lay counsellor would be assigned to each study school for the duration of intervention delivery. Once lay counsellors began their work in schools, there would be ongoing support and mentoring with the project team based in NIMHANS as well as peer support (between the lay counsellors themselves). Lay counsellors would be supported in the first two sessions of SAMA for YOUTH by NIMHANS project assistants who were mental health professionals.

**3. Support Sessions:** The project team would check in weekly with lay counsellors to debrief on their week, clarify any uncertainties, review intervention delivery, consistent record keeping and any emerging barriers or difficulties. This would be either via WhatsApp video or in person meetings in schools. Emergency support would be available 24/7 with a nominated clinical psychologist at NIMHANS for cases regarding urgent mental health crises or safeguarding.

**4.** **Booster training:** Three months after intervention start, lay counsellors would receive group booster training. This would explore challenges faced by the lay counsellors, reminders of safeguarding procedures, revisiting intervention values and content and boosting lay counsellors motivation and confidence in their roles.

It was planned that lay counsellors were employed full-time as delivery agents and would be paid a wage aligned with regional salaries.

SAMA for TEACHERS and SAMA for PARENTS would be delivered by mental health professionals from NIMHANS. The co-production phase identified that lay counsellors would not been viewed by stakeholders as suitably qualified, or of the correct status, to deliver these intervention components to teacher or parents. These mental health professionals would be from the SAMA project team who had been directly involved in generating the intervention prototypes and no so additional training would be required. These team members would not be paid separately for delivery of these intervention components.

**Item 6: How delivered**

All components of the whole school program would be delivered in person either by lay counsellors or NIMHANS mental health professionals, on school premises, during school hours and to groups (i.e. classes of adolescents, groups of parents, groups of teachers). Scheduling of intervention delivery would be in consultation with school leadership, and would avoid exam periods, holidays and festivals.

**Item 7: Where**

The program was delivered in two regions of the state of Karnataka India; Kolar (rural) and Bangalore (urban). Two school types received the program: government aided schools and private schools. Adolescents in grade 9 (14-15y) received SAMA for YOUTH and SAMA for SCHOOLS, although students from other grades were not excluded from activities linked to the latter if they were keen to take part. This was to foster broader school engagement and ownership of the school climate component.

**Item 8: When and how much**

The whole school program was designed to be delivered over a minimum of six months and a maximum of one full academic year. SAMA for SCHOOLS was the planned to be longest in duration and was designed to run for at least six months and preferably for one full academic year. There was flexibility in whether the other three interventions would be delivered sequentially or simultaneously, depending on school preference and logistics. It was planned that SAMA for YOUTH would involve 8 weekly sessions and one booster session. SAMA for TEACHERS would involve 7 x 90-minute sessions delivered every 2-3 weeks, and SAMA for PARENTS would involve a one-day workshop. See Hugh-Jones et al. (2022) for study design protocol.

**Item 9: Tailoring**

We planned to encourage delivery agents to adapt each intervention once they had begun to learn about the school, the students and the staff. Each school has its own culture, processes and ways of working. Adaptation means achieving a good fit between the intervention and the context and requires empowering teams to innovate ‘on the job’ (within-systems innovation), and to address missed or non-prioritised key implementation determinants during implementation (Holdsworth et al., 2020; Kirk et al., 2020; Moore et al., 2021). Local adaptation has also been suggested as a way to ensure interventions are sustained (Moore et al., 2021). We planned continual monitoring and record-keeping of any adaptations via online templates completed weekly by delivery agents, and reviewing during weekly check-in with the research team.

Training to delivery agents was planned to advise on the core intervention components which had to be delivered, and where there was flexibility to tailor the intervention to different groups, settings and circumstances. For example, adaptation could include: hosting the classroom sessions in smaller groups, modifying role-plays if participants are reluctant, increasing or decreasing the level of peer discussion among teachers depending on fatigue, pacing and staging the school climate activities around other planned school events. As the schools and our research team attempt to adapt and implement SAMA, they will generate practical knowledge and practice -based evidence which can be translated back into our final implementation framework (van der Laan & Boenink, 2015).

**Item 10: Modifications**

following the feasibility study, we plan to use the Stirman et al. (2019) Reporting Adaptations and Modifications Framework (FRAME) to document modifications to the intervention made during implementation (as per Item 9).

**Item 11 & 12: How well**

Not applicable

**REFERENCES**

Fusar‐Poli, P., Correll, C. U., Arango, C., Berk, M., Patel, V., & Ioannidis, J. P. (2021). Preventive psychiatry: a blueprint for improving the mental health of young people. *World Psychiatry*, *20*(2), 200-221.

Holdsworth, L. M., Safaeinili, N., Winget, M., Lorenz, K. A., Lough, M., Asch, S., & Malcolm, E. (2020). Adapting rapid assessment procedures for implementation research using a team-based approach to analysis: a case example of patient quality and safety interventions in the ICU. *Implementation Science, 15*(1), 12. doi:10.1186/s13012-020-0972-5

Kirk, M. A., Moore, J. E., Wiltsey Stirman, S., & Birken, S. A. (2020). Towards a comprehensive model for understanding adaptations’ impact: the model for adaptation design and impact (MADI). *Implementation Science, 15*(1), 56. doi:10.1186/s13012-020-01021-y

Moore G, Campbell M, Copeland L, Craig P, Movsisyan A, Hoddinott P et al. Adapting interventions to new contexts—the ADAPT guidance BMJ 2021; 374 :n1679 doi:10.1136/bmj.n1679

van der Laan, A. L., & Boenink, M. (2015). Beyond bench and bedside: disentangling the concept of translational research. *Health Care Anal, 23*(1), 32-49. doi:10.1007/s10728-012-0236-x

World Health Organization. (2020). *Guidelines on mental health promotive and preventive interventions for adolescents: helping adolescents thrive*. World Health Organization.

Supplementary File 11: Guidance for Reporting Involvement of Patients and the Public (GRIPP 2) Staniszewska et al., 2017)

**Table S11:1 GRIPP2 short form** (pages numbers to be added on final typeset version)

| **Section and topic** | **Item** | **Reported on page No** |
| --- | --- | --- |
| 1: Aim | Report the aim of PPI in the study |  |
| 2: Methods | Provide a clear description of the methods used for PPI in the study |  |
| 3: Study results | Outcomes—Report the results of PPI in the study, including both positive and negative outcomes |  |
| 4: Discussion and conclusions | Outcomes—Comment on the extent to which PPI influenced the study overall. Describe positive and negative effects |  |
| 5: Reflections/critical perspective | Comment critically on the study, reflecting on the things that went well and those that did not, so others can learn from this experience |  |

PPI=patient and public involvement

Staniszewska, S., Brett, J., Simera, I., Seers, K., Mockford, C., Goodlad, S., ... & Tysall, C. (2017). GRIPP2 reporting checklists: tools to improve reporting of patient and public involvement in research. BMJ *358*.
